# On the origin of animals and placental mammals: a critique of literalist readings of the fossil record

**DOI:** 10.1093/molbev/msag211

**Published:** 2026-08-19

**Authors:** Sandra Álvarez-Carretero, Ziheng Yang, Philip C J Donoghue, Mario dos Reis

**Affiliations:** Department of Genetics, Evolution and Environment, University College London, London WC1E 6BT, UK; Department of Genetics, Evolution and Environment, University College London, London WC1E 6BT, UK; Bristol Palaeobiology Group, School of Earth Sciences, University of Bristol, Bristol BS8 1TQ, UK; Centre for Evolutionary and Functional Genomics, School of Biological and Behavioural Sciences, Queen Mary University of London, London E1 4NS, UK

**Keywords:** molecular clock, fossil record, Bayesian model selection, placental mammal, animal origins

## Abstract

The fossil record is incomplete, as evidenced by the pervasive presence of ghost lineages throughout the Tree of Life. For example, across placental mammals, at least 720 Myr of basal lineages are ghost lineages, that is, lineages that have left no fossil evidence of their past history. In contrast, some studies have suggested that the fossil record is a faithful temporal archive of evolutionary history and thus the times of diversification of clades must be close to the ages of their oldest fossils. Such literalist interpretations have been contradicted by analysis of molecular datasets which, in many cases, indicate that groups including placental mammals and animals may have originated at times substantially older than their fossil records. Some of those studies have further argued that, in the case of animals and placental mammals, molecular clocks are uninformative, suffer from characteristic pathologies, and thus cannot distinguish between recent and ancient hypotheses of diversification. Here, we reexamine these two cases and show, using Bayesian model selection theory, that the explosive diversification models previously proposed for animals and placental mammals have a posterior probability of ∼0. We show the characteristic pathologies purportedly discovered do not exist, highlight errors in previous analyses, and provide advice on best practice for molecular-clock dating analysis.

“When we see a species first appearing in the middle of any formation, it would be rash in the extreme to infer that it had not elsewhere previously existed.”

Charles Darwin

On the Origin of Species

## Introduction

Evolutionary timescales are foundational to evolutionary biology, providing rates of evolution and dates from which to test hypotheses of diversification, coevolution, and competition. Traditionally, evolutionary history has been the domain of palaeontology, with the antiquity of living lineages inferred from their oldest fossil records. However, most lineages have little or no fossil record, and molecular-clock theory was developed to estimate evolutionary timescales from the molecular distances between species and fossil-based constraints on the timing of their divergence (Zuckerkandl and Pauling 1965). Molecular-clock methods have undergone extraordinary development to incorporate uncertainty in molecular rate variation and fossil calibration uncertainty, exploiting genome scale data from an increasing diversity of species (Donoghue and Yang 2016). Consequently, molecular-clock methods are widely employed in evolutionary biology and epidemiology, underpinning diverse research questions concerning the evolutionary rates and divergence times (dos Reis et al. 2016). In contrast, Budd and Mann (2020a) argue that, for some clades (such as animals or placental mammals), the fossil record should be read more or less literally and admonish molecular-clock studies for failing to test the hypothesis that the fossil record is a faithful temporal archive of evolutionary history (Budd and Mann 2024).

**Figure 1:**
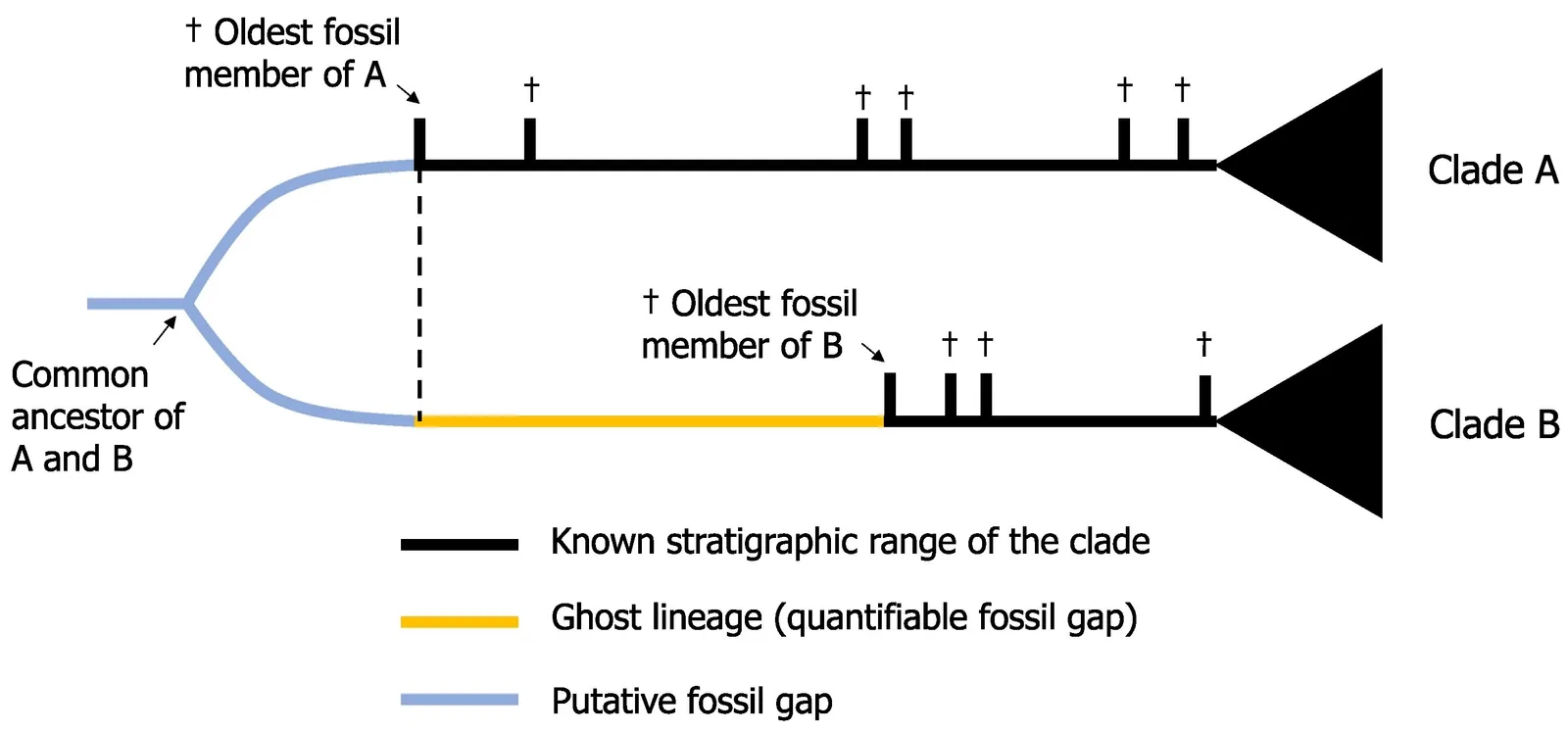
Gaps in the fossil record. Sister clades can be used to estimate the length of fossil gaps if one of the clades has older fossil members. The length of this fossil gap, known as a ghost lineage (shown in orange), is simply the age difference between the oldest fossil members of the two clades. However, an additional fossil gap must also exist beyond the oldest fossil member of the crown group. This gap (shown in blue) is hard to quantify as absence of fossils cannot be interpreted as absence of the crown group. Notorious examples of crown gaps include bilaterians, placental mammals, birds, and flowering plants.

The fossil record is an invaluable archive of evolutionary history, providing insight into the nature of extinct lineages, expanding on understanding of the historical biogeography of extant lineages, informing on the sequence in which body plans were assembled, and, in particular, providing minimum constraints on clade ages that are integral to disambiguating rates and times in the calibration of molecular clocks (Patterson 1981). However, the fossil record is an incomplete temporal archive of evolutionary history. The stratigraphic ranges of fossils always underestimate the true ages of clades because the fossil record is limited (largely) to morphology. Lineage divergence is a genomic phenomenon that is manifest in the fossil record only after derived lineages have accrued morphological distinctions from one another and from their ancestral lineage (Donoghue and Yang 2016). This expected temporal lag in the fossil evidence of lineage divergence is exacerbated by the nature of the rock record in which fossils are entombed, which is a nonuniform record of geography and environments across geologic time (Holland 2016). Even for groups with a rich fossil record that has been intensively sampled and studied, which yield rich remains from which credible morphological phylogenies may be derived, there is intrinsic evidence that their temporal distribution is a poor record of their evolutionary history. This is clear from inferences of ghost lineages, ie the minimum estimate of incompleteness of the fossil record based on the stratigraphic ranges of sibling lineages which, by definition, emerged simultaneously from a common ancestor (Fig. 1). For example, O’Leary et al. (2013) identified 61 ghost lineages among the basal branches that they sampled in placental mammal phylogeny, equating to a total of over ∼780 Myr of evolution with no record, according to Fig. 1 in their paper (see section “The evolutionary gap in the fossil record of placental mammals” in Material for details). Ghost lineages provide an uncontroversial quantification of gaps in the fossil record (Norell and Novacek 1992), but they are minimum estimates since there is no reason to believe that the older records among sibling lineages accurately represent clade age. Hence, there is a long tradition of applying statistical methods to estimate true stratigraphic ranges for species or clades (Paul 1982; Strauss and Sadler 1989; Marshall 1990; Sadler et al. 2009; Silvestro et al. 2021). For example, using a non-homogenous birth–death process with fossil sampling, Wilkinson et al. (2011) inferred a median age for Crown Primates between 68.7 and 63.6 Ma (depending on diversification model used), with a 97.5% upper quantile for the crown group age between 88.9 and 88.6 Ma.

**Figure 2:**
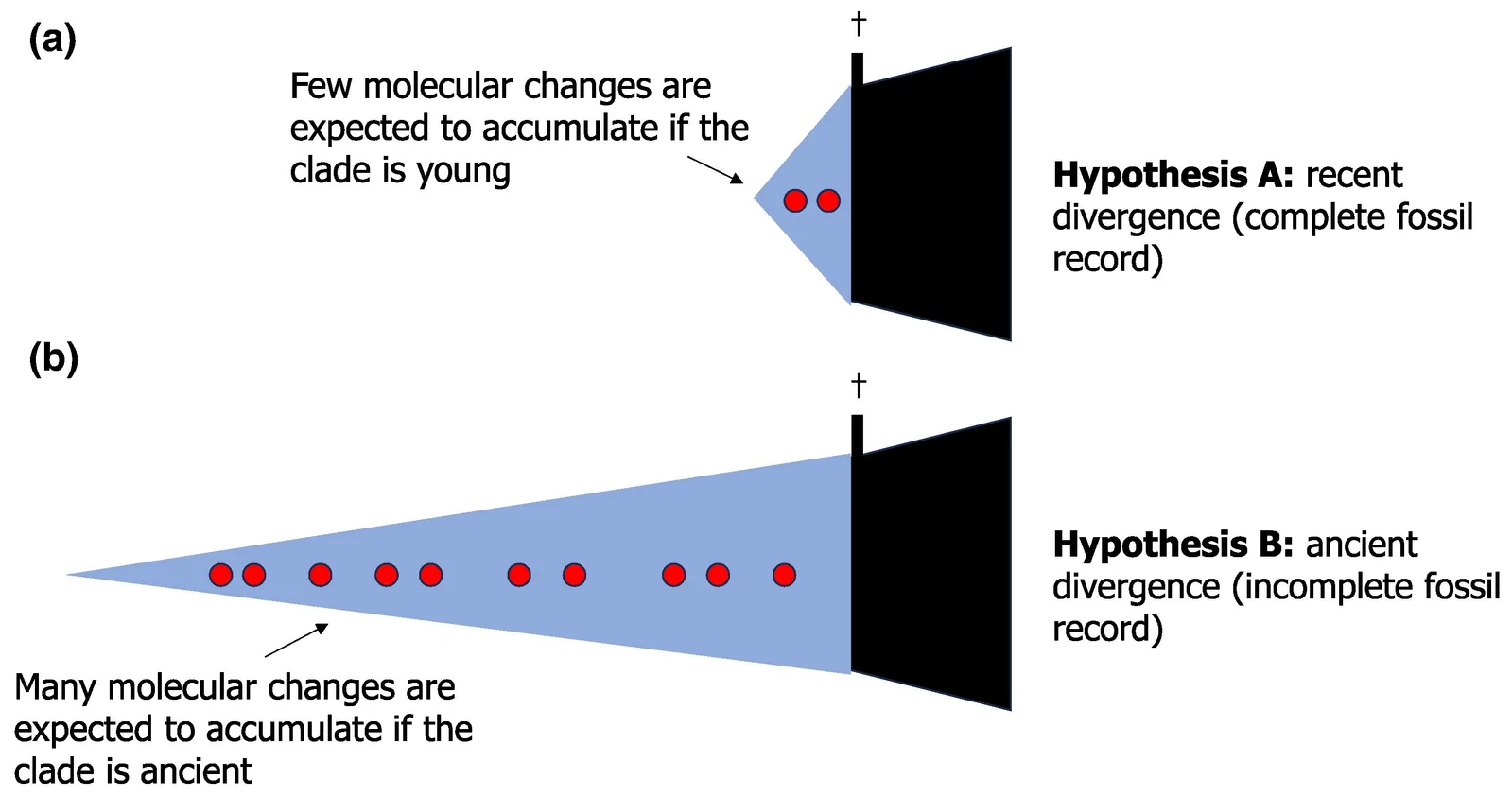
Estimation of fossil gaps using molecular clocks. The genomes of organisms accumulate changes with the passage of time. This fact is leveraged by Bayesian molecular-clock methods, which integrate over fossil uncertainty and rate variation resulting from the accumulation of genomic changes to infer the ages of clades. A young crown group (a) will have accumulated a smaller number of genomic changes (shown as red dots) compared to an old crown group (b). Bayesian model selection theory can be used to distinguish between these two scenarios by integrating the likelihood of molecular branch lengths over the joint prior of times and rates.

The absence of fossil evidence for a group can have two possible explanations (Fig. 2): the group is young, and thus the corresponding fossil record is a reliable indicator of the group's age (Fig. 2a), or the group is old and the earliest period of their evolutionary history is unrepresented or unrecognizable in the fossil record (Fig. 2b). Both scenarios can be distinguished using molecular-clock methods because old groups would have accumulated a substantial number of molecular substitutions, leading to long ancestral molecular branches. Bayesian molecular-clock methodologies have emerged as the state-of-the-art as they allow integration of fossil and molecular rate uncertainties in the inference procedure (Pett and Heath 2020) and thus allow assessments on whether crown groups are close to their oldest fossil representatives, conditioned on the rate model used and the configuration of fossil calibrations throughout the phylogeny being studied.

**Figure 3:**
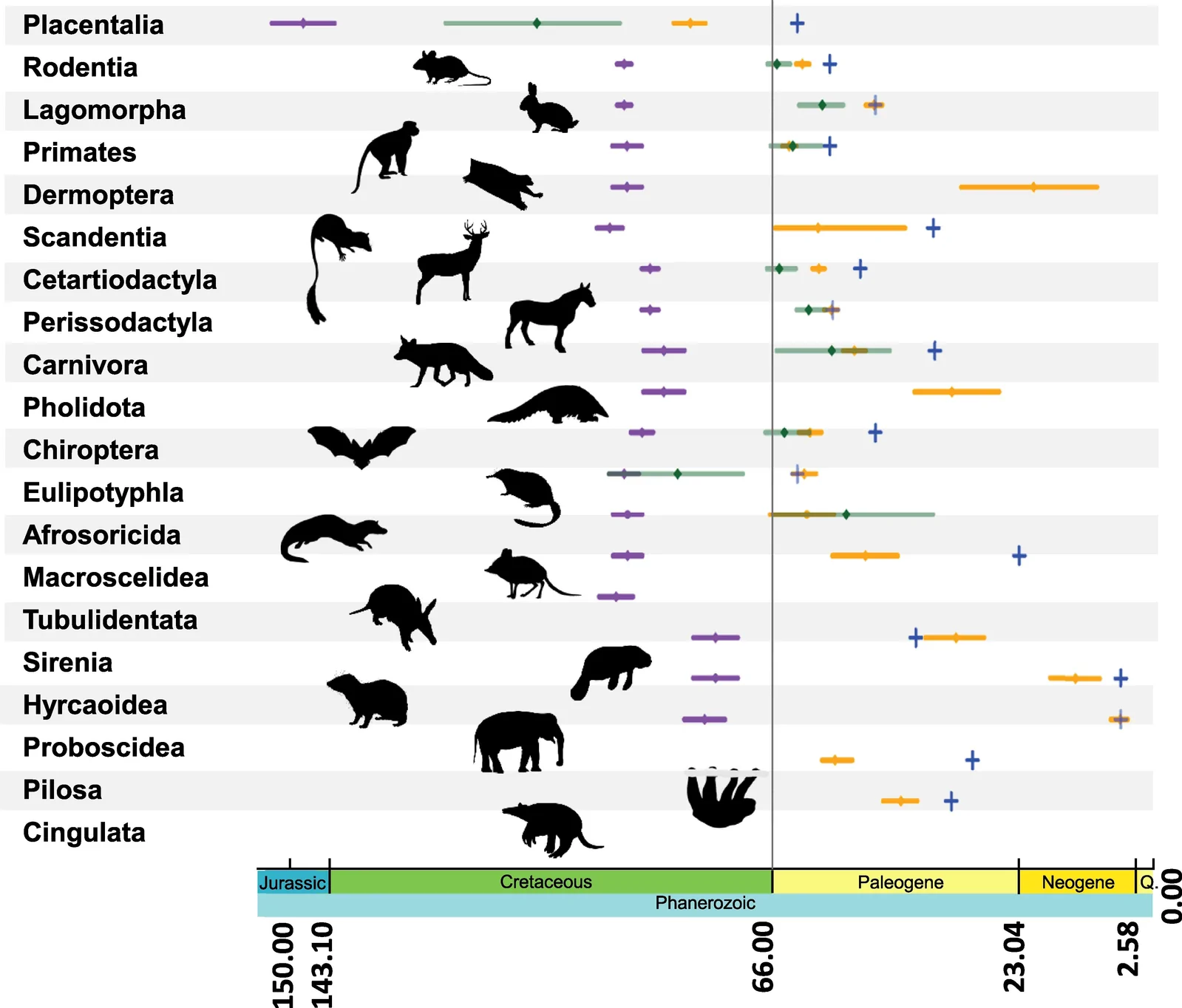
Comparison of divergence–time estimates for Placental mammals between the studies of Bininda-Emonds et al. (2007), Álvarez-Carretero et al. (2022), and Foley et al. (2023). The oldest fossils used in Álvarez-Carretero et al. (2022) to set up the minimum age calibrations are indicated with a blue dagger. The posterior time estimates by Foley et al. (2023) are in green, those estimated by Álvarez-Carretero et al. (2022) are shown in orange, and those in purple are the penalized likelihood estimates by Bininda-Emonds et al. (2007). Dots show the mean divergence–time estimate, while bars show the uncertainty in the estimates (95% confidence/credibility intervals). When a particular time estimate was not available, the corresponding colored bar and dot are not shown. Please note that Foley et al. (2023) reported various time estimates under different scenarios; we plot relevant time estimates corresponding to analysis “Benton 2009 under the IRM model” listed in Table S10 and shown in Fig. 4b. Taxon silhouettes are freely accessible via Phylopic. The link to each individual image is given in the Material.

Early non-Bayesian clock methods were not well-suited to study diversification timings because they usually used fossil calibrations as point calibrations without uncertainties and could not integrate over evolutionary rate uncertainties (Graur and Martin 2004; Reisz and Müller 2004). Application of these early methods resulted in extremely ancient divergence–time estimates for many groups, including a 1 billion year estimate for divergence of animals (Runnegar 1982; Wray et al. 1996; Wang et al. 1999) and a deep Cretaceous estimate for the divergence of placental mammals (Hedges et al. 1996; Bininda-Emonds et al. 2007). Application of the latest Bayesian methods using genomic data has reconciled fossil records with estimated divergence times. For instance, recent molecular timescales for mammals (Álvarez-Carretero et al. 2022; Foley et al. 2023) have estimated crown placental orders to have diverged after the end-Cretaceous (K-Pg) mass extinction, close to their fossil records, in contrast to earlier non-Bayesian methods that inferred ancient ordinal divergences deep within the Cretaceous (Fig. 3). However, molecular dating studies consistently agree on a Cretaceous origin of placental mammals because the molecular branch lengths linking this ancestor to the ordinal crown groups are long. Reconciling the placental ancestor with a post-Cretaceous origin would require concertinaing these long branches into a small time interval, resulting in an implausible, viruslike rate of molecular evolution (Springer et al. 2013). De novo estimates of mutation rates in mammals by pedigree sequencing indicate rates in the same order of magnitude as the phylogenetic estimates (Bergeron et al. 2023).

However, despite the clear gaps in the fossil record, several studies have sought to interpret the record of specific clades literally, assuming that the ages of origination of particular groups are the same as the age of their oldest fossils (Magallón 2010; O’Leary et al. 2013). The most prominent of these have been by Budd and Mann (2020a, 2020b, 2024), who have argued that molecular clocks suffer from “characteristic pathologies” that consistently result in overestimation of the ages of particular groups.

For example, Budd and Mann (2024) examine studies on the origin of animals (dos Reis et al. 2015) and placental mammals (Álvarez-Carretero et al. 2022), concluding that both groups originated much later than suggested by the molecular-clock estimates. In their reanalysis, Budd and Mann (2024) show that molecular-clock analyses can provide very young estimates for animals and placentals by placing younger maximum bounds on the prior of the ages of those groups. They argue that molecular timescales are thus uninformative about times of divergence because early or late divergence times can be obtained simply by the choice of maximum bounds.

Here, we directly address these criticisms by developing a Bayesian model selection test of diversification scenarios. We show that molecular-clock analyses can distinguish between late and early divergences because early divergences are accompanied by long molecular branches that cannot be accommodated within short time spans. We apply our new approach to the timing of origin of animals and placental mammals and show that the explosive diversification scenarios proposed by Budd and Mann (2024) provide a poor fit to the data and are thus decisively rejected by the Bayesian model selection approach. We also show that the analysis of mammals undertaken by Budd and Mann (2024) suffers from a gross error in the numerical optimization of branch lengths during Bayesian analysis, invalidating their results and conclusions. Our results indicate that enforcing more or less literal interpretations of the fossil record, in which the origins of groups are assumed to be close to their oldest fossils, is unwarranted and unjustified. Instead, Bayesian tests of competing diversification models of evolution can be used to formally assess the congruency between the fossil record and molecular data in inferring evolutionary timescales.

## A Bayesian model test of diversification timings in animals

**Figure 4:**
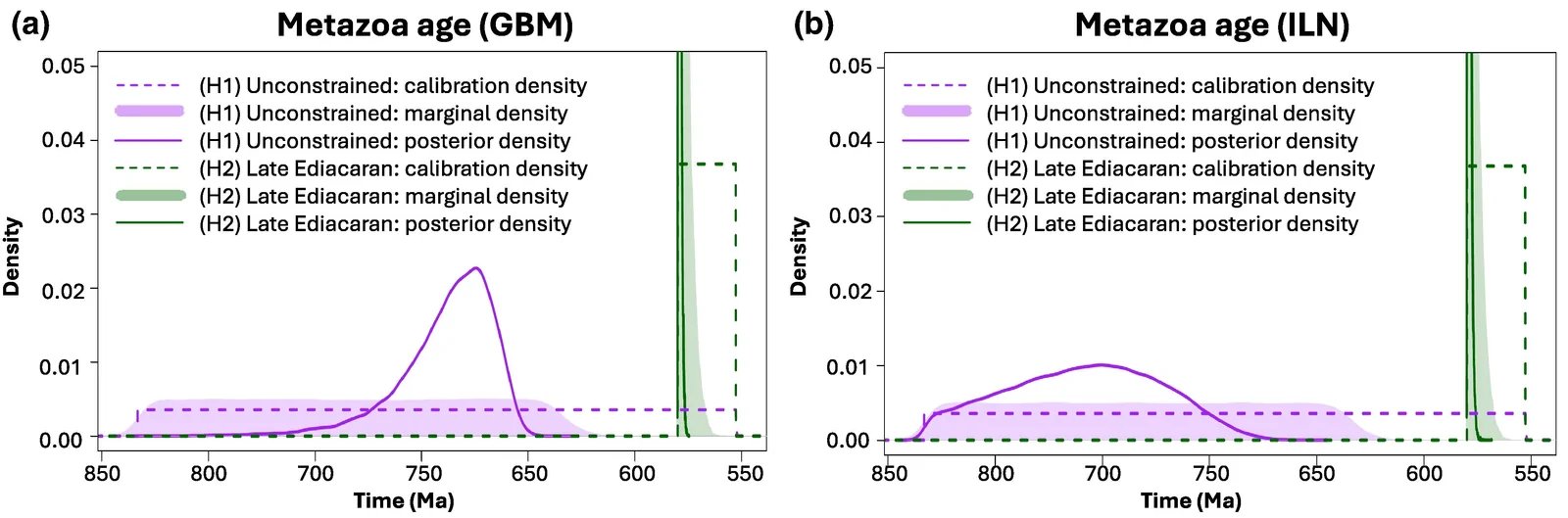
Fossil calibration and posterior densities on the age of Metazoa for dos Reis et al. (2015) original analysis (purple lines) and using Budd and Mann's (2024) revised calibration (green lines). Left (a): analyses under the GBM rate model. Right: analyses under the ILN rate model. Note that, when using Budd and Mann's calibration, the posterior density on the age of Metazoa is squashed against the maximum bound. This indicates a conflict between the molecular data and the fossil calibration.

Budd and Mann (2024) have argued that Metazoa (animals) cannot be older than its oldest Ediacaran fossil representatives (ca. 564.8 Ma, as of Μatthews et al. 2020) and criticize the Bayesian molecular-clock study of dos Reis et al. (2015) who obtained an older age for crown Metazoa (834 to 650 Ma). Budd and Mann (2024) reanalyzed the data of dos Reis et al. (2015) by placing a maximum bound of 580 Ma on the prior age of crown Metazoa. This bound is younger than the maximum bound of 833 Ma used by dos Reis et al. (2015) in the original study. Under the new younger maximum bound, the posterior age of Metazoa is younger than 580 Ma. Thus, Budd and Mann argue that molecular clocks are uninformative about the age of crown Metazoa because the posterior estimate can be moved from a maximum of 834 to 580 Ma by simply changing the maximum bound on the Metazoa age (Fig. 4).

However, the fact that the posterior of a parameter changes when the prior boundaries of the parameter change does not mean that the data are uninformative. Changing boundaries for a parameter is a dramatic change to the prior, and it is not surprising that the prior will have impacts on the posterior. In the Bayesian framework, one can calculate the marginal likelihood of a model (the likelihood averaged over the prior) or the posterior model probability to compare competing models. The marginal likelihood reflects not only the fit of the evolutionary model to the sequence data but also the compatibility of the prior and the data.

We repeated the analysis of Metazoa from Budd and Mann (2024) to calculate the marginal likelihood of two models (or hypotheses) of metazoan origination. Both hypotheses use the same minimum calibration of 552.85 Ma on the age of Metazoa, but they differ in the maximum bound used:

**H1 (unconstrained):** The age of crown Metazoa is allowed to be anywhere between 833 and 552.85 Ma; thus, we call H1 the unconstrained model. This is the original model used in dos Reis et al. (2015).

**H2 (late Ediacaran):** The age of crown Metazoa is restricted to the late Ediacaran between 580 and 552.85 Ma. This is Budd and Mann's modified model (2024), which assumes the age of crown Metazoa is close to its oldest Ediacaran fossil representatives.

For both models, we set the probability of violation of the maximum bound to zero (ie a hard bound) and the probability of minimum bound violation to 0.1% (ie a soft bound). The data and the rest of the fossil calibrations in the phylogeny are as described in dos Reis et al. (2015). Each model is analyzed twice, under the geometric Brownian motion (GBM) and independent log-normal (ILN) rate models (Rannala and Yang 2007). In the GBM model, rates for descendant branches have a log-normal distribution conditioned on the rate of the parent branch, thus generating autocorrelation among the branch rates. On the other hand, in the ILN model, rates for each branch are independent draws from a log-normal distribution, and thus there is no autocorrelation. Marginal likelihoods are calculated using the stepping-stones approach (Xie et al. 2011) as implemented in mcmc3r and MCMCtree (Panchaksaram et al. 2025), using approximate likelihood calculations (dos Reis and Yang 2011). The workflow developed for this approach is illustrated in Figure S1 (see also Figures S13 to S23 and Table S2 in Material for all MCMC diagnostics).

Application of the model selection procedure indicates the late Ediacaran model has a posterior probability of ∼0 due to a marked drop in marginal likelihood, both under the GBM and ILN models (Table 1). The unconstrained model under GBM has the highest marginal likelihood (Table 1) and should be considered the preferred model given the data analyzed and configuration of fossil calibrations used. Thus, the data provide strong evidence against the hypothesis that molecular data are uninformative about the age of crown Metazoa. Instead, examination of the posterior distribution (Fig. 4) and the results of the model selection procedure (Table 1) demonstrate the late Ediacaran model is a poor model for the timing of origin of Metazoa.

**Table 1:** Bayesian selection of metazoan origination model.

Metazoa origination model	Rate model	log L ± S.E.	Pr(M|D) (95% CI)	Mean posterior time estimates and 95% CI (Myr)
**H1 (unconstrained):** 833 to 552.85 Ma dos Reis et al. 2015	**GBM**	**−674.72** ± **0.09**	**1.00** (**1.00, 1.00)**	**688.1** (**658.1, 744.9)**
	ILN	−697.70 ± 0.06	1.04e^−10^ (8.54e^−11^, 1.27e^−10^)	759.0 (694.7, 825.8)
H2 (late Ediacaran): 580 to 552.85 Ma Budd and Mann 2024	GBM	−720.24 ± 0.08	1.70e^−20^ (1.35e^−20^, 2.15e^−20^)	579.4 (578.0, 580.00)
	ILN	−764.14 ± 0.06	1.46e^−39^ (1.19e^−39^, 1.79e^−39^)	579.3 (577.6, 580.00)

Log L, log of marginal likelihood; S.E., standard error, calculated with the delta method; Pr(M|D), posterior model probability, calculated assuming equal prior model probabilities; CI, equal-tail Credibility Interval. The model with highest posterior probability is highlighted in bold.

## A Bayesian model test of diversification timings in mammals

Budd and Mann (2024) have argued that a Cretaceous origin of placental mammals is not possible as the placental fossil record postdates the K-Pg mass extinction. They reanalyzed the mammal data of Álvarez-Carretero et al. (2022) and showed that, by placing a maximum bound on the age of Placentalia at the K-Pg boundary (66.09 Ma), they could make the origin of Placentalia postdate the mass extinction. In their original analysis, Álvarez-Carretero et al. (2022) used a maximum bound of 162.5 Ma on the age of Placentalia, obtaining a mean posterior age of 80.284 Ma (83.32 to 77.62 Ma) (Fig. 3). Thus, Budd and Mann (2024) argued that molecular clocks are uninformative about the age of Placentalia because the estimate can be made to predate or postdate the K-Pg simply by changing the maximum bound used. Again, we use Bayesian model comparison to assess the competing hypotheses.

We test two models of Placental mammal origination:

**H1 (unconstrained):** The calibration on the age of Placentalia lies between 162.5 and 61.66 Ma with probability of violation of the bounds equal to 5% (2.5% each bound). Thus, this model is unconstrained as it is compatible with post- or pre-K-Pg originations of Placentalia. This is the original model used by Álvarez-Carretero et al. (2022).

**H2 (post-K-Pg):** The age of Placentalia is constrained to postdate the K-Pg between 66.09 and 61.66 Ma; this is the model espoused by Budd and Mann (2024). We set the probability of violation of the K-Pg maximum to 0, while the probability of violating the minimum constraint is kept to 2.5%.

The data are analyzed as for the Metazoa case, using mcmc3r and MCMCtree to calculate the marginal likelihood under the stepping-stones method using approximate likelihood (dos Reis and Yang 2011; Xie et al. 2011; Panchaksaram et al. 2025) (see Figure S1). The data and remaining fossil calibrations for the 72 species phylogeny are as in Álvarez-Carretero et al. (2022). MCMC diagnostics and illustrating comparisons of estimated divergence times and evolutionary rates are shown in Figures S2 to S12 and Table S1.

**Figure 5:**
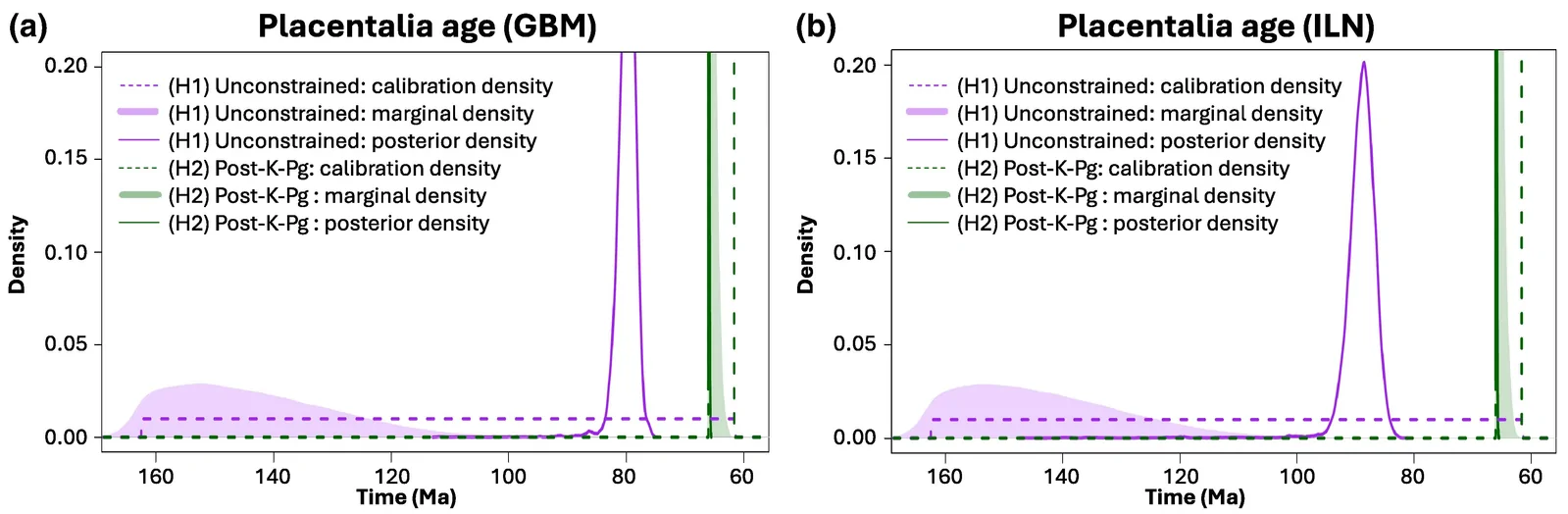
Fossil calibration and posterior densities on the age of Placentalia for Álvarez-Carretero et al. (2022) original analysis (purple lines) and using Budd and Mann (2024)'s revised calibration with a hard bound on the maximum age (green lines). Left (a): analyses under the GBM rate model. Right (b): analyses under the ILN rate model. Note that, when using Budd and Mann's calibration, the posterior density on the age of Placentalia is squashed against the maximum bound. This indicates a conflict between the molecular data and the fossil calibration.

Table 2 shows the results of the Bayesian model selection analysis. Budd and Mann's post-K-Pg origination model for Placentalia has a posterior probability of ∼0. Furthermore, the posterior distribution of Placentalia's age is very narrow and concentrated at the maximum bound at 66 Ma (Fig. 5), which indicates conflict between the molecular data and the K-Pg constraint used. Thus, we find no evidence for the claim that molecular clocks are uninformative about the age of Placentalia.

**Table 2:** Bayesian selection of placental mammal origination model.

Placental Mammals origination model	Rate model	log L ± S.E.	Pr(M|D) (95% CI)	Mean posterior time estimates and 95% CI (Myr)
**H1 (unconstrained):** 162.5 to 61.66 Ma Álvarez-Carretero et al. (2022)	**GBM**	**−2,509.80** ± **0.16**	**1.00** (**1.00, 1.00)**	**80.0** (**77.4, 83.2)**
	ILN	−2,612.74 ± 0.12	1.96e^−45^ (1.33e^−45^, 2.93e^−45^)	89.2 (85.1, 94.0)
H2 (post-K-Pg): 66.09 to 61.66 Ma Budd and Mann (2024)	GBM	−2,609.06 ± 1.56	7.80e^−44^ (4.04e^−45^, 1.61e^−42^)	66.0 (65.9, 66.1)
	ILN	−2,774.48 ± 1.92	1.12e^−115^ (2.81e^−117^, 4.86e^−114^)	66.0 (65.9, 66.1)

Log L, log of marginal likelihood; S.E., standard error, calculated with the delta method; Pr(M|D), posterior model probability, calculated assuming equal model prior probabilities; CI, equal-tail Credibility Interval. The model with highest posterior probability is highlighted in bold.

## Truncation, calibration densities, and effective priors

Yang and Rannala (2006) highlighted the difference between the user-specified calibration densities and the joint prior distribution for node ages used by the molecular-clock dating algorithm, known also as the *effective* prior. Because the age of an ancestral node must be older than the age of a descendent node, the joint prior density cannot simply be obtained by multiplying the calibration densities specified for individual nodes. Instead, it is the product of the calibration densities conditional on the intrinsic constraints imposed by the tree topology. When the prior bounds of ancestral and descendant nodes overlap substantially, the discrepancy between the naïve product of marginal calibration densities and the joint prior density can be considerable (Figure S24).

Ideally, calibration information should be specified in the form of joint prior distributions for the ages of multiple calibration nodes. Such joint densities may be generated from probabilistic modeling and statistical analysis of occurrences of fossils in the rock strata or of morphological characters for fossil taxa (Wilkinson et al. 2011; Ronquist et al. 2012; Heath et al. 2014; Zhang et al. 2016). Without a formal statistical analysis, it may be challenging to specify a joint prior distribution, even for two calibration nodes. Interpretations of fossils are complex, and, furthermore, joint probability distributions are in general hard to grasp. A pragmatic approach commonly adopted in dating analyses is for the researcher to specify calibration densities for individual nodes based on fossil evidence, and the dating program enforces the intrinsic constraint through truncation. This procedure modifies the calibration densities to produce a new joint distribution for node ages that satisfies the intrinsic constraint. When multiple calibration nodes interact through the truncation, the resulting effective prior, or the implied marginal distributions of node ages, may differ substantially from the calibration densities originally specified by the user. It is essential for the researcher to assess the effective prior to ensure that it is an adequate summary of the fossil evidence. In practice, this can be done by running the MCMC program without using the molecular sequence data and inspecting the resulting distribution of node ages.

## Unsurprising correlations and the role of fossils in molecular-clock dating

**Figure 6:**
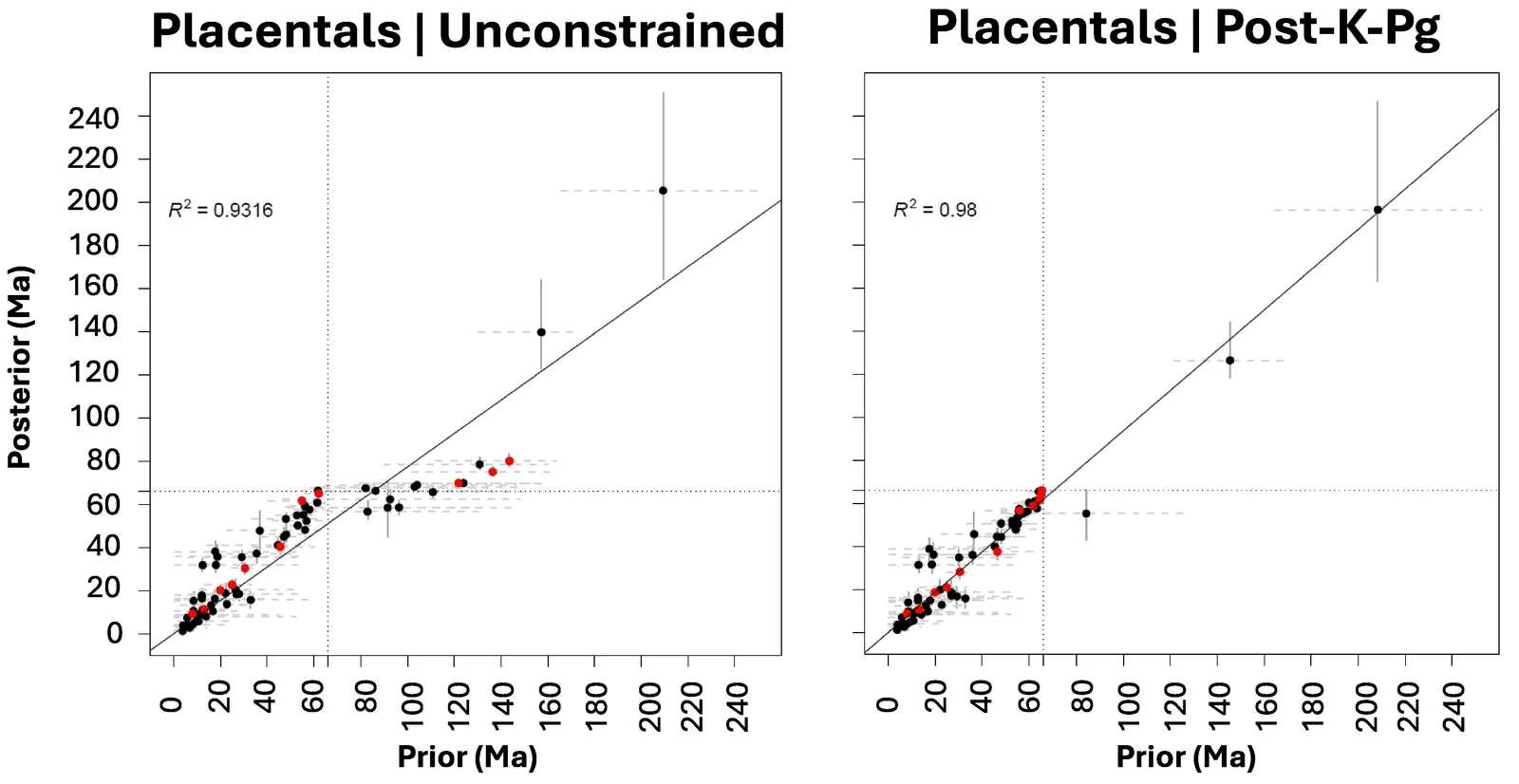
Posterior ages vs. prior ages for placental mammals. Prior vs. posterior means for unconstrained (left) and post-K-Pg (right) models of placental mammal diversification. Nodes are plotted at their prior/posterior means, and the horizontal (prior) and vertical (posterior) bars are the corresponding 95% HPD intervals. Red dots show the posterior means for node ages along the last common ancestor of placental to human lineage (see text). The prior and posterior means of node ages are highly correlated, but the posterior estimates tend to have narrower CIs. The data are analyzed under the GBM rate model in all cases.

In analyses of two datasets involving animals and placental mammals, Budd and Mann (2024) observed strong correlations between posterior means of node ages with the prior means and concluded that molecular clocks are largely uninformative because the posterior appears to recapitulate the prior. We note two things. Firstly, this correlation is unsurprising because it arises due to the ordered nature of nodes in phylogenies. For example, consider the lineage linking the Human–Chimp ancestor to the Placental ancestor: this lineage contains 11 node ages, ordered from the youngest (Human–Chimp) to the oldest (Placentalia, eg Human–Elephant). Thus, the correlation arises as a natural consequence of the prior and posterior respecting the ordering of node ages (Fig. 6), and thus it is neither abnormal nor pathological. Secondly, Budd and Mann (2024)’s interpretation that the correlation evidences an uninformative clock is incorrect and reflects a misunderstanding of how branch lengths from molecular sequences and fossil calibrations interact in divergence–time estimation. Molecular sequence data provide information about evolutionary distances or branch lengths, measured in the expected number of substitutions per site, which are the products of evolutionary rates and divergence times (Thorne et al. 1998; dos Reis and Yang 2013). Molecular data alone cannot estimate substitution rates and divergence times separately. A short time period with a high substitution rate may produce sequence data indistinguishable from those generated by a long time period and a slow rate. Fossil calibrations with absolute geological times provide information to resolve this confounding effect between time and rate.

Through branch lengths, molecular data provide at least two kinds of information. Firstly, molecular data contribute information about the relative ages of nodes. For example, molecular branch lengths may indicate that one species divergence event is twice as old as another. Even if the posterior for a calibration node age is similar to the prior, the joint posterior for multiple nodes is typically very different from the joint prior. Secondly, molecular data also propagate temporal information throughout the phylogeny. Fossil calibrations typically constrain only a small subset of nodes, but the molecular sequence data help distribute that information to all nodes on the phylogeny by enforcing consistency with the estimated branch lengths. Large gaps in time between consecutive nodes are often associated with long molecular branches, whereas short temporal intervals correspond to short branches. Simulation studies illustrate the power of molecular data in shaping divergence–time estimates. For example, Yang and Rannala (2006) demonstrate that even when fossil calibrations are grossly incorrect, molecular sequence data may still lead to accurate age estimates because the relative branch lengths impose strong constraints on the temporal structure of the tree. In such cases, the molecular data effectively “push” or “pull” node ages until they become consistent with the relative branch lengths (eg Fig. 6 in Yang and Rannala 2006).

**Figure 7:**
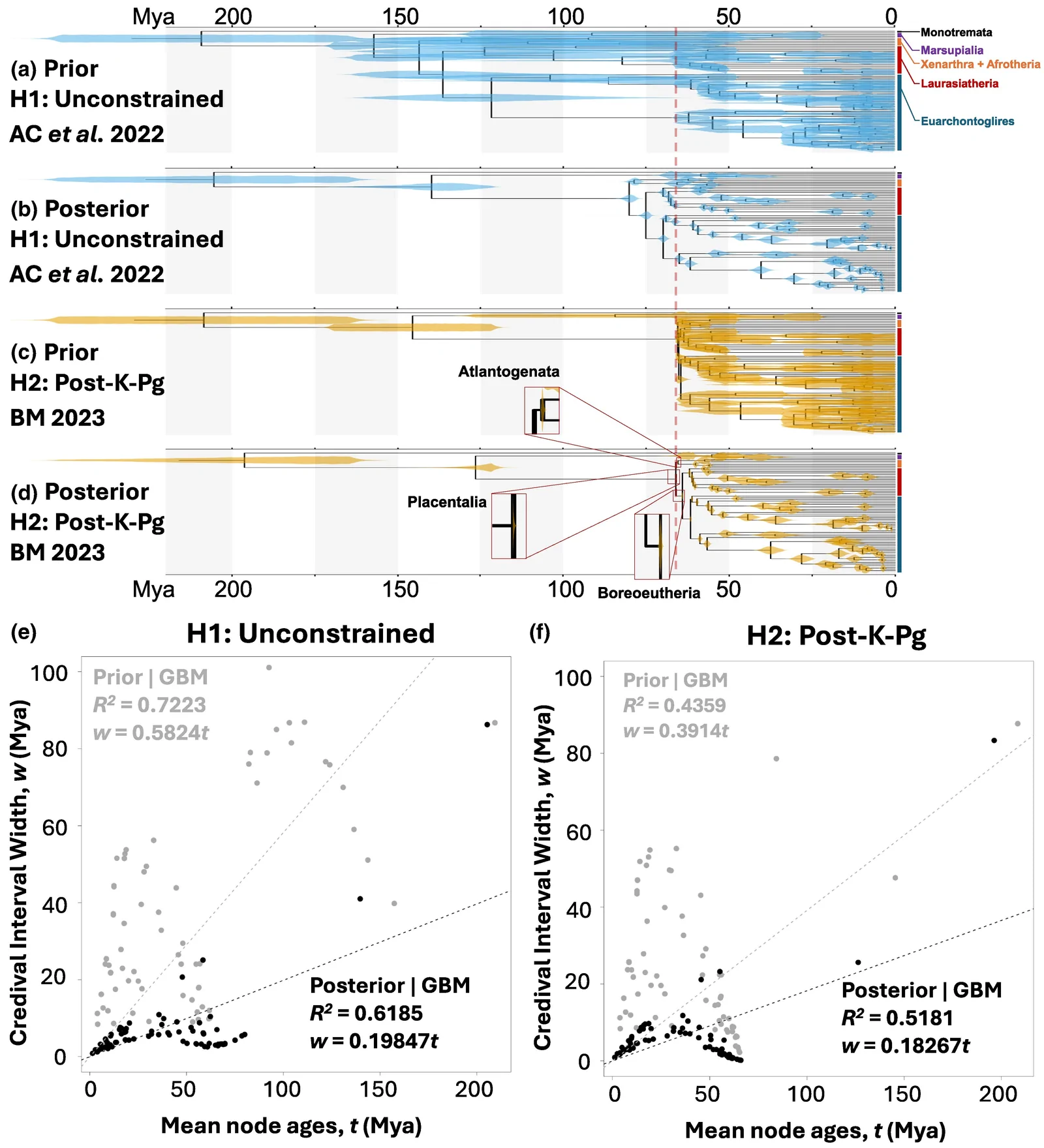
Posterior and prior ages for placental mammals analyzed under two diversification models. The GBM rate model is used. (a) Prior and (b) posterior timetrees for the unconstrained model of placental mammal diversification. (c) Prior and (d) posterior timetrees for the post-K-Pg model of placental diversification. Note posterior time densities for nodes Placentalia, Atlantogenata, and Boreoeutheria have had to be zoomed in as the posterior densities are squished against their hard maximum constraints. The vertical dashed line is the K-Pg boundary at 66 Ma. Panels (e) and (f) are infinite-sites plots for the unconstrained (e) and the post-K-Pg (f) models. Evolutionary timelines (a–d) were generated with TreeViewer (Bianchini and Sánchez-Baracaldo 2024).

Finally, it is inaccurate to claim that the posterior recapitulates the prior in either the animal or the placental mammal dataset. Indeed, the prior and posterior mean divergence times show striking differences (Fig. 7a to d, for the placental mammal dataset). Under the unconstrained model (H1), the prior is diffuse and the 95% HPD intervals for many nodes span the K-Pg boundary. In contrast, the posterior is far more concentrated, with predominantly post-K-Pg diversification of placental mammal orders with a conspicuous pre-K-Pg ancestor for all placentals.

The amount of information from the molecular data relative to that from the prior (or fossil calibrations), as well as the limits of divergence–time estimates when the amount of molecular data approaches infinity, can be formally quantified using the infinite-sites and finite-sites theories developed by Yang and Rannala (2006), Rannala and Yang (2007), dos Reis and Yang (2013), and Zhu et al. (2015). For the mammal case, the infinite-sites plot evidences a marked reduction in uncertainty in the posterior of times when compared to the prior (Fig. 7e, f).

## On the nature of molecular rates

Models of rate variation are required to obtain posterior node ages, and thus assumptions about these models can have an impact (eg Lartillot and Poujol 2011; Berv and Field 2018). However, models of rate variation must be grounded on empirical and theoretical understanding of the evolution of molecular rates. Characteristics of the DNA–damage–response–repair mechanism and life–history traits, such as genome size and effective population size, impose a limit on the mutation rate of organisms (Lynch 2010; Lynch et al. 2016). As pointed out by Springer et al. (2013), forcing divergence timings to equate the age of oldest fossils can result in estimated molecular rates that appear physiologically implausible. There can be no doubt that there remains room for the development of molecular-clock methods and, indeed, there are now diverse families of approaches for divergence–time estimation that allow researchers to explore the impact of different biological phenomena that impact on the rate and timing of evolution (Donoghue and Yang 2016). We welcome the development of different approaches to integrating fossil evidence (Heath et al. 2014), violent shifts in rate variation (Landis et al. 2013), and phenotypic traits that might be correlated with and informative of rate variation (Lartillot and Delsuc 2012). These and other developments allow researchers to test hypotheses on the nature of major diversification events, freeing them from simply interpreting evidence within the constraints of competing hypotheses, such as those that attend the diversification of bilaterian animals and placental mammals. Nevertheless, simulation-based studies suggest that existing molecular-clock methods can provide accuracy in the estimation of evolutionary timescales (eg Zhang et al. 2016; Warnock et al. 2017).

## A critique of the literalist interpretation of the fossil record

The fossil record remains an important and unparalleled archive of evolutionary history, offering insights into organisms and their ecologies over time. However, it is inherently incomplete and requires careful interpretation. We have argued that efforts to impose a more or less literal reading of the fossil record onto evolutionary history risk yielding evolutionary timescales for animals and mammals that are statistically improbable, inconsistent with molecular data, and unsupported by model-based inference. Framing molecular clocks and the fossil record as alternative or competing approaches to estimating evolutionary timescales is therefore misleading, since molecular-clock analyses explicitly incorporate and are conditioned upon fossil evidence. Many phylogenetic and non-phylogenetic models are available for analyzing the fossil record, whether in combination with molecular data or independently. Although reconciling the results of different methods and the timescales they produce can be challenging, these approaches nonetheless provide a more robust foundation than timelines derived from a more or less literal interpretation of the fossil record alone.

When we reject a literalist interpretation of the fossil record for animals and placental mammals, we do not suggest the record is generally unreliable. Instead, it is clear that, in some instances, the fossil record provides a good approximation of the antiquity of clades, while in other cases it provides a poor approximation of clade age. This is exemplified by placental mammals: the latest Bayesian analyses provide diversification timings for ordinal-level crown groups that are close to their fossil records, but not for the common ancestor of the group. Determining whether or not fossils are good approximations of crown–clade divergence times requires the weighting of all available evidence. Here, we have proposed an approach to do just so: Bayesian model selection lets us compare a priori-specified hypotheses of diversification times that can be assessed by the weight of fossil and molecular evidence.

## Acknowledgments

We thank Graham E Budd and Richard P Mann for sharing the files needed to reproduce their analyses and for helpful discussions at the “Rocks And Clocks” meeting (Montréal, 2025), where SÁ-C presented the Bayesian model selection results.

## Supplementary Material

msag211_Supplementary_Data

## Data Availability

The code, data, and step-by-step guidelines to reproduce our analyses are available at https://github.com/sabifo4/BayesModSel. Large output MCMC files are available at https://doi.org/10.6084/m9.figshare.32033958. The original alignments for the animal and mammal datasets are available at https://doi.org/10.6084/m9.figshare.1525089 and https://doi.org/10.6084/m9.figshare.14885691, respectively.
