# Supplementary material for: On the origin of animals and placental mammals: a critique of literalist readings of the fossil record
Source: Mol Biol Evol. 2026 Aug 19;43(9):msag211. doi: 10.1093/molbev/msag211 (PMC13543917; doi:10.1093/molbev/msag211)
Supplement: Supplementary file 1 [file msag211_supplementary_data.zip › ACetal26_firstlook_msMBE-260406_SuppMat.pdf]

Supplementary Material for *"On the Origin of Animals and Placental Mammals: A Critique of Literalist Readings of the Fossil Record"*

Sandra Álvarez-Carretero[1,✉], Ziheng Yang[1], Philip C. J. Donoghue[2,✉], and Mario dos Reis[3,✉]

[1]Department of Genetics, Evolution and Environment, University College London, London, UK
[2]Bristol Palaeobiology Group, School of Earth Sciences, University of Bristol, BS8 1TQ, Bristol, UK
[3]School of Biological and Behavioural Sciences, Queen Mary University of London, London, UK

✉ E-mails: s.alvarez-carretero@ucl.ac.uk, phil.donoghue@bristol.ac.uk, m.dosreisbarros@qmul.ac.uk.

Bayesian model selection: an overview

In a Bayesian model selection analysis, we need to infer the marginal likelihood values of the competing models. In this study, we use the steppingstone sampling (SS) approach (Xie *et al.* 2011) for this estimation, which uses K β points to calculate the corresponding power posteriors,

$$f(D|\theta)^{\beta} = \frac{f(\theta)f(D|\theta)^{\beta}}{c_{\beta}} \text{ (Eq. S1),}$$

where D is the dataset, $f_{\beta}(\theta|D)$ the target distribution, $f(\theta)$ the prior, $f(D|\theta)^{\beta}$ the likelihood, and c_{β} the normalising constant. Following Eq. S1, when $\beta = 0$, the likelihood term is equal to 1. To this end, the target distribution is equal to the prior distribution. If $\beta = 1$, the target distribution is the posterior distribution. Nevertheless, when $0 < \beta < 1$, the target distribution becomes a power posterior distribution that will change according to the value of the β point to which the likelihood term is exponentiated. The marginal likelihood can therefore be regarded as the area under the path that connects the prior distribution, $f_{\beta=0}(\theta|D) \simeq f(\theta)$, to the posterior distribution, $f_{\beta=1}(\theta|D) \simeq f(\theta)f(D|\theta)^{\beta=1}$, through multiple power posteriors, $f_{0<\beta<1}(\theta|D)$.

When the likelihood is raised to values of β close to 0, however, the likelihood lies within a very unstable area of the parameter space, quickly changing between the prior and the posterior. To address this issue, Xie *et al.* (2011) investigated the impact of drawing β values from a Beta(α, 1.0) distribution when setting different values of α. Specifically, drawing β values from a Beta distribution with $\alpha = 0.3$ seems to be the most optimal approach; half of the drawn values were less than 0.1 (Xie *et al.* 2011).

To evaluate M_i contested models, we can compute the ratio between the two largest estimated marginal likelihood values (e.g., $p(D|M_1)$ and $p(D|M_2)$), which returns the corresponding Bayes factor (BF, Jeffreys 1935):

$$BF_{12} = \frac{p(D|M_1)}{p(D|M_2)} \text{ (Eq. S2).}$$

If log marginal likelihood values are used instead, then we would calculate the difference between the two largest estimated log marginal likelihood values and then compute the exponential:

$$log\ BF_{12} = \ exp(log\ p(D|M_1) - log\ p(D|M_2) \text{ (Eq. S3).}$$

For instance, in our test case 1 (origin of placental mammals), we evaluated the fitness of two contested diversification models (i.e., "H1: Unconstrained" and "H2: Post-K-Pg") under two relaxed-clock models (i.e., autocorrelated-rates [or geometric Brownian motion, GBM] model and independent-rates model [ILN]); we evaluated a total of 4 models (i.e., "Unconstrained | GBM", "Post-K-Pg | GBM", "Unconstrained | ILN", "Post-K-Pg | ILN"). After estimating the log marginal likelihood values of each model, we used the two largest values to calculate the log BF following Eq. S3. The same would apply when assessing the fitness of the diversification models tested in our test case 2 (origin of animals). Bayes factors are widely used to evaluate how fitting a model is for the dataset being analysed.

If the prior on a given model is not equally distributed, however, it is also relevant to compute posterior model probabilities alongside BFs. For illustrative purposes, Eq. S4 shows how to compute such a probability for a given model, M_1, although the same procedure applies to other models being evaluated:

$$p(M_1|D) = \frac{p(D|M_1)p(D|M_1)}{\sum_{i=1}^{N} \ \ p(D|M_i)p(D|M_i)} \text{ (Eq. S4).}$$

To this end, we can update the BF calculation as a ratio of posterior model probabilities in which the prior weight on the models, $p(M_i)$, is accounted for as prior odds:

$$\frac{p(M_1|D)}{p(M_2|D)} = \frac{p(D|M_1)}{p(D|M_2)} \times \frac{p(M_1)}{p(M_2)} = BF_{12} \times \frac{p(M_1)}{p(M_2)} \text{ (Eq. S5).}$$

As shown in Eqs. S4 and S5, the choice of priors can substantially affect the calculation of BFs, and thus caution must be taken when specifying such distributions.

To calculate the BFs and posterior model probabilities, we used the `mcmc3r` R package as detailed in the section below and in our GitHub repository "BayesModsel" (see Data Availability section for details).

Running a Bayesian model selection analysis with `mcmc3r` and `MCMCtree`

To run our Bayesian model selection analyses, we used the `mcmc3r` R package (dos Reis *et al.* 2018) and the dating program `MCMCtree`, part of the PAML software (Yang 2007). Below, you can find the various steps that we followed to (i) prepare the file structure with `mcmc3r`, (ii) relocate input data and control files using bash scripting, (iii) run `MCMCtree` specifying to collect likelihood samples from power posteriors, and (iv) estimate log marginal likelihood values for every competing model and compute BFs and posterior model probabilities using `mcmc3r`.

To prepare the file structure, we ran our in-house R script `Generate_BFS_inp.R` (we wrote two scripts, one for each dataset). Please note that, while this script is customised to work with the data analysed in this study, one can easily adapt the global variables to work with their own datasets.

Once the global variables are defined in the aforementioned R script, the next block code runs the `mcmc3r::make.beta` R function, which generates K β points under one of the two implemented approaches: thermodynamic integration (Lartillot and Philippe 2006) or steppingstone sampling approach (Xie *et al.* 2011). In this study, we used the latter approach, which uses Eq. S6 to generate K β points:

$$\beta_i = \left(\frac{i-1}{K}\right)^a \quad \text{(Eq. S6)}.$$

Specifically, K is the total number of β points to be generated, β_i is the i-th β point being generated (i.e., $1 < i < K$), and a is the value that controls how close to zero the β points are to be generated. We used a value of $a = 5$ given that ~60% of the β points were lower than 0.1, which helped us sample the unstable area of the parameter space close to the prior distribution. The resulting β points are saved in a text file that we decided to call `betaweights.txt` (this file name can be modified). In our test case 1 (origin of placental mammals), we used $K = 256$ β points to estimate the log marginal likelihood values under each model, while we used $K = 128$ β points for our test case 2 (origin of animals). The area in the parameter space close to the prior was more unstable when analysing our first dataset, and so we decided to double the number of β points with the aim to decrease the standard error of the estimated marginal likelihood values.

Subsequently, a directory for each model is created (i.e., M*X* directories). Inside these M*X* directories, the `mcmc3r::make.bfctlf` R function is run to first create K subdirectories (i.e., as many subdirectories as power posteriors `MCMCtree` will collect likelihood samples from). Then, within each of these subdirectories, the function creates the control file that will be used to run `MCMCtree`. At this stage, each control file will have only one line setting variable `betaBayesFactorBeta` to the corresponding β point. For instance, directory 1 will have a control file with a line reading `betaBayesFactorBeta = 1e-300`; the target distribution (power posterior) will be the prior. The rest of directories labelled from 2 to K will have a control file where the rest of β points previously generated by the `mcmc3r::make.beta` R function are specified (i.e., $0 < \beta < 1$). By enabling variable `betaBayesFactorBeta`, `MCMCtree` samples from the power posterior calculated with the β point written in the control file.

The last code block in the R script will (i) read the lines of a template control file with the rest of variables required to run `MCMCtree` and (ii) append them to the previously generated control files with only variable `betaBayesFactorBeta`. Please note that all variables in the template control file are pre-defined by the user according to the dataset being analysed. Nevertheless, there are two unspecified variables: `clock` and `treefile`. The flags given to these two variables will be replaced with the relevant options depending on the subdirectory where the control file is.

The previously described steps are illustrated in Fig. S1 (**steps 1.1 and 1.2)**. The following steps (Fig. S1, **step 2**) involve allocating relevant input files (sequence alignment, calibrated tree topology, and, if required, the in.BV file with the estimated branch lengths, the Hessian, and the gradient required to approximate the likelihood calculation) to subdirectories 1 to K within each M*X* directory. There are multiple approaches to carry out this step; we chose bash scripting as shown in the first code snippet in section *"2. Run MCMCtree"* in the README files available in our GitHub repository "BayesModsel" (see Data Availability section for details; see "00_mammals/01_modsel_workflow/README.md" and "01_animals/01_modsel_workflow/README.md"). Please note that the alignment and the `betaweights.txt` files are always the same; only the tree file and control file will change for each model (i.e., different tree hypothesis and different tree file names).

Once all directories have the relevant input and control files, `MCMCtree` will target a power posterior distribution defined by the β point given by variable `betaBayesFactorBeta` in each control file. For each power posterior, a total of n likelihood samples, $f(D|MX,\vartheta)^{\beta_k}$, will be collected; $1 < k < K$. When analysing our datasets, we set variable `nsample` to 20,000 in all control files, and thus we collected a total of $n = 20{,}000$ likelihood samples when targeting each power posterior (these settings can be updated in the template control file). The likelihood samples are used to estimate the log marginal likelihood values of each model alongside the corresponding β points.

The log marginal likelihood values, $log\ \hat{r}_{SS}$, are calculated using the `mcmc3r::stepping.stones` R function, which adapts Eqs. S7 and S8 from Xie *et al.* (2011):

$$\hat{r}_{SS,k} = \frac{1}{n}\sum_{i=1}^{n} f(D|MX,\vartheta_{k-1,i})^{\beta_k-\beta_{k-1}} \text{ (Eq. S7)},$$

$$log\ \hat{r}_{SS} = \sum_{k=1}^{K} log(\hat{r}_{SS,k}) \text{ (Eq. S8)}.$$

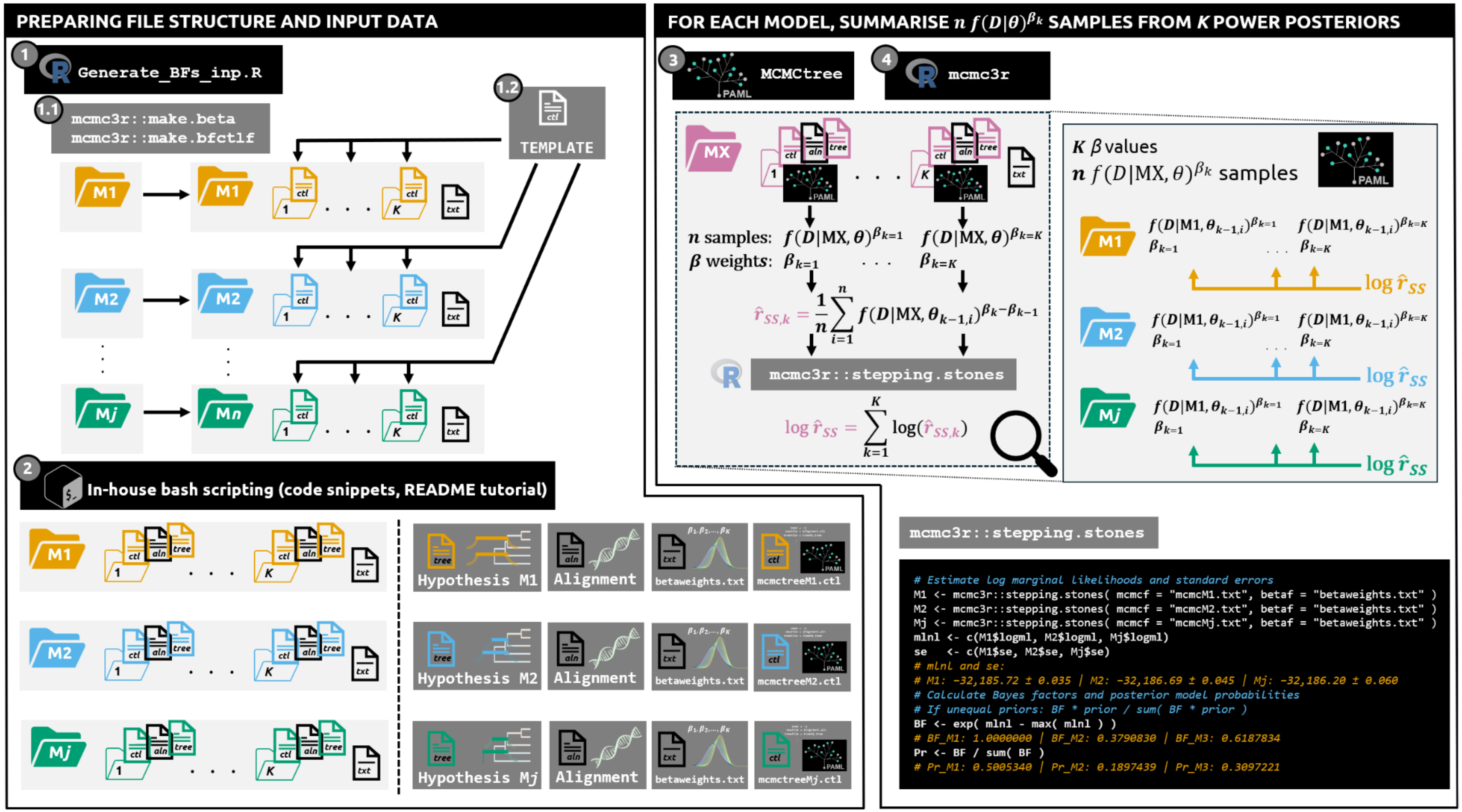

PREPARING FILE STRUCTURE AND INPUT DATA
1 Generate_BFs_inp.R
1.1 mcmc3r::make.beta
mcmc3r::make.bfctlf
1.2 TEMPLATE
M1
M2
Mj
Mn
2 In-house bash scripting (code snippets, README tutorial)
Hypothesis M1
Hypothesis M2
Hypothesis Mj
Alignment
betaweights.txt
mcmctreeM1.ctl
mcmctreeM2.ctl
mcmctreeMj.ctl
FOR EACH MODEL, SUMMARISE $n\ f(D|\theta)^{\beta_k}$ SAMPLES FROM K POWER POSTERIORS
3 MCMCtree
4 mcmc3r
MX
n samples: $f(D|\mathrm{MX},\theta)^{\beta_{k=1}}$ . . . $f(D|\mathrm{MX},\theta)^{\beta_{k=K}}$
β weights: $\beta_{k=1}$ . . . $\beta_{k=K}$
$\hat{r}_{SS,k} = \frac{1}{n}\sum_{i=1}^{n} f(D|\mathrm{MX},\theta_{k-1,i})^{\beta_k-\beta_{k-1}}$
mcmc3r::stepping.stones
$\log \hat{r}_{SS} = \sum_{k=1}^{K} \log(\hat{r}_{SS,k})$
K β values
$n\ f(D|\mathrm{MX},\theta)^{\beta_k}$ samples
$f(D|\mathrm{M1},\theta_{k-1,i})^{\beta_{k=1}}$ $\beta_{k=1}$ . . . $f(D|\mathrm{M1},\theta_{k-1,i})^{\beta_{k=K}}$ $\beta_{k=K}$
$\log \hat{r}_{SS}$
mcmc3r::stepping.stones
# Estimate log marginal likelihoods and standard errors
M1 <- mcmc3r::stepping.stones(mcmcf = "mcmcM1.txt", betaf = "betaweights.txt")
M2 <- mcmc3r::stepping.stones(mcmcf = "mcmcM2.txt", betaf = "betaweights.txt")
Mj <- mcmc3r::stepping.stones(mcmcf = "mcmcMj.txt", betaf = "betaweights.txt")
mlnl <- c(M1$logml, M2$logml, Mj$logml)
se <- c(M1$se, M2$se, Mj$se)
# mlnL and se:
# M1: -32,185.72 ± 0.035 | M2: -32,186.69 ± 0.045 | Mj: -32,186.20 ± 0.060
# Calculate Bayes factors and posterior model probabilities
# If unequal priors: BF * prior / sum(BF * prior)
BF <- exp(mlnl - max(mlnl))
# BF_M1: 1.0000000 | BF_M2: 0.3790830 | BF_M3: 0.6187834
Pr <- BF / sum(BF)
# Pr_M1: 0.5005340 | Pr_M2: 0.1897439 | Pr_M3: 0.3097221

Figure S1. Bayesian model selection analysis workflow using `MCMCtree` and `mcmc3r`. The R script `Generate_BFs_inp.R` is used for preparing the file structure and relocating input data and control files. Step 1.1 illustrates the outcome of running the `mcmc3r::make.beta` and `mcmc3r::make.bfctlf` R functions. One directory for each model being evaluated will be created with (i) as many subdirectories as power posteriors are to be run with an `MCMCtree` control file labelled from 1 to K and (ii) a text file (betaweights.txt) with K β points. The control files will have only one line with variable `betaBayesFactorBeta` specifying the β point that will determine the power posterior from which the likelihood samples will be collected when running `MCMCtree`. In step 1.2, the lines of a template control file are read and appended to each of the previously generated control files. The options for clock and treefile variables are updated according to the MX directory where the control files are. Input files (sequence alignment and calibrated tree topology) are then included in each subdirectory using bash scripting. If the approximate likelihood calculation is to be enabled when running `MCMCtree`, the "`in.BV`" file (i.e., estimated branch lengths, gradient, and Hessian) should also be added. Subsequently, `MCMCtree` will be run for each model within each subdirectory (step 3). The n likelihood samples collected when sampling from each $k$power posterior under each model will be used, alongside the corresponding β points, to estimate the log marginal likelihood values, standard errors, Bayes factors, and posterior model probabilities with `mcmc3r` (step 4).

MCMC diagnostics when running MCMCtree with the mammal dataset

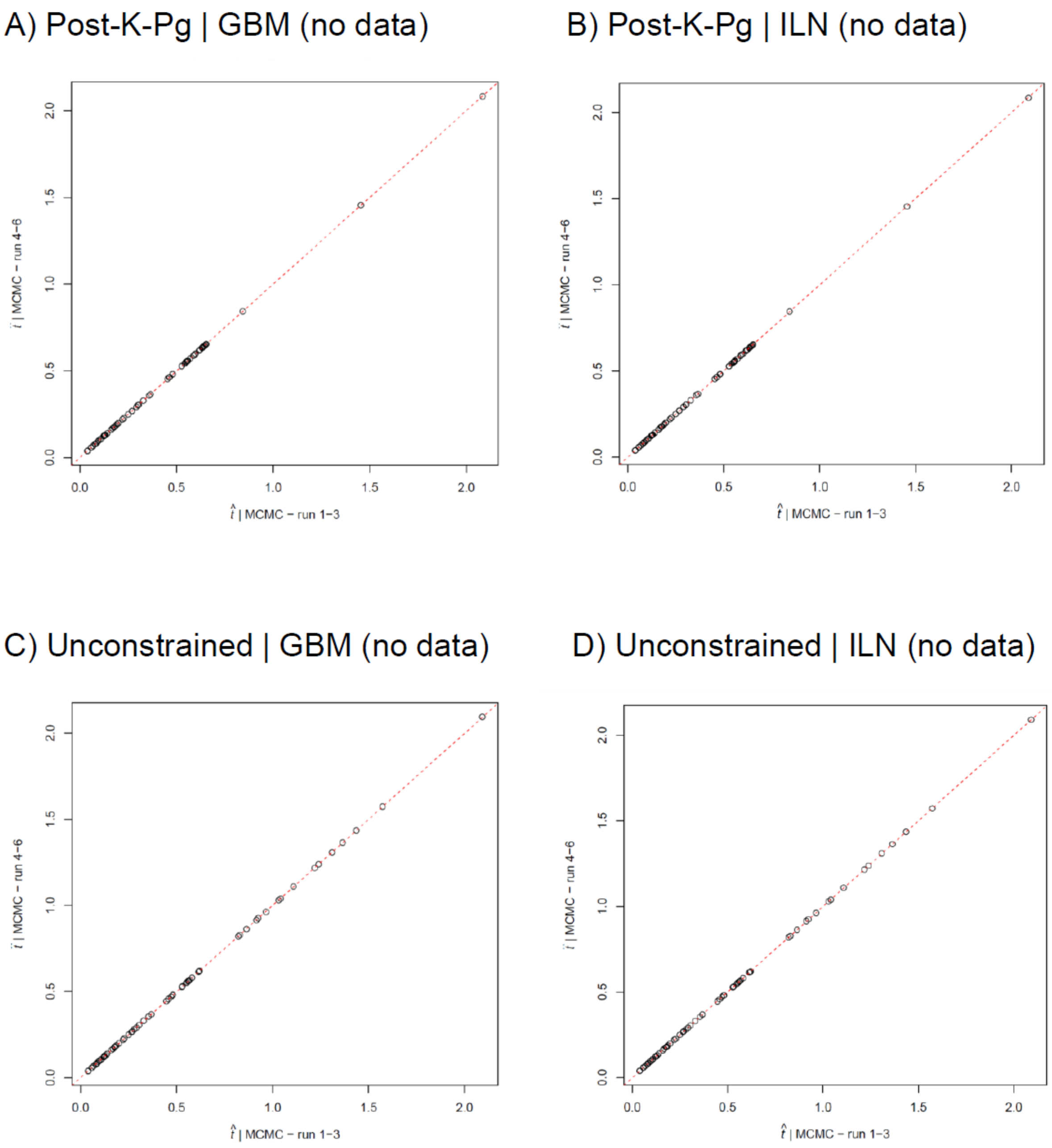

Figure S2. Convergence plots for the summarised divergence times estimated under each of the diversification models testing the origin of placental mammals when data were not used. The mean divergence times were calculated using the chains that passed the filters during the MCMC diagnostics that were run for each diversification model and under both the geometric Brownian motion (GBM, autocorrelated rates) and the independent-rates log-normal (ILN) relaxed-clock models when data were not included (i.e., the target distribution from which sampling took place was the prior distribution). The first row has the convergence plots for the Post-K-Pg model (A: GBM, B: ILN) and the last row for the Unconstrained model (C: GBM, D: ILN). The samples collected for half of the chains that passed the filters were summarised and the divergence times plotted on the x axis. The same was done for the other half; the divergence times were plotted on the y axis.

All convergence plots show that that x ≃ y, and thus this qualitative check for chain convergence was passed for all analyses. Time unit is set to 100 Myr. The in-house R script used to obtain these plots generates one individual PDF file for each model. The script and output files are available in the GitHub repository. Path for script: “BayesModSel/00_mammals/02_rate_per_branch/01_MCMC_diagnostics/scripts/MCMC_diagnostics_prior.R”. Path for output files: “BayesModSel/00_mammals/02_rate_per_branch/01_MCMC_diagnostics/sum_files_prior/MCMC_diagnostics”.

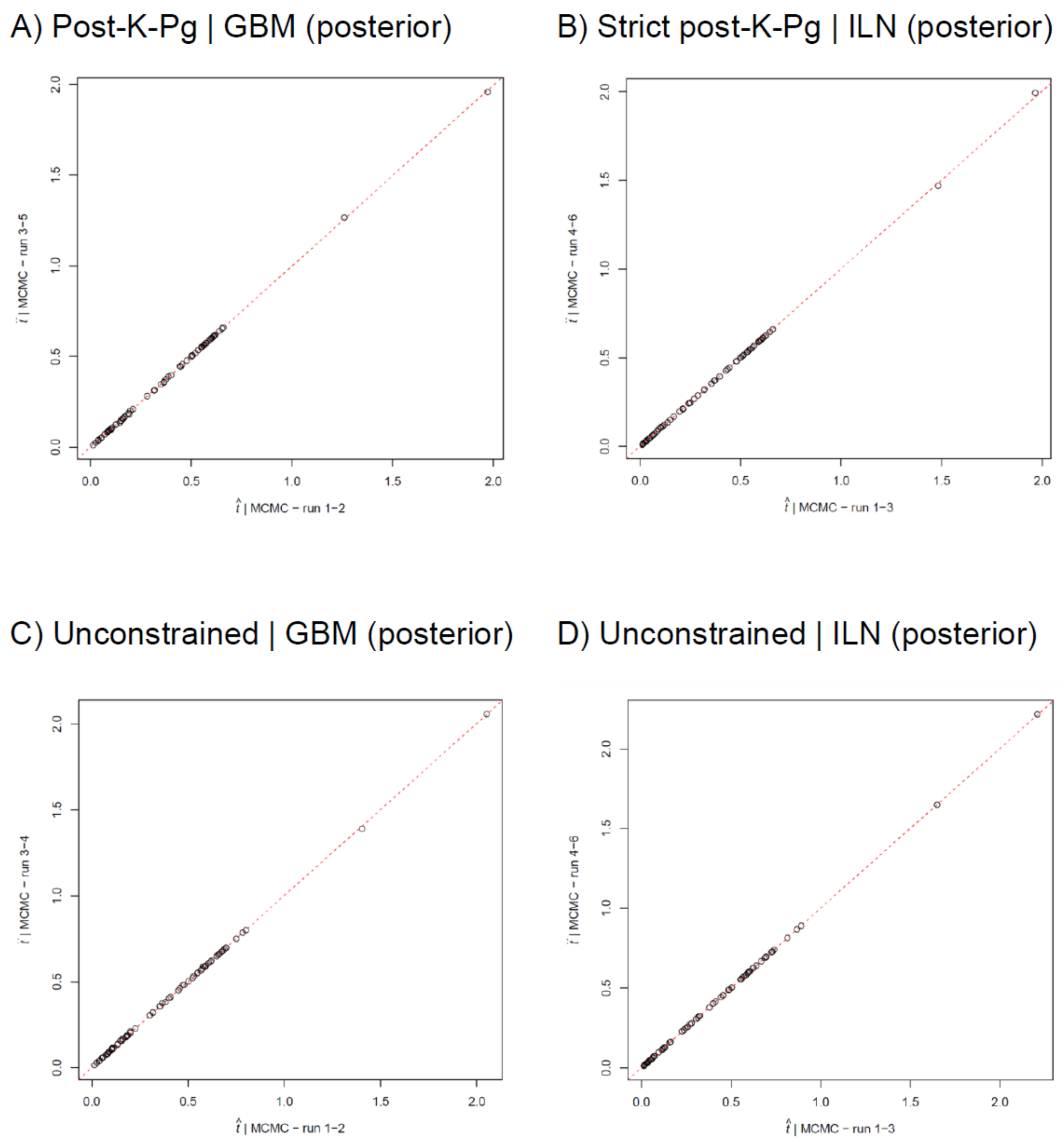

Figure S3. **Convergence plots for the summarised divergence times estimated under each of the diversification models testing the origin of placental mammals when data were used.** The mean divergence times were calculated using the chains that passed the filters during the MCMC diagnostics that were run for each diversification model and under both the geometric Brownian motion (GBM, autocorrelated rates) and the independent-rates

log-normal (ILN) relaxed-clock models when data were included (i.e., the target distribution from which sampling took place was the posterior distribution). The first row has the convergence plots for the Post-K-Pg model (A: GBM, B: ILN), and the last row for the Unconstrained model (C: GBM, D: ILN). The samples collected for half of the chains that passed the filters were summarised and the divergence times plotted on the x axis. The same was done for the other half; the divergence times were plotted on the y axis. Please note that not all chains were used to generate the converge plots for the analyses under GBM as some chains did not pass the filters (A: one chain did not pass the QC filters, C: two chains did not pass the QC filters). The convergence plots show that x ≃ y, and thus this qualitative check for chain convergence was passed for all analyses. Time unit is set to 100 Myr. The in-house R script used to obtain these plots generates one individual PDF file for each model. The script and output files are available in the GitHub repository. Path for script: “BayesModSel/00_mammals/02_rate_per_branch/01_MCMC_diagnostics/scripts/MCMC_diagnostics_posterior.R”. Path for output files: “BayesModSel/00_mammals/02_rate_per_branch/01_MCMC_diagnostics/sum_files_post/MCMC_diagnostics”.

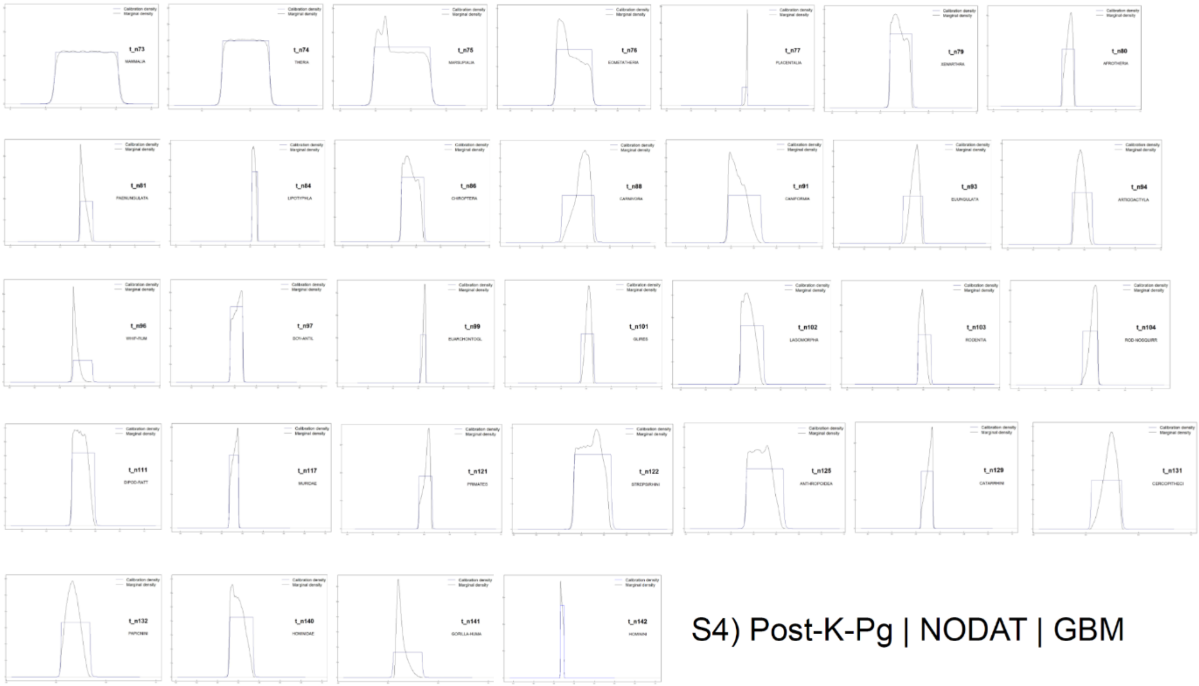

S4) Post-K-Pg | NODAT | GBM

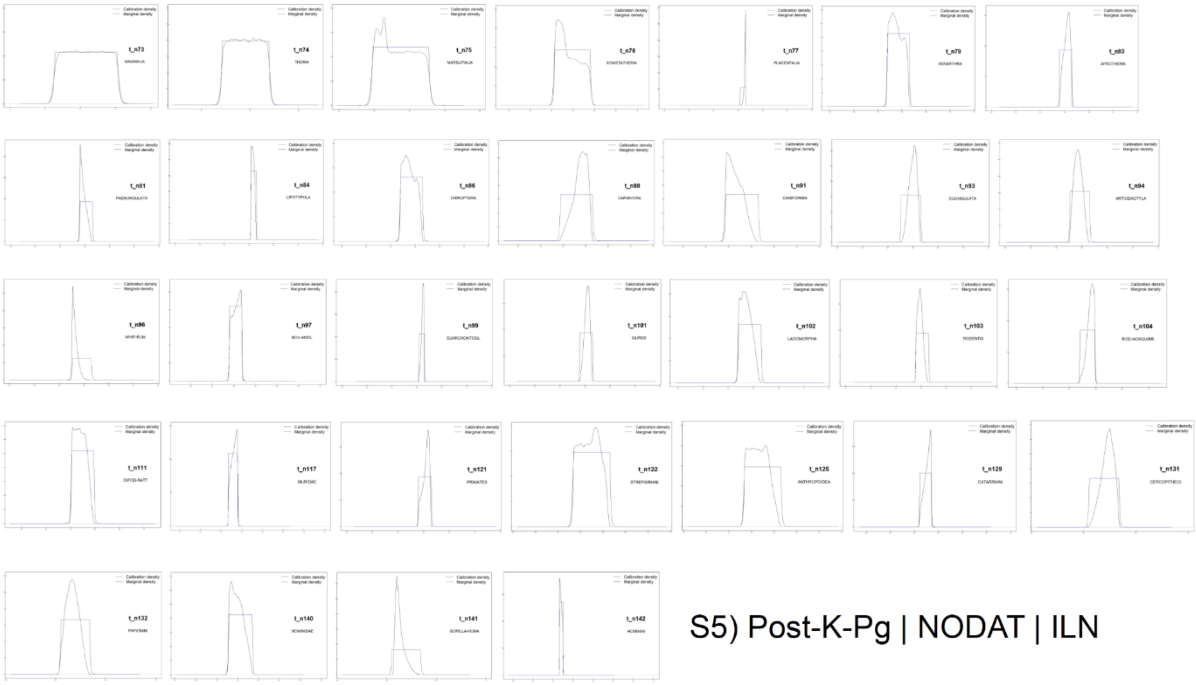

S5) Post-K-Pg | NODAT | ILN

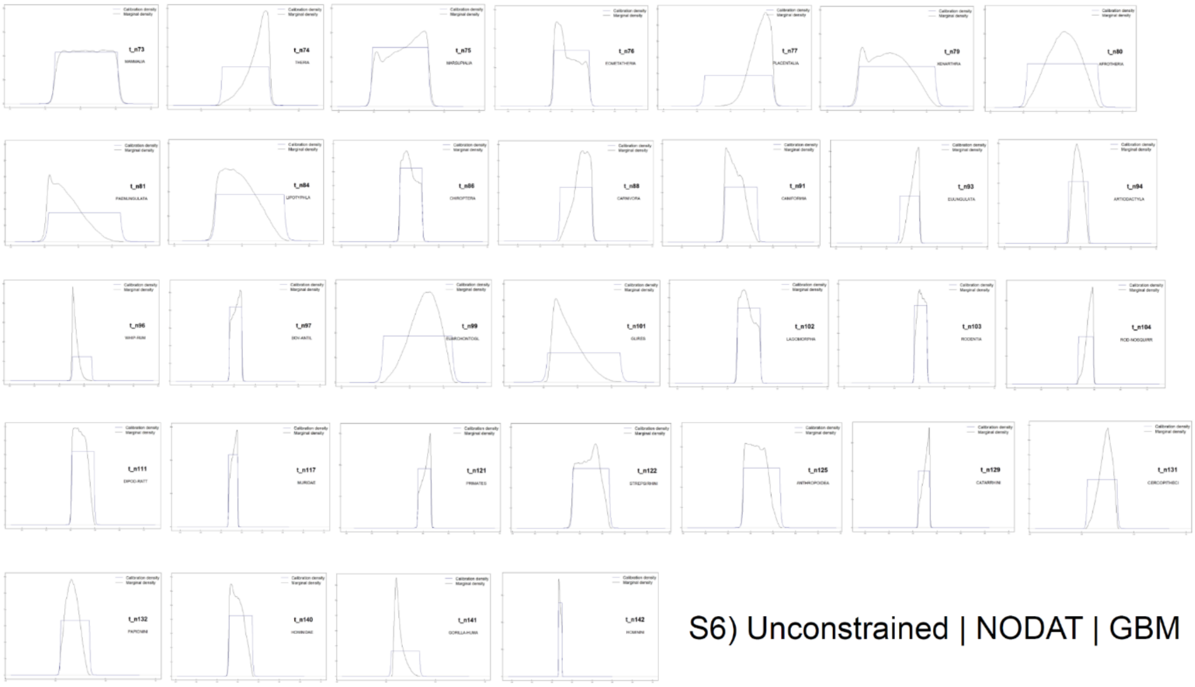

S6) Unconstrained | NODAT | GBM

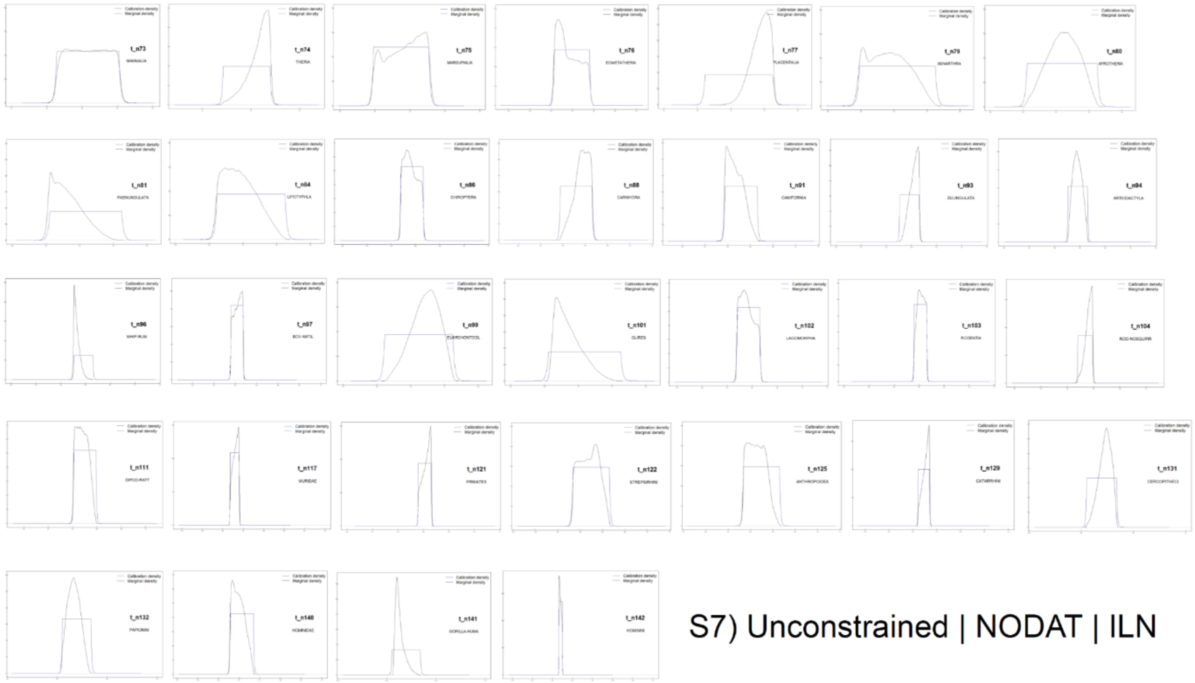

Figures S4-S7. **Calibration densities VS marginal densities for each of the diversification models testing the origin of placental mammals under each relaxed-clock model.** The marginal densities (black) were obtained when the target distribution was the prior (i.e., `usedata = 0` in the control file), while the calibration densities (blue) correspond to those specified by the user in the tree file (in this case, all of them uniform densities with either soft or hard bounds) to constrain the ages of specific nodes in the tree topology. S4: Post-K-Pg + GBM; S5: Post-K-Pg + ILN; S6: Unconstrained + GBM; S7: Unconstrained + ILN. Ordered from top to bottom and left to right, the following are the labels given to the nodes constrained (taxa have been included when the node label used in the analysis does not correspond to taxonomy): Mammalia (t_n73), Theria (t_n74), Marsupialia (t_n75), Eometatheria (t_n76), Placentalia (t_n77), Xenarthra (t_n79), Afrotheria (t_n80), Paenungulata (t_n81), Lipotyphla (t_n84), Chiroptera (t_n86), Carnivora (t_n88), Caniformia (t_n91), Euungulata (t_n93), Artiodactyla (t_n94), Whip-Rum (t_n96; *Tursiops truncatus, Bos taurus, Ovis aries, Capra hircus*), Bov-Antil (t_n97; *Bos taurus, Ovis aries, Capra hircus*), Euarchontoglires (t_n99), Glires (t_n101), Lagomorpha (t_n102), Rodentia (t_n103), Rod-nosquirrel (t_n104; *Fukomys damarensis, Heterocephalus glaber, Cavia porcellus, Cavia aperea, Chinchilla lanigera, Octodon degus, Dipodomys ordii, Jaculus jaculus, Nannospalax galili, Mesocricetus auratus, Cricetulus griseus, Peromyscus maniculatus, Microtus ochrogaster, Mus Pahari, Mus caroli, Mus musculus, Mus spretus, Rattus norvegicus*), Dipod-Ratt (t_n111; *Jaculus jaculus, Nannospalax galili, Mesocricetus auratus, Cricetulus griseus, Peromyscus maniculatus, Microtus ochrogaster, Mus Pahari, Mus caroli, Mus musculus, Mus spretus, Rattus norvegicus*), Muridae (t_n117), Primates (t_n121), Strepsirhini (t_n122), Anthropoidea (t_n125), Catarrhini (t_n129), Cercopithecinae (t_n131), Papionini (t_n132), Hominidae (t_n140), Gorilla-Human (t_n141; *Gorilla gorilla, Pan troglodytes, Pan paniscus, Homo sapiens*), Hominini (t_n142). The in-house R script used to obtain these plots generates one individual image for each calibrated node (available in the GitHub repository). All individual plots obtained under each model have been merged to generate figures S4-S7. The in-house R script used to obtain these plots generates one individual PDF file for each model. The script and output files are available in

the GitHub repository. Path for script: "BayesModSel/00_mammals/02_rate_per_branch/01_MCMC_diagnostics/scripts/Check_priors_margVScalib.R". Path for output files: "BayesModSel/00_mammals/02_rate_per_branch/01_MCMC_diagnostics/sum_files_prior/MCMC_diagnostics/margVScalib".

S8) Post-K-Pg | GBM

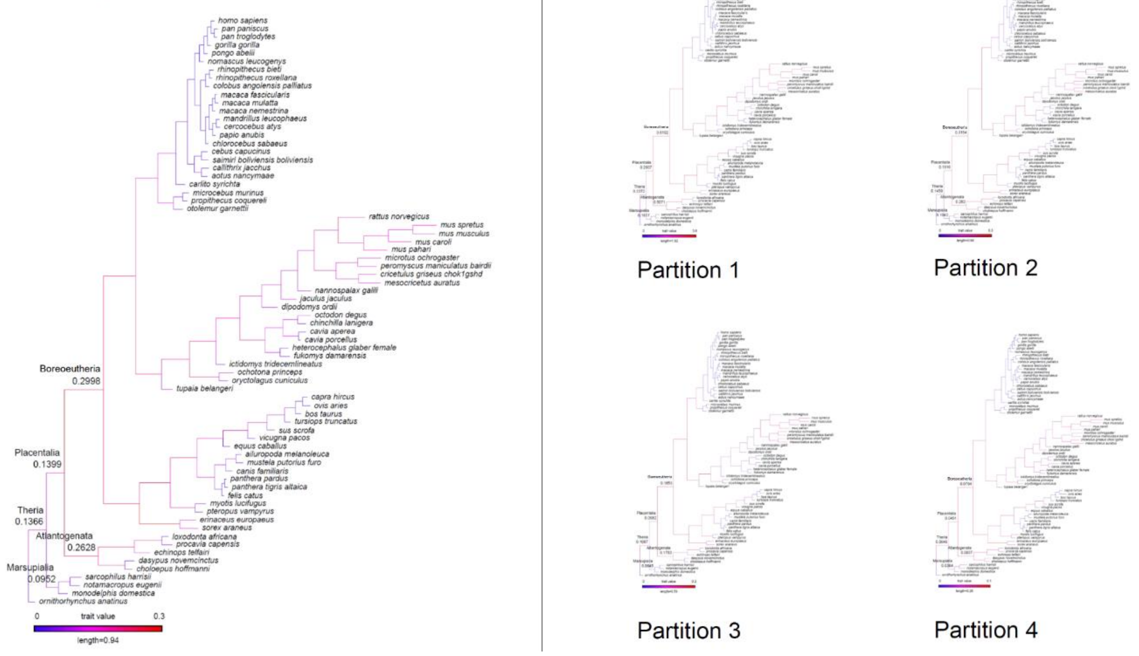

S9) Post-K-Pg | ILN

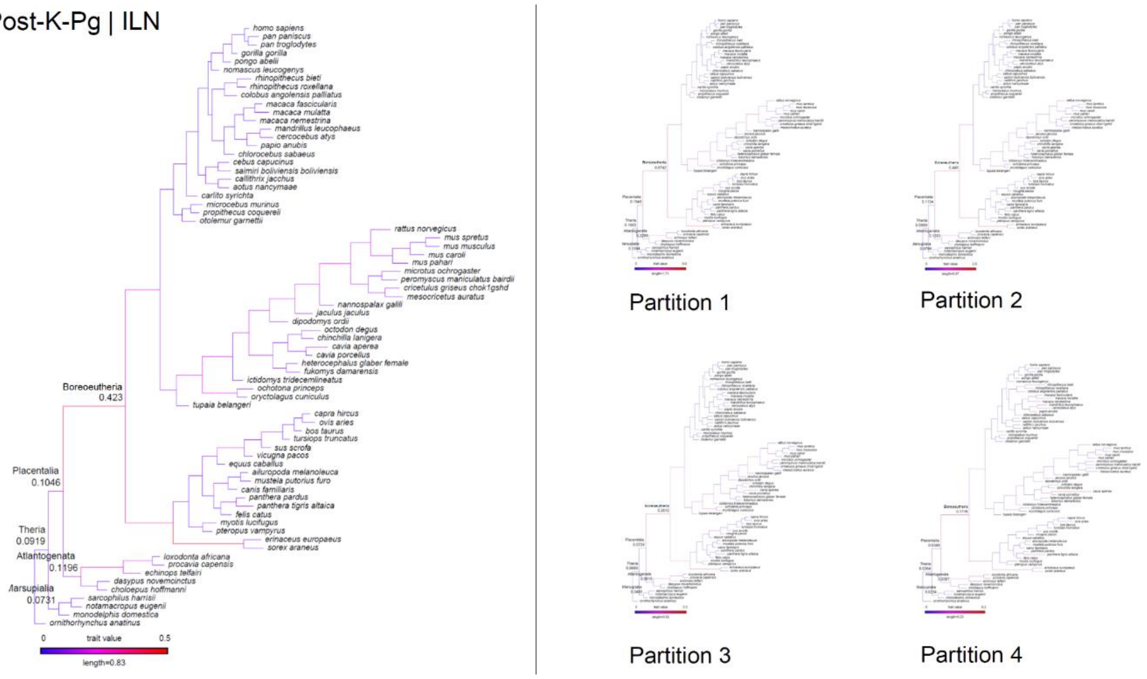

S10) Unconstrained | GBM

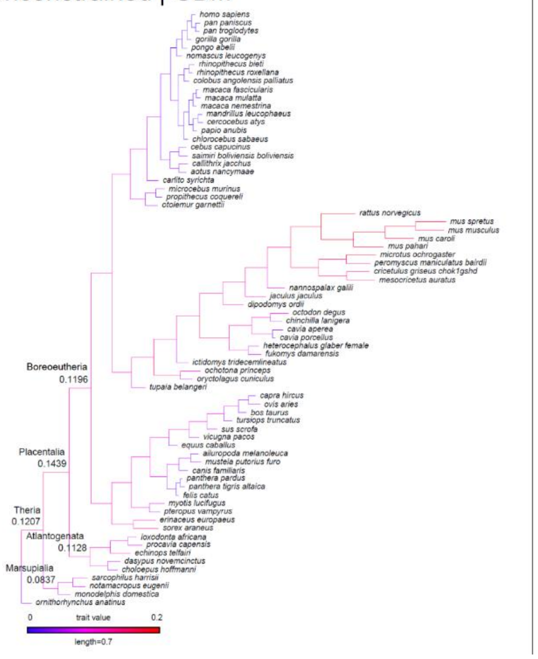

S11) Unconstrained | ILN

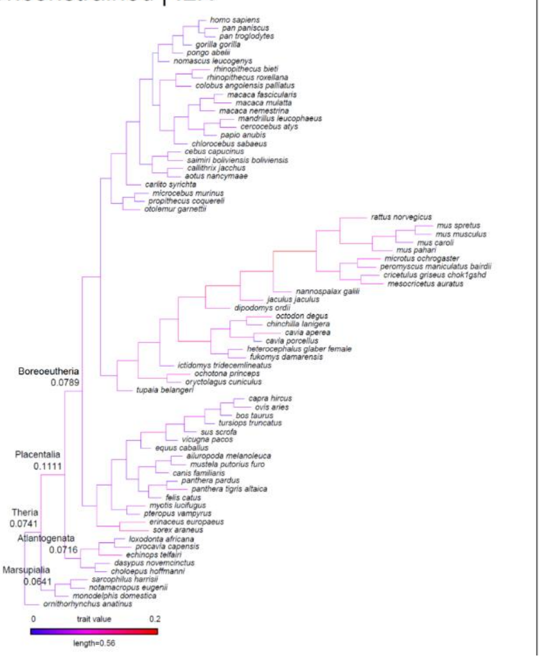

Figures S8-S11. Comparison of evolutionary rates estimated under the independent-rates log-normal (ILN) and Geometric Brownian motion (GBM) relaxed-clock models for each placental origination diversification model. There are two main sections in each figure. Left-hand side: average of the estimated evolutionary rates inferred for each of the four partitions of the sequence alignment. Right-hand side: estimated evolutionary rates inferred for each partition. S8: Post-K-Pg + GBM; S9: Post-K-Pg + ILN; S10: Unconstrained + GBM; S11: Unconstrained + ILN. The in-house R script used to obtain these plots generates one individual image for each partition and then for the rategram with averaged estimated evolutionary rates (available in the GitHub repository). All individual plots obtained under each model have been merged to generate figures S8-S11. The script and output files are available in the GitHub repository. Path for script: “BayesModSel/00_mammals/

02_rate_per_branch/02_Summary_Rates/scripts/Summarise_brates_mammals.R”. Path for output files: “BayesModSel/00_mammals/02_rate_per_branch/02_Summary_Rates/plots/rategrams”.

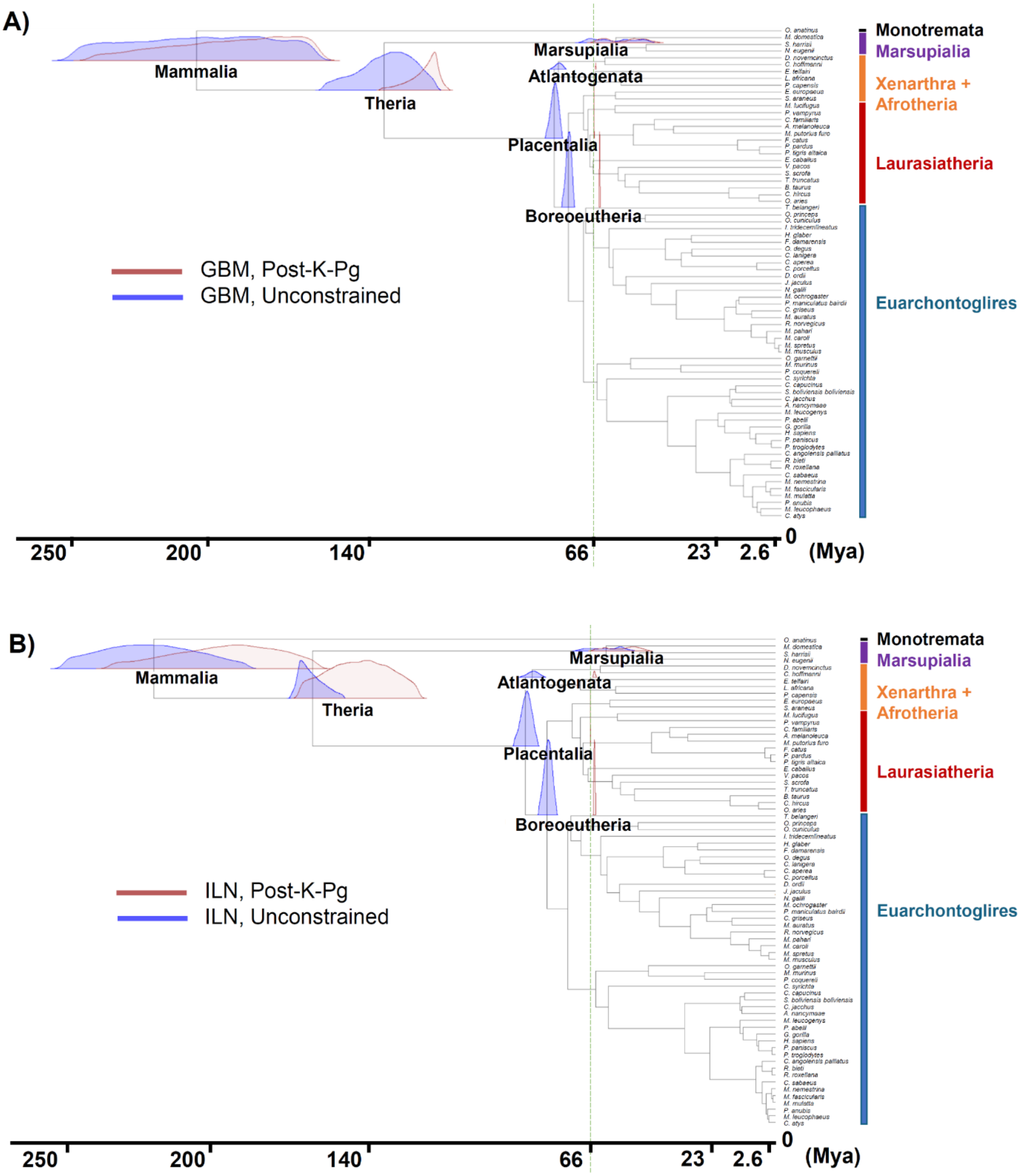

Figure S12. Estimate posterior time densities inferred under each of the diversification models for the origin of placental mammals under the geometric Brownian motion (GBM) and independent-rates log-normal (ILN) relaxed-clock models. This figure shows the summarised posterior time densities for nodes Mammalia, Theria, Marsupialia, Atlantogenata, Placentalia, and Boreoeutheria. The analyses took place under the GBM (A) and ILN (B) relaxed-clock models while assuming the node age constraints specified in the Unconstrained (blue) and Post K-Pg (brown) diversification models under a fixed tree topology (Álvarez-Carretero *et al.* 2022). The in-house R script used to obtain the evolutionary timelines with specific posterior densities generates one file

per relaxed-clock model being compared across all diversification models tested (relevant PDF files available in the GitHub repository). Additional features have been subsequently added to enhance the quality and understanding of the figure. The script and output files are available in the GitHub repository. Path for script: "BayesModSel/00_mammals/02_rate_per_branch/figs_tables/scripts/Plots_mammals_MCMCtreeR.R". Path for output files: "BayesModSel/00_mammals/02_rate_per_branch/figs_tables/plots". The in-house script used to generate the time densities integrates a modified version of one of MCMCtreeR's functions (Puttick 2019) to tailor the features being displayed here.

Table S1. Estimated divergence times and MCMC diagnostics that were carried out for all inference analyses with the mammal dataset. This spreadsheet contains various tabs with different results. Tabs "GBM|AllDivTimes" and "ILN|AllDivtimes" include a list with the posterior divergence times (Myr) and confidence intervals (2.5% and 97.5% quantiles) estimated for each diversification model (Post K-Pg and Unconstrained) under the geometric Brownian motion (GBM) and independent-rates log-normal (ILN) relaxed-clock models, respectively. Tabs "GBM|CalibNodesDivtimes" and "ILN|CalibNodesDivtimes" include the same information as "GBM|AllDivTimes" and "ILN|AllDivtimes", but only for those nodes which age had been constrained (i.e., calibrated nodes). All MCMC diagnostics for those chains which target distribution was the posterior are summarised in tab "MCMCdiagn|Post", while tab "MCMCdiagn|Prior" includes those when the target distribution was the prior. The stats regarding the tail-ESS values and the bulk-ESS (median, minimum, and maximum) were all larger than 100 when the target distribution was the prior; thus, the estimated parameter values are assumed to be reliable. The R-hat values reported for these chains were also smaller than 1.05, thus suggesting a good mixing. Nevertheless, the minimum bulk-ESS and minimum tail-ESS were lower than 100 for some of the chains when the target distribution was the posterior, which we could also see reflected in the R-hat values for some nodes (i.e., R-hat > 1.05; a better mixing may be required to improve the estimates of these divergence times). E.g.: amongst the various nodes listed in this tab, the estimated posterior time densities for nodes 126-128 seem to require a better mixing as they showed R-hat values larger than 1.05 for all the analyses we ran. The scripts that were used to summarise the divergence times and the MCMC diagnostics and the output files used to generate this table are available in the GitHub repository. Path for script: "BayesModSel/00_mammals/02_rate_per_branch/figs_tables/scripts". Path for output files: "BayesModSel/00_mammals/02_rate_per_branch/figs_tables/tables".

Abbreviations: *mean_t*: mean posterior time estimate; *2.5%q*: 2.5% quantile of the posterior time density for a given node; *97.5%q*: 97.5% quantile of the posterior time density for a given node; *med. num. samples collected per chain*: median of the total amount of samples collected per chain; *min. num. samples collected per chain*: minimum number of samples collected per chain; *max. num. samples collected per chain*: minimum number of samples collected per chain; *num. samples used to calculate stats*: number of samples collected by all chains that passed the filters that were used to calculate the tail-ESS, bulk-ESS and R-hat values.

MCMC diagnostics when running MCMCtree with the Metazoa dataset

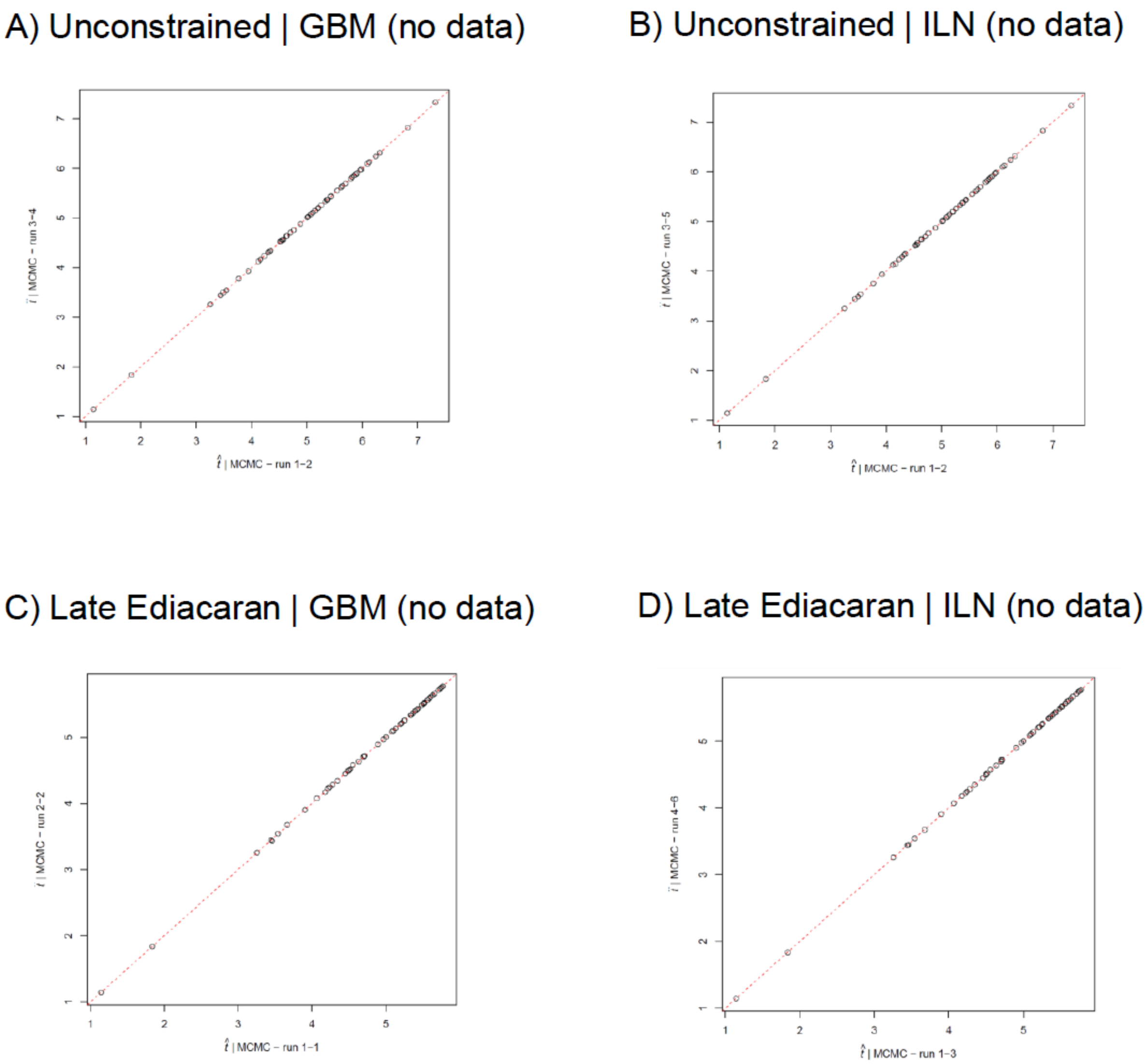

Figure S13. Convergence plots for the summarised divergence times estimated under each of the diversification models testing the origin of animals when data were not used. The mean divergence times were calculated using the chains that passed the filters during the MCMC diagnostics that were run for each diversification model and under both the geometric Brownian motion (GBM, autocorrelated rates) and the independent-rates log-normal (ILN) relaxed-clock models when data were not included (i.e., the target distribution from which sampling took place was the prior distribution). The first row has the convergence plots for the Unconstrained model (A: GBM, B: ILN) and the last row for the Late Ediacaran model (C: GBM, D: ILN). The samples collected for half of the chains that passed the filters were summarised and the divergence times plotted on the x axis. The same was done for the other half; the divergence times were plotted on the y axis. Please note that not all chains were used to generate the converge plots for the aforementioned analyses as some chains did not pass the filters (A: two chains did not pass the QC filters, B: one chain did not pass the QC filters, C: four chains did not pass the QC filters). All convergence plots show that x ≃ y, and thus this qualitative check for chain convergence was passed for all analyses. Time unit is set to 100 Myr. The in-house R script used to obtain these plots

generates one individual PDF file for each model. The script and output files are available in the GitHub repository. Path for script: “BayesModSel/ 01_animals/02_rate_per_branch/01_MCMC_diagnostics/scripts/ MCMC_diagnostics_prior.R”. Path for output files: “BayesModSel/ 01_animals/02_rate_per_branch/01_MCMC_diagnostics/sum_files_prior/MCMC_diagnostics ”.

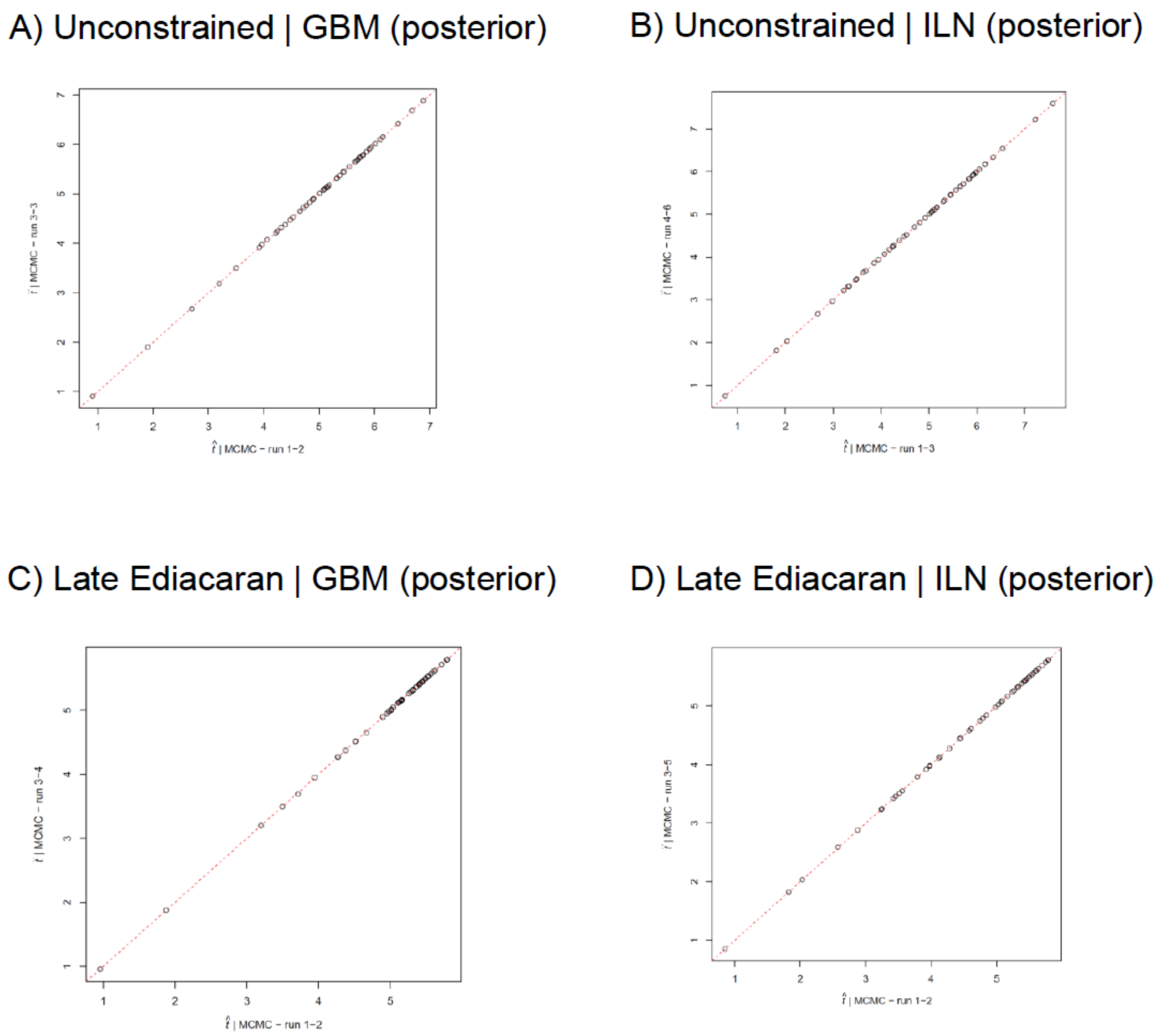

Figure S14. **Convergence plots for the summarised divergence times estimated under each of the diversification models testing the origin of animals when data were used.** The mean divergence times were calculated using the chains that passed the filters during the MCMC diagnostics that were run for each diversification model and under both the geometric Brownian motion (GBM, autocorrelated rates) and the independent-rates log-normal (ILN) relaxed-clock models when data were included (i.e., the target distribution from which sampling took place was the posterior distribution). The first row has the convergence plots for the Unconstrained model (A: GBM, B: ILN) and the last row for the Late Ediacaran model (C: GBM, D: ILN). The samples collected for half of the chains that passed the filters were summarised and the divergence times plotted on the x axis. The same was done for the other half; the divergence times were plotted on the y axis. Please note that not all chains were used to generate the converge plots for the aforementioned analyses as some

chains did not pass the filters (A: three chains did not pass the QC filters, C: two chains did not pass the QC filters, D: one chain did not pass the QC filters). All convergence plots show that x ≃ y, and thus this qualitative check for chain convergence was passed for all analyses. Time unit is set to 100 Myr. The in-house R script used to obtain these plots generates one individual PDF file for each model. The script and output files are available in the GitHub repository. Path for script: "BayesModSel/01_animals/02_rate_per_branch/01_MCMC_diagnostics/scripts/MCMC_diagnostics_posterior.R". Path for output files: "BayesModSel/01_animals/02_rate_per_branch/01_MCMC_diagnostics/sum_files_post/MCMC_diagnostics".

S15) Unconstrained | NODAT | GBM

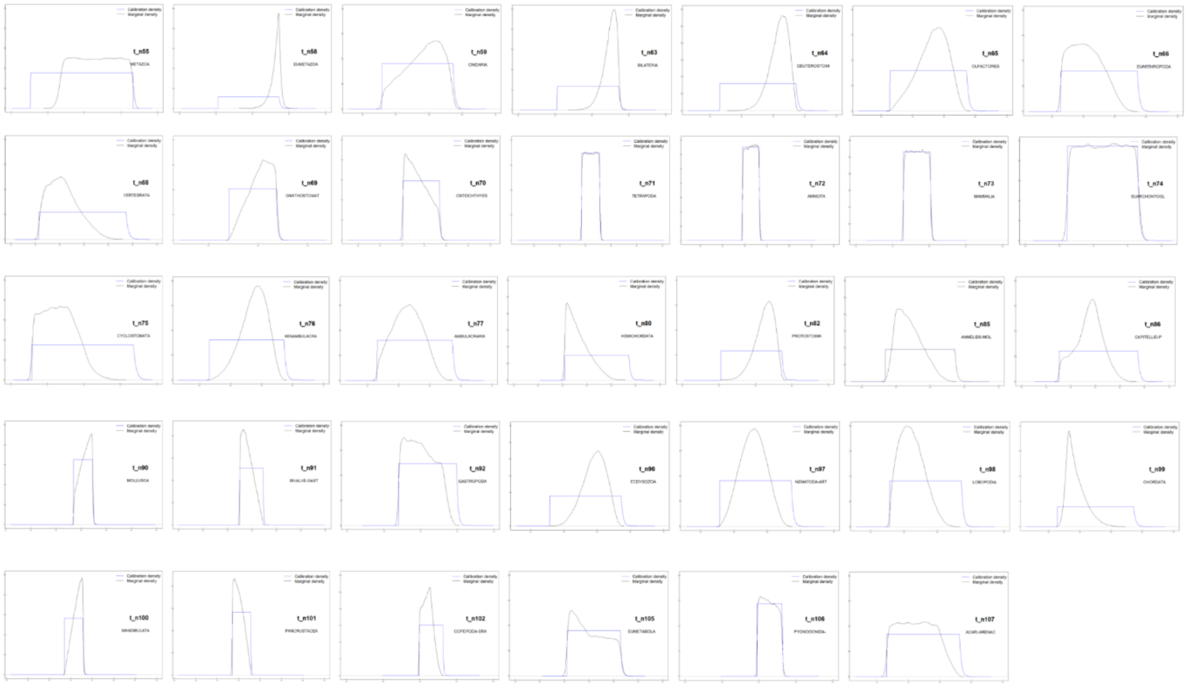

S16) Unconstrained | NODAT | ILN

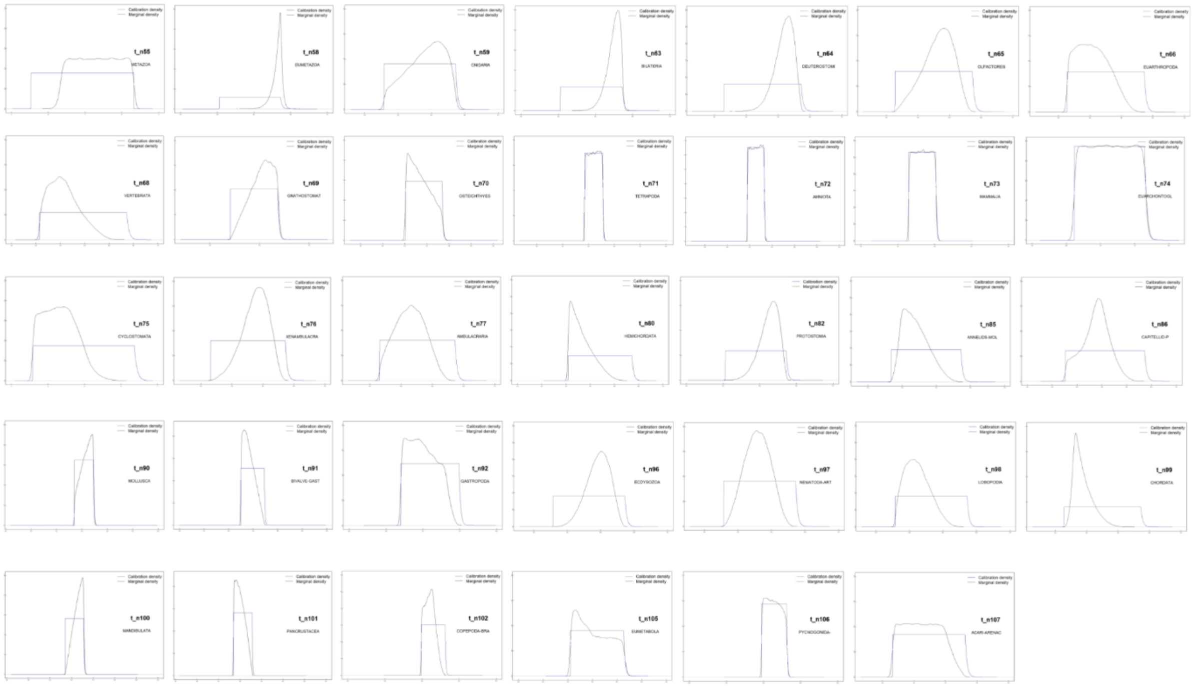

S17) Late Ediacaran | NODAT | GBM

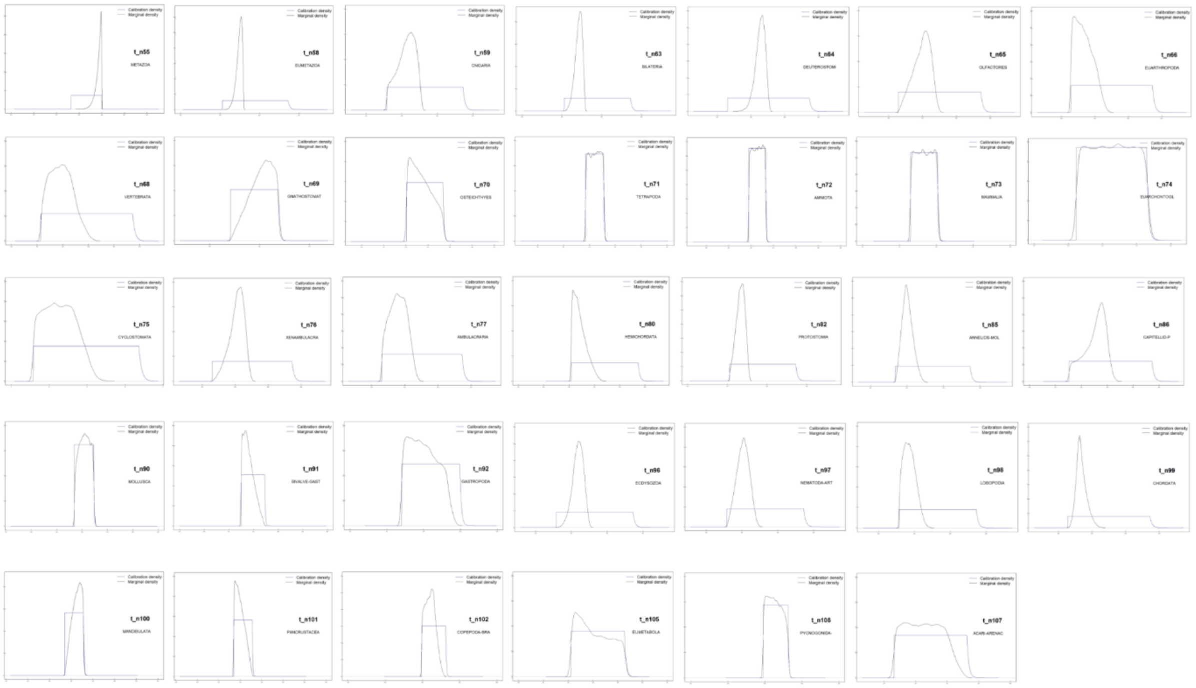

S18) Late Ediacaran | NODAT | ILN

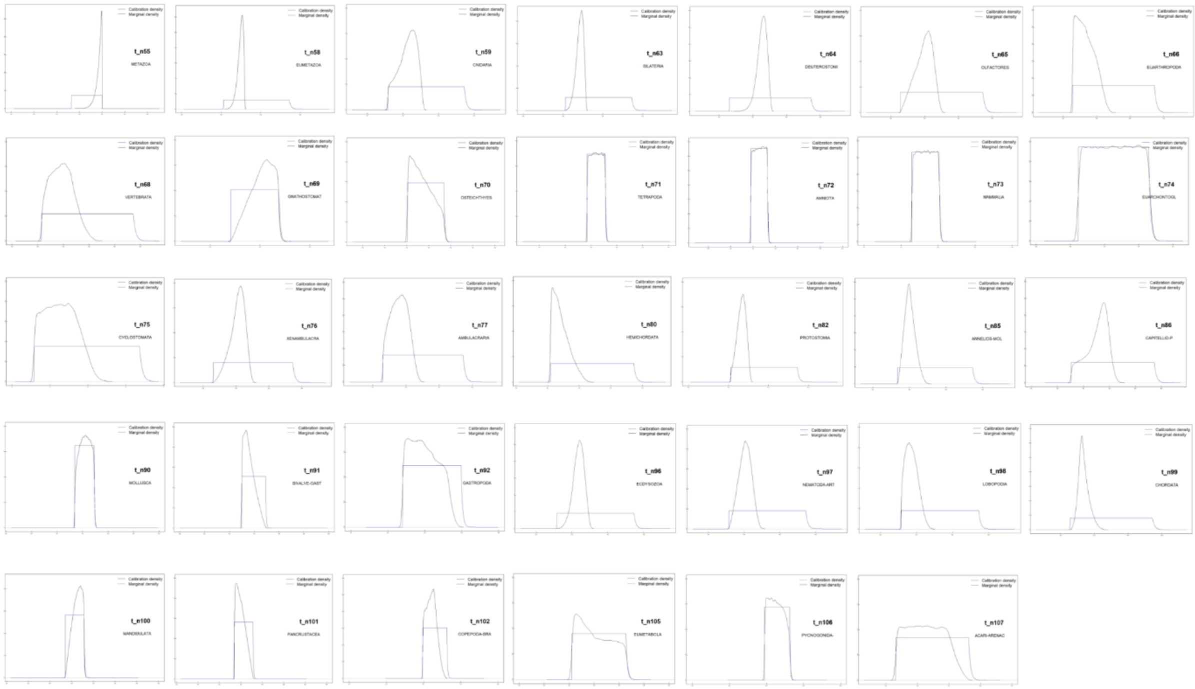

Figures S15-S18. **Calibration densities VS marginal densities for each of the diversification models testing the origin of animals under each relaxed-clock model.** The marginal densities (black) were obtained when the target distribution was the prior (i.e., `usedata = 0` in the control file), while the calibration densities (blue) correspond to those specified by the user in the tree file (in this case, all of them uniform densities with either soft or hard bounds) to constrain the ages of specific nodes in the tree topology. S15: Unconstrained + GBM; S16: Unconstrained + ILN; S17: Late Ediacaran + GBM; S18: Late Ediacaran + ILN. Ordered from top to bottom and left to right, the following are the labels given to the nodes constrained: Metazoa (t_n55), Eumetazoa (t_n58), Cnidaria (t_n59), Bilateria (t_n63), Deuterostomia (t_n64), Olfactores (t_n65), Euarthropoda (t_n66), Vertebrata (t_n68), Gnathostomata (t_n69), Osteichthyes (t_n70), Tetrapoda (t_n71), Amniota (t_n72), Mammalia (t_n73), Euarchontoglires (t_n74), Cyclostomata (t_n75), Xenambulacraria (t_n76), Ambulacraria (t_n77), Hemichordata (t_n80), Protostomia (t_n82), Annelids-Molluscs (t_n85), Capitellid-Polychete-Leech (t_n86), Mollusca (t_n90), Bivalve-Gastropod (t_n91), Gastropoda (t_n92), Ecdysozoa (t_n96), Nematoda-Arhtropoda (t_n97), Lobopodia (t_n98), Chordata (t_n99), Mandibulata (t_n100), Pancrustacea (t_n101), Copepoda-Branchiopoda (t_n102), Eumetabola (t_n105), Pycnogonida-other-Chelicerates (t_n106), Acari-Arenacea (t_n107). The in-house R script used to obtain these plots generates one individual image for each calibrated node (available in the GitHub repository). All individual plots obtained under each model have been merged to generate figures S15-S18. The in-house R script used to obtain these plots generates one individual PDF file for each model. The script and output files are available in the GitHub repository. Path for script: "BayesModSel/01_animals /02_rate_per_branch/01_MCMC_diagnostics/scripts/Check_priors_margVScalib.R". Path for output files: "BayesModSel/01_animas/02_rate_per_branch/01_MCMC_diagnostics/sum_files_prior/MCMC_diagnostics/margVScalib".

S19) Unconstrained | GBM

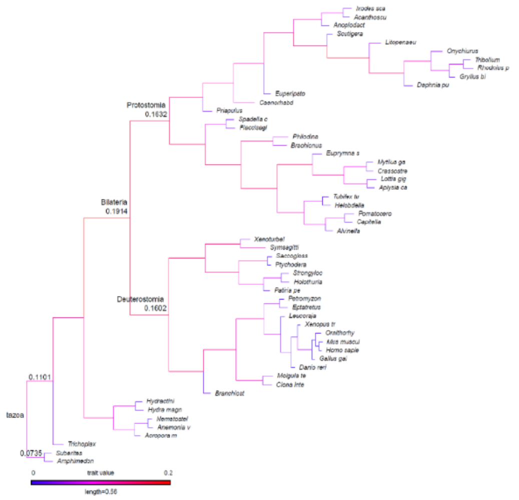

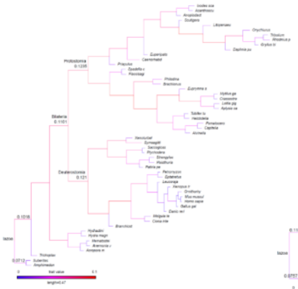

Partition 1

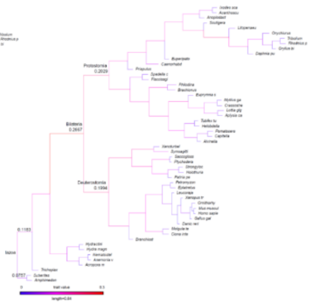

Partition 2

S20) Unconstrained | ILN

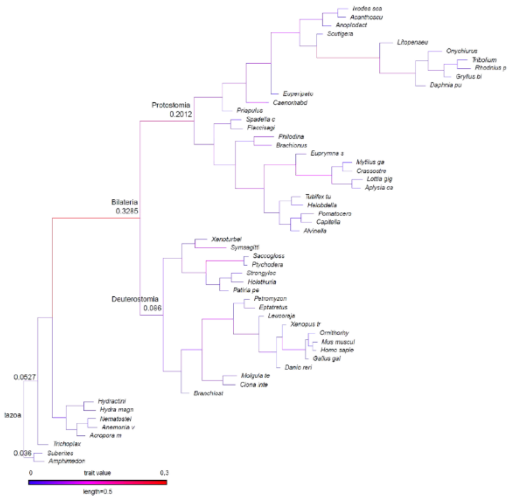

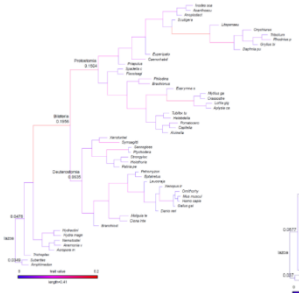

Partition 1

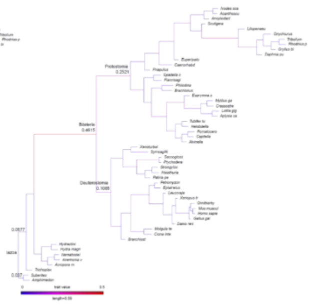

Partition 2

S21) Late Ediacaran | GBM

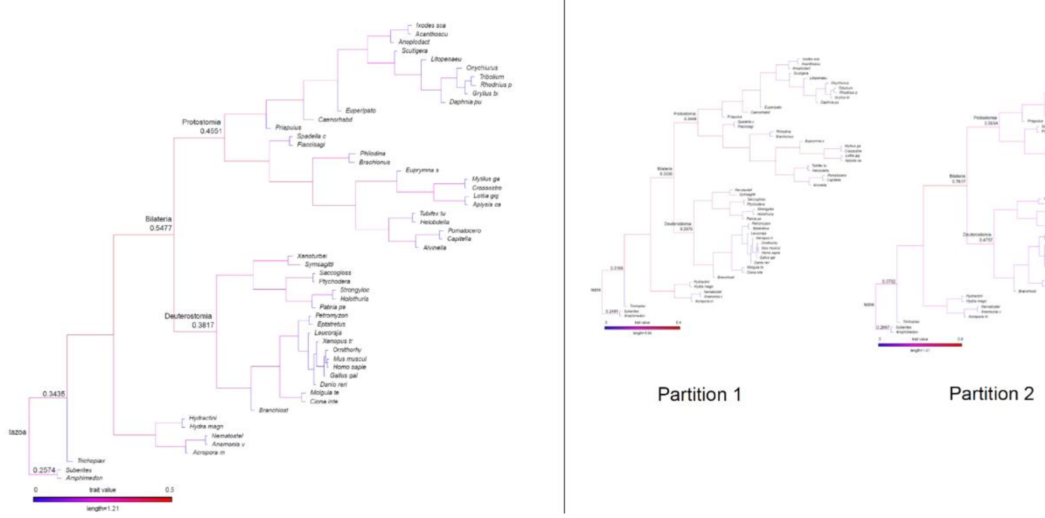

S22) Late Ediacaran | ILN

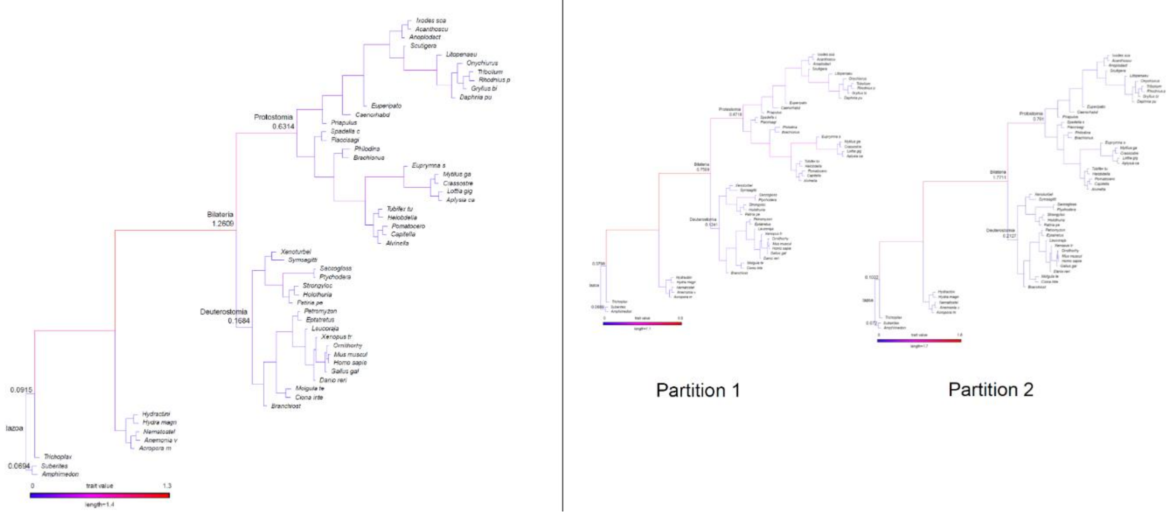

Figure S19-S22. Comparison of evolutionary rates estimated under the independent-rates log-normal (ILN) and Geometric Brownian motion (GBM) relaxed-clock models for each animal origination diversification model. There are two main sections in each figure. Left-hand side: average of the estimated evolutionary rates inferred for each of the four partitions of the sequence alignment. Right-hand side: estimated evolutionary rates inferred for each partition. S19: Unconstrained + GBM; S20: Unconstrained + ILN; S21: Late Ediacaran + GBM; S22: Late Ediacaran + ILN. The in-house R script used to obtain these plots generates one individual image for each partition and then for the rategram with averaged estimated evolutionary rates (available in the GitHub repository). All individual plots obtained under each model have been merged to generate figures S19-S22. The script and output files are available in the GitHub repository. Path for script: "BayesModSel/01_animals/ 02_rate_per_branch/02_Summary_Rates/scripts/ Summarise_brates_mammals.R". Path for output files: "BayesModSel/01_animals /02_rate_per_branch/02_Summary_Rates/plots/rategrams".

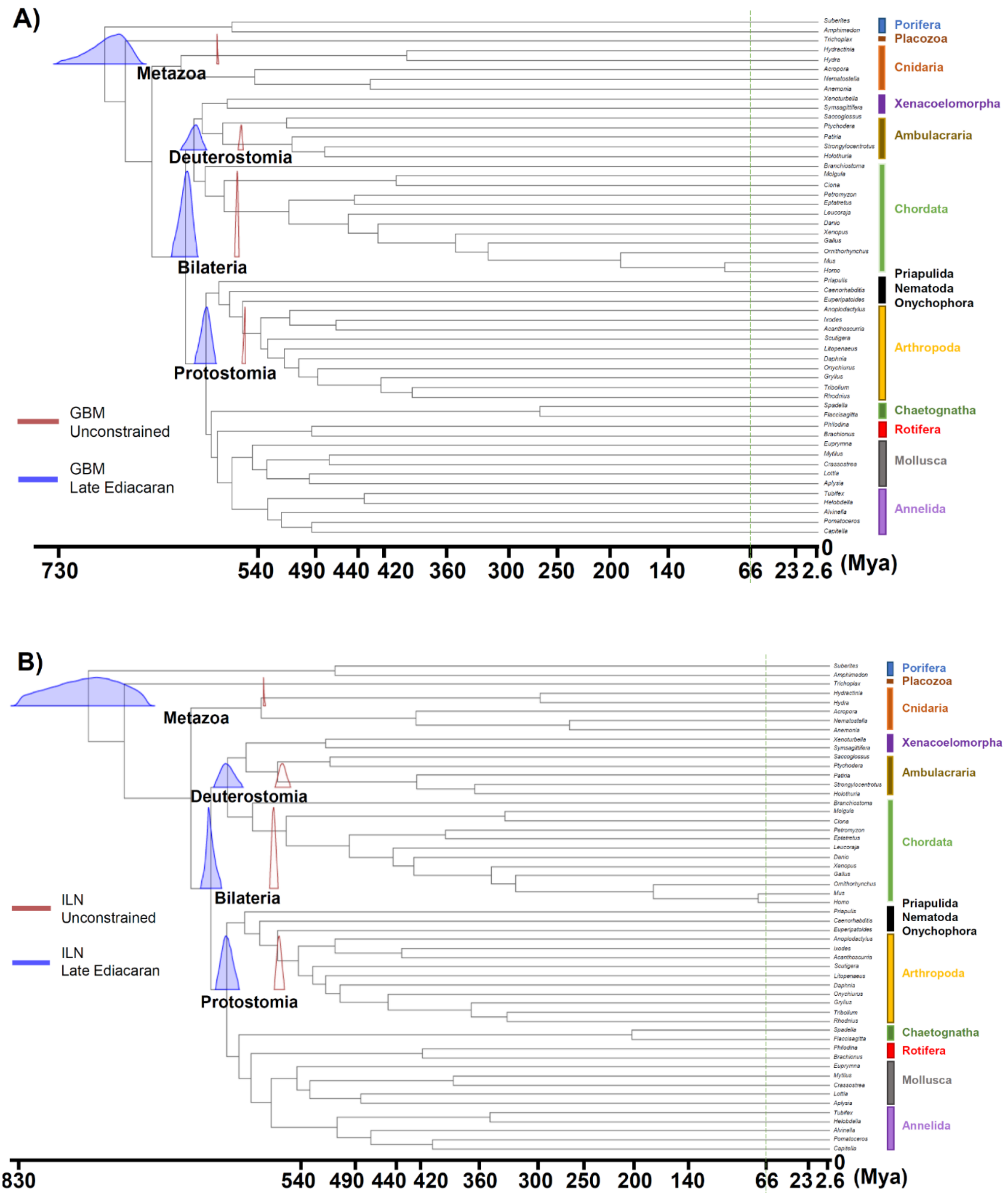

Figure S23. Estimated posterior time densities inferred under each of the diversification models for the origin of animals under the geometric Brownian motion (GBM) relaxed-clock model. This figure shows the summarised posterior time densities for nodes Metazoa, Deuterostomia, Bilateria, and Protostomia. The analyses took place under the GBM (A) and ILN (B) relaxed-clock models while assuming the node age constraints specified in the Unconstrained (blue) and Late Ediacaran (brown) diversification models under a fixed tree topology (dos Reis *et al.* 2015). The in-house R script used to obtain the evolutionary timelines with specific posterior densities generates one file per relaxed-clock model being compared across all diversification models tested (relevant PDF files available in the GitHub repository). Additional features have been subsequently added to enhance the quality and understanding of the figure. The script and output files are available in the

GitHub repository. Path for script: “BayesModSel/01_animals/
02_rate_per_branch/figs_tables/scripts/ Plots_mammals_MCMCtreeR.R”. Path for output files: “BayesModSel/01_animals/02_rate_per_branch/figs_tables/plots”. The in-house script used to generate the time densities integrates a modified version of one of MCMCtreeR’s functions (Puttick 2019) to tailor the features being displayed here.

Table S2. Estimated divergence times and MCMC diagnostics that were carried out for all inference analyses when analysing the animals dataset. This spreadsheet contains various tabs with different results. Tabs “GBM|AllDivTimes” and “ILN|AllDivtimes” include a list with the posterior divergence times (Myr) and confidence intervals (2.5% and 97.5% quantiles) estimated for each diversification model (Unconstrained and Later Ediacaran) under the geometric Brownian motion (GBM) and independent-rates log-normal (ILN) relaxed-clock models, respectively. Tabs “GBM|CalibNodesDivtimes” and “ILN|CalibNodesDivtimes” include the same information as “GBM|AllDivTimes” and “ILN|AllDivtimes”, but only for those nodes which age had been constrained (i.e., calibrated nodes). All MCMC diagnostics for those chains which target distribution was the posterior are summarised in tab “MCMCdiagn|Post”, while tab “MCMCdiagn|Prior” includes those when the target distribution was the prior. The stats regarding the tail-ESS values and the bulk-ESS (median, minimum, and maximum) were all larger than 100 when the target distribution was the prior and the posterior; thus, the estimated parameter values are assumed to be reliable. The R-hat values reported for all chains were also smaller than 1.05, thus suggesting a good mixing. The scripts that were used to summarise the divergence times and the MCMC diagnostics and the output files used to generate this table are available in the GitHub repository. Path for script: “BayesModSel/01_animals/
02_rate_per_branch/figs_tables/scripts”. Path for output files: “BayesModSel/01_animals
/02_rate_per_branch/figs_tables/tables”.

Abbreviations: *mean_t*: mean posterior time estimate; *2.5%q*: 2.5% quantile of the posterior time density for a given node; *97.5%q*: 97.5% quantile of the posterior time density for a given node; *med. num. samples collected per chain*: median of the total amount of samples collected per chain; *min. num. samples collected per chain*: minimum number of samples collected per chain; *max. num. samples collected per chain*: minimum number of samples collected per chain; *num. samples used to calculate stats*: number of samples collected by all chains that passed the filters that were used to calculate the tail-ESS, bulk-ESS and R-hat values.

The evolutionary gap in the fossil record of placental mammals

In the main text, we state that there is a gap of ~780 Myr in the fossil record of placental mammals based on Figure 1 in O’Leary *et al.* (2013). Estimating the length of the branches (in Myr) depicting ghost lineages, however, was not an easy task due to the irregular breaking points in the accompanying timeline.

While one would expect the length of the breaking points in the timescale to be constant (e.g., same distance for every 10 Myr), Figure 1 does not follow this logic. For instance, the distance spanning 55-65 Myr (i.e., 10 Myr) is much longer than the distance allocated for the time period ranging from 65 to 145 Myr (i.e., 80 Myr). One can find more of such examples in that timeline. To figure out the length of each branch, we manually mapped the beginning

and end of each of the orange branches (i.e., ghost lineages in their Figure 1) to the irregular timeline; the difference was the estimated time spanning each ghost lineage.

We provide the R script we used for this calculation, “count_ghostlineages.R”, as part of our Supplementary Material. The script includes comments to help the reader understand what each object is and to which ghost lineage they correspond to in Figure 1 (O’Leary *et al.* 2013).

Order statistics and truncation

A consequence of the ordered nature of node ages is their prior distributions will be truncated. That is, the probability mass in the area in which a parent node is younger than a daughter node must be set to zero. For example, consider the ages of the two internal nodes, t_2 and t_3, in the phylogeny of Fig. S24. Assume a uniform calibration density is applied on each, between 0 (present time) and the age of the root: $f(t_2) = f(t_3) = 1/t_1$. Their joint prior density, $f(t_2, t_3) = 1/t_1^2$, which must respect the constraint $0 < t_3 < t_2 < t_1$, resembles a tridimensional wedge after truncation (Fig. S24b). If we construct an MCMC chain to sample this distribution, we will notice that the chain will spend relatively little time at the diagonal corners of the wedge, because those corners contain little probability mass: i.e., the corner in which t_3 (the youngest node) is near the root, and the corner in which t_2 (the oldest of the two nodes) is near 0. The result is that the marginal prior densities of the node ages cannot longer be uniform but, instead, will reflect the increasingly reduced probability mass towards the corners (Fig. S24c). In other words, the calibration densities (which are uniform) are very different from the marginal (or effective) prior densities (Inoue *et al.* 2010; Warnock *et al.* 2015; Donoghue and Yang 2016). This truncation is neither abnormal nor pathological; it is simply the consequence of the ordered nature of the variables: order statistics with a joint uniform prior distribution cannot have marginal uniform prior densities. In the case of a joint uniform density on order statistics, truncation is inconsequential because the joint uniform density guarantees MCMC proposals towards the corners are not penalised. This can be easily verified by calculating the proposal ratio of MCMC moves, $t \to t'$:

$$\alpha = \frac{f(t_2, t_3')}{f(t_2, t_3)} \times \frac{f(D|t_2, t_3', r)}{f(D|t_2, t_3, r)} = \frac{f(D|t_2, t_3', r)}{f(D|t_2, t_3, r)}$$

i.e., the joint prior density cancels out in the proposal and only the likelihood ratio, $f(D|t_2, t_3', r)/f(D|t_2, t_3, r)$, drives the move.

In the mammal analysis of Álvarez-Carretero *et al.* (2022), a multidimensional joint uniform prior is constructed for the age of Placentalia and its descendant nodes. This joint prior is, due to truncation, a multidimensional wedge. The resulting marginal prior density on Placentalia has vanishingly small probability mass near the K-Pg event, and this was interpreted by Budd and Mann (2024) as a pathological prior that precludes the possibility of post-K-Pg origination for the crown group. However, this interpretation is incorrect because the multidimensional prior is still uniform, and MCMC proposals across this multidimensional wedge are not penalised, allowing the posterior to settle in the correct position in parameter space given the branch lengths in the phylogeny.

Finally, we note truncation cannot be "corrected". If we were to insist, for example, on one of the order statistics having a uniform marginal prior, then the joint prior would no longer be uniform. For example, in the case of Fig. S24, this can be achieved by making the density of one of the variables conditional on the other one, which results in a joint prior with a large peak in a corner (Fig. S24d). This new joint prior does not longer cancel out in MCMC proposals, and the joint prior effectively pulls proposals towards the corner with high probability mass, to compensate for truncation and force the marginal uniform density on the age of interest (Fig. S24e).

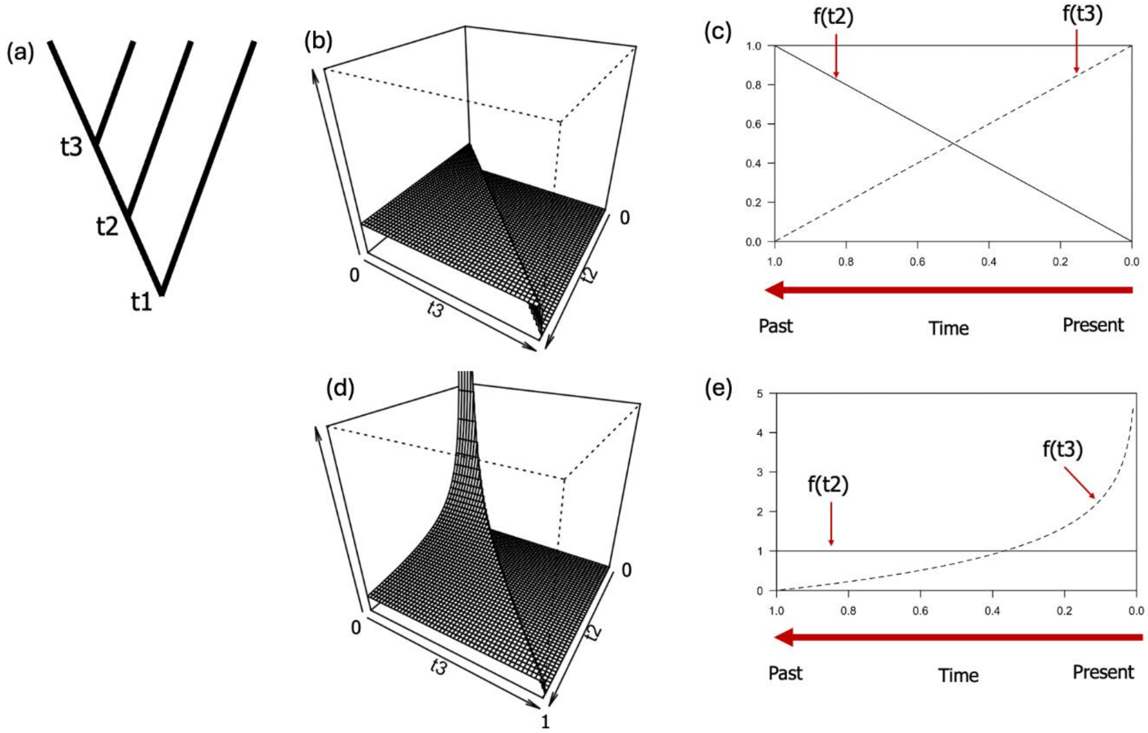

Figure S24. **Order statistics and truncation.** (a) Phylogeny of four species with three internal nodes. The age of the root, $t_1 = 1$, is fixed in all cases. (b) Joint density on the ages t_2 and t_3 is a truncated uniform density, $f(t_2, t_3) = 1/t_1^2$, $t_1 \geq t_2 \geq t_3$. (c) The joint uniform density results on non-uniform marginal densities for t_2 and t_3. (d) Joint density on t_2 and t_3 is $f(t_2, t_3) = f(t_3, t_2)f(t_2) = 1/t_2 \times 1/t_1$, $t_1 \geq t_2 \geq t_3$. This joint density has an infinitely high peak at (0, 0). (e) The non-uniform joint density guarantees that t_2 has a uniform marginal density, as the peak at (0, 0) compensates for the truncation effect. This joint prior density no longer cancels out in MCMC proposals.

Phylopic artwork

The artwork used in Fig. 3 has been dedicated to the public domain and can be accessible via Phylopic: https://www.phylopic.org/images/

We accessed Phylopic on 2025/10/10 to download each of the images we display on Fig. X. The links we accessed are the following:

- **Lagomorpha**: https://www.phylopic.org/images/ad7d2a9d-ef0d-46f2-895d-7c67bb8e6355/oryctolagus-cuniculus

- **Rodentia**: https://www.phylopic.org/images/3b89954e-b28e-4062-89cd-1ad5df0eb431/apodemus-sylvaticus
- **Primates**: https://www.phylopic.org/images/20c8c1f6-6e7d-4a0a-aaa9-d363d93c527c/cercopithecinae
- **Dermoptera**: https://www.phylopic.org/images/31c7a540-3d0a-4bb8-a43d-e6fa460a3c1d/galeopterus-variegatus
- **Scandentia**: https://www.phylopic.org/images/88a07585-846a-405d-9195-c15c010e7443/ptilocercus-lowii
- **Cetartiodactyla**: https://www.phylopic.org/images/4b474bdd-24e0-428d-b1f0-6d221ffc6367/odocoileus-virginianus
- **Perissodactyla**: https://www.phylopic.org/images/071ee517-a0f1-4d19-aa29-812b9f86cb53/equus-ferus-caballus
- **Carnivora**: https://www.phylopic.org/images/d67d3bf6-3509-4ab6-819a-cd409985347e/vulpes-vulpes
- **Pholidota**: https://www.phylopic.org/images/f628f6fe-6e2d-4173-a20f-265dc7d2d04f/manis-culionensis
- **Chiroptera**: https://www.phylopic.org/images/805e756d-81f2-449f-a9ef-662708f6399a/plecotus-austriacus
- **Eulipotyphla**: https://www.phylopic.org/images/f6146c1d-874f-45a8-9d5c-eff0d9df0802/desmana-moschata
- **Afrosoricida**: https://www.phylopic.org/images/181fcea5-6a69-40c3-b14e-f638f77ac5ce/potamogale-velox
- **Macroscelidea**: https://www.phylopic.org/images/b2a34ebf-1eab-4ddc-a926-af305a20828b/petrodromus-tetradactylus
- **Tubulidentata**: https://www.phylopic.org/images/5d3926b8-53b9-4382-8dd6-eaa273c79113/orycteropus-afer
- **Sirenia**: https://www.phylopic.org/images/d88fc903-0deb-4b78-b2fd-7c15d206d3f9/trichechus-senegalensis
- **Hyracoidea**: https://www.phylopic.org/images/bae84fd8-937a-4390-b123-0a22bfcd7df7/dendrohyrax
- **Proboscidea**: https://www.phylopic.org/images/db278f3b-d8c1-47e9-afce-0478980f5875/elephas-maximus
- **Pilosa**: https://www.phylopic.org/images/361913c8-6f63-412e-9792-da4651619454/choloepus
- **Cingulata**: https://www.phylopic.org/images/05351855-f406-47c8-b11b-9d38d681bb8b/priodontes-maximus

Data Availability

The code, data, and step-by-step guidelines to reproduce our analyses are available at https://github.com/sabifo4/BayesModSel. Large output MCMC files are available at https://figshare.com/s/badcc59a192fa4fd0722. The original alignments for the animal and mammal datasets are available at https://doi.org/10.6084/m9.figshare.1525089 and https://doi.org/10.6084/m9.figshare.14885691, respectively.

References

Álvarez-Carretero S., Tamuri A.U., Battini M., Nascimento F.F., Carlisle E., Asher R.J., Yang Z., Donoghue P.C.J., dos Reis M. 2022. A species-level timeline of mammal evolution integrating phylogenomic data. Nature. 602:263–267.

Budd G.E., Mann R.P. 2024. Two notorious nodes: a critical examination of relaxed molecular clock age estimates of the bilaterian animals and placental mammals. Syst. Biol. 73:223–234.

Donoghue P.C.J., Yang Z. 2016. The evolution of methods for establishing evolutionary timescales. Philos. Trans. R. Soc. Lond. B. Biol. Sci. 371:20160020.

dos Reis M., Thawornwattana Y., Angelis K., Telford M.J., Donoghue P.C.J., Yang Z. 2015. Uncertainty in the timing of origin of animals and the limits of precision in molecular timescales. Curr. Biol. 25:2939–2950.

dos Reis M., Gunnell G.F., Barba-Montoya J., Wilkins A., Yang Z., Yoder A.D. 2018. Using phylogenomic data to explore the effects of relaxed clocks and calibration strategies on divergence time estimation: primates as a test case. Syst. Biol. 67:594–615.

Inoue J., Donoghue P.C.J., Yang Z. 2010. The impact of the representation of fossil calibrations on Bayesian estimation of species divergence times. Syst. Biol. 59:74–89.

Jeffreys H. 1935. Some tests of significance, treated by the Theory of Probability. Math. Proc. Camb. Philos. Soc. 31:203–222.

Lartillot N., Philippe H. 2006. Computing Bayes Factors using thermodynamic integration. Syst. Biol. 55:195–207.

O'Leary M.A., Bloch J.I., Flynn J.J., Gaudin T.J., Giallombardo A., Giannini N.P., Goldberg S.L., Kraatz B.P., Luo Z.-X., Meng J., Ni X., Novacek M.J., Perini F.A., Randall Z.S., Rougier G.W., Sargis E.J., Silcox M.T., Simmons N.B., Spaulding M., Velazco P.M., Weksler M., Wible J.R., Cirranello A.L. 2013. The placental mammal ancestor and the post–K-Pg radiation of placentals. Science. 339:662–667.

Puttick M.N. 2019. MCMCtreeR: functions to prepare MCMCtree analyses and visualize posterior ages on trees. Bioinformatics. 35:5321–5322.

Warnock R.C.M., Parham J.F., Joyce W.G., Lyson T.R., Donoghue P.C.J. 2015. Calibration uncertainty in molecular dating analyses: there is no substitute for the prior evaluation of time priors. Proc. R. Soc. B Biol. Sci. 282:20141013.

Xie W., Lewis P.O., Fan Y., Kuo L., Chen M.-H. 2011. Improving marginal likelihood estimation for Bayesian phylogenetic model selection. Syst. Biol. 60:150–160.

Yang Z. 2007. PAML 4: phylogenetic analysis by maximum likelihood. Mol. Biol. Evol. 24:1586–1591.
