# Supplementary material for: On the origin of animals and placental mammals: a critique of literalist readings of the fossil record
Source: Mol Biol Evol. 2026 Aug 19;43(9):msag211. doi: 10.1093/molbev/msag211 (PMC13543917; doi:10.1093/molbev/msag211)
Supplement: Supplementary file 1 [file msag211_supplementary_data.zip › Supp_Fig_S12_Mammals_CompareDistsDivModelsPlots.pdf]

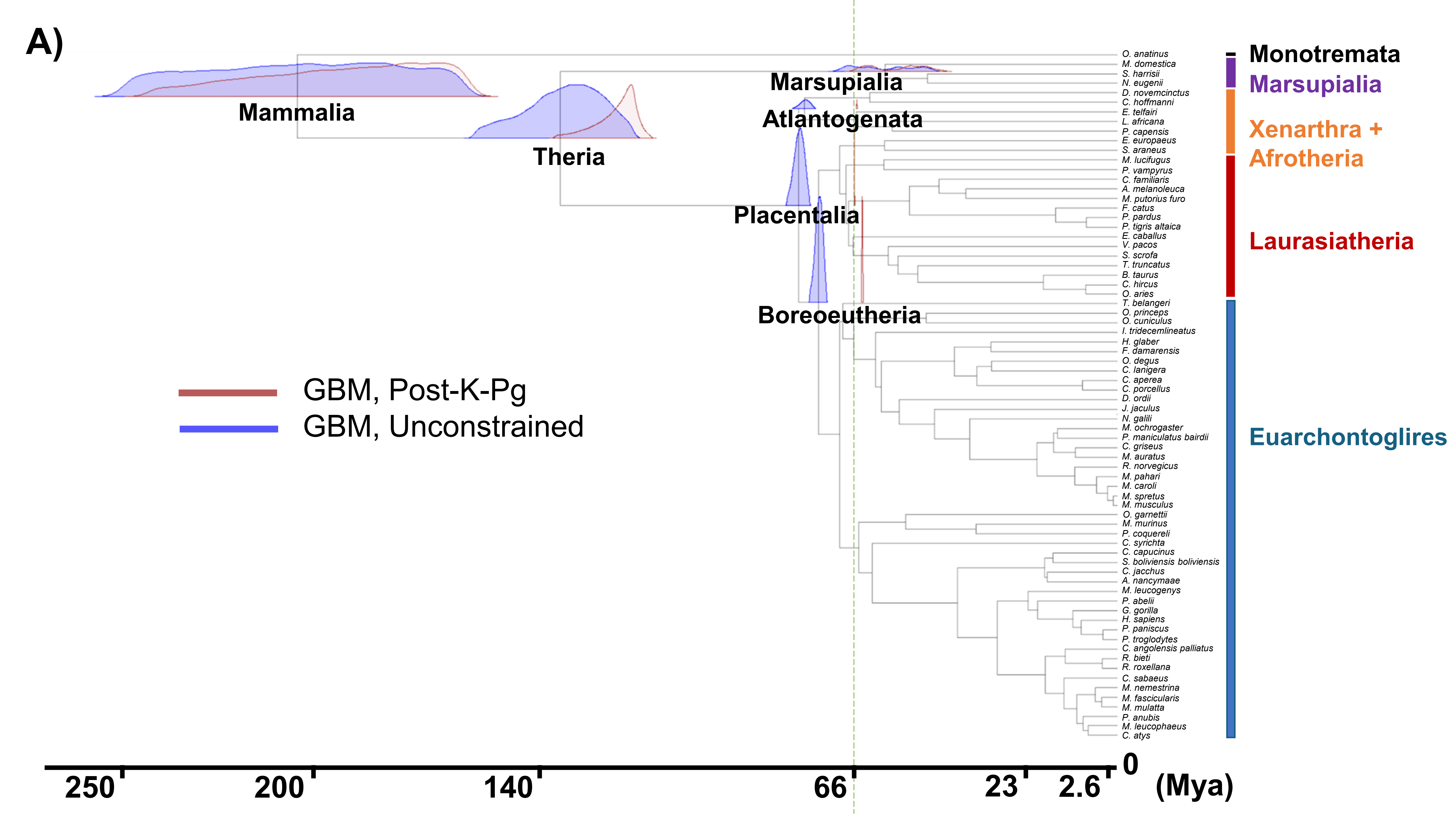

A)
Mammalia
Theria
Marsupialia
Atlantogenata
Placentalia
Boreoeutheria
GBM, Post-K-Pg
GBM, Unconstrained
O. anatinus
M. domestica
S. harrisii
N. eugenii
D. novemcinctus
C. hoffmanni
E. telfairi
L. africana
P. capensis
E. europaeus
S. araneus
M. lucifugus
P. vampyrus
C. familiaris
A. melanoleuca
M. putorius furo
F. catus
P. pardus
P. tigris altaica
E. caballus
V. pacos
S. scrofa
T. truncatus
B. taurus
C. hircus
O. aries
T. belangeri
O. princeps
O. cuniculus
I. tridecemlineatus
H. glaber
F. damarensis
O. degus
C. lanigera
C. aperea
C. porcellus
D. ordii
J. jaculus
N. galili
M. ochrogaster
P. maniculatus bairdii
C. griseus
M. auratus
R. norvegicus
M. pahari
M. caroli
M. spretus
M. musculus
O. garnettii
M. murinus
P. coquereli
C. syrichta
C. capucinus
S. boliviensis boliviensis
C. jacchus
A. nancymaae
M. leucogenys
P. abelii
G. gorilla
H. sapiens
P. paniscus
P. troglodytes
C. angolensis palliatus
R. bieti
R. roxellana
C. sabaeus
M. nemestrina
M. fascicularis
M. mulatta
P. anubis
M. leucophaeus
C. atys
Monotremata
Marsupialia
Xenarthra +
Afrotheria
Laurasiatheria
Euarchontoglires
250
200
140
66
23
2.6
0
(Mya)

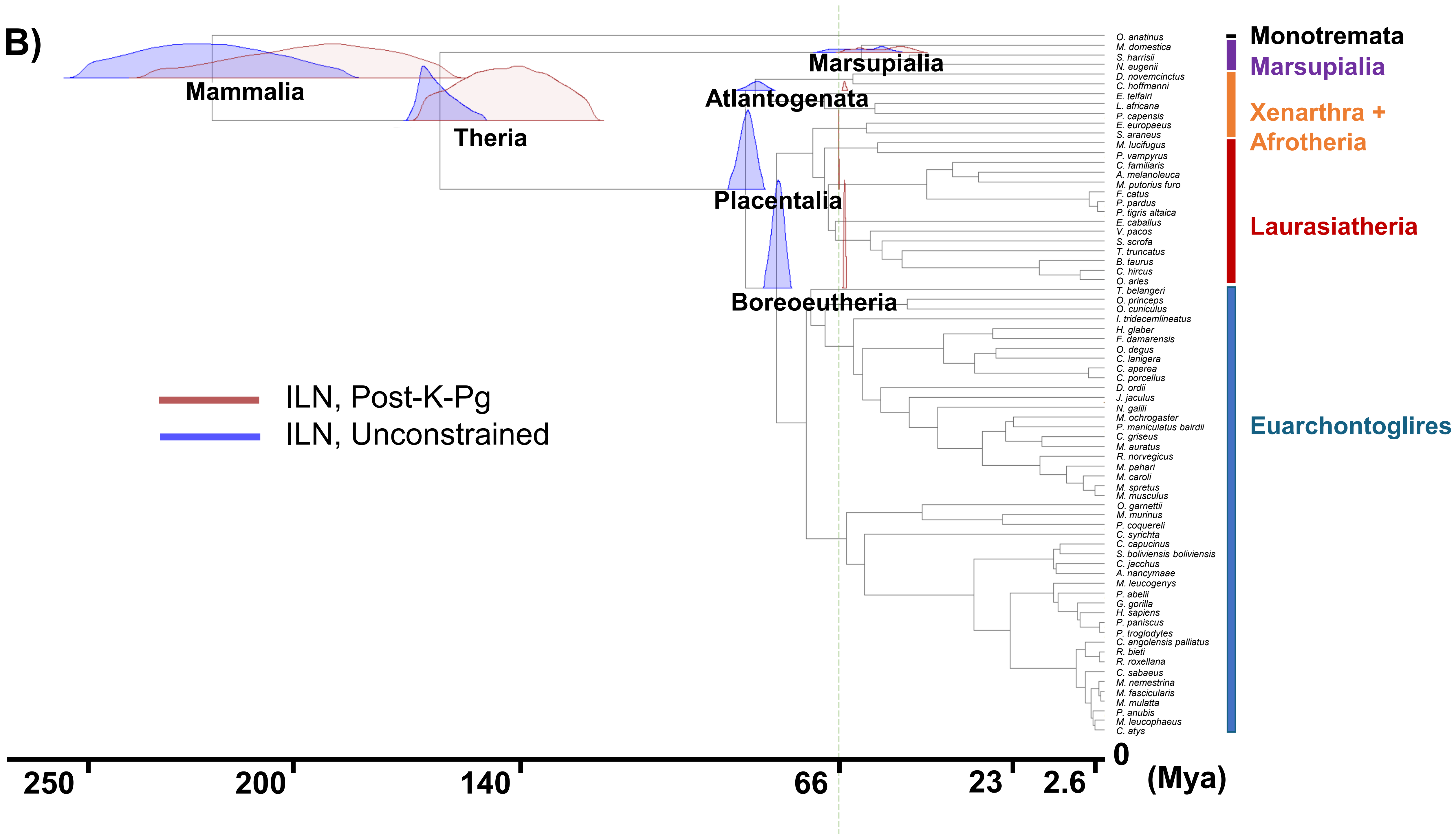

B)
Mammalia
Theria
Marsupialia
Atlantogenata
Placentalia
Boreoeutheria
ILN, Post-K-Pg
ILN, Unconstrained
O. anatinus
M. domestica
S. harrisii
N. eugenii
D. novemcinctus
C. hoffmanni
E. telfairi
L. africana
P. capensis
E. europaeus
S. araneus
M. lucifugus
P. vampyrus
C. familiaris
A. melanoleuca
M. putorius furo
F. catus
P. pardus
P. tigris altaica
E. caballus
V. pacos
S. scrofa
T. truncatus
B. taurus
C. hircus
O. aries
T. belangeri
O. princeps
O. cuniculus
I. tridecemlineatus
H. glaber
F. damarensis
O. degus
C. lanigera
C. aperea
C. porcellus
D. ordii
J. jaculus
N. galili
M. ochrogaster
P. maniculatus bairdii
C. griseus
M. auratus
R. norvegicus
M. pahari
M. caroli
M. spretus
M. musculus
O. garnettii
M. murinus
P. coquereli
C. syrichta
C. capucinus
S. boliviensis boliviensis
C. jacchus
A. nancymaae
M. leucogenys
P. abelii
G. gorilla
H. sapiens
P. paniscus
P. troglodytes
C. angolensis palliatus
R. bieti
R. roxellana
C. sabaeus
M. nemestrina
M. fascicularis
M. mulatta
P. anubis
M. leucophaeus
C. atys
Monotremata
Marsupialia
Xenarthra + Afrotheria
Laurasiatheria
Euarchontoglires
250
200
140
66
23
2.6
0
(Mya)
