# Supplementary material for: On the origin of animals and placental mammals: a critique of literalist readings of the fossil record
Source: Mol Biol Evol. 2026 Aug 19;43(9):msag211. doi: 10.1093/molbev/msag211 (PMC13543917; doi:10.1093/molbev/msag211)
Supplement: Supplementary file 1 [file msag211_supplementary_data.zip › Supp_Fig_S13_Animals_ConvPlotsPrior.pdf]

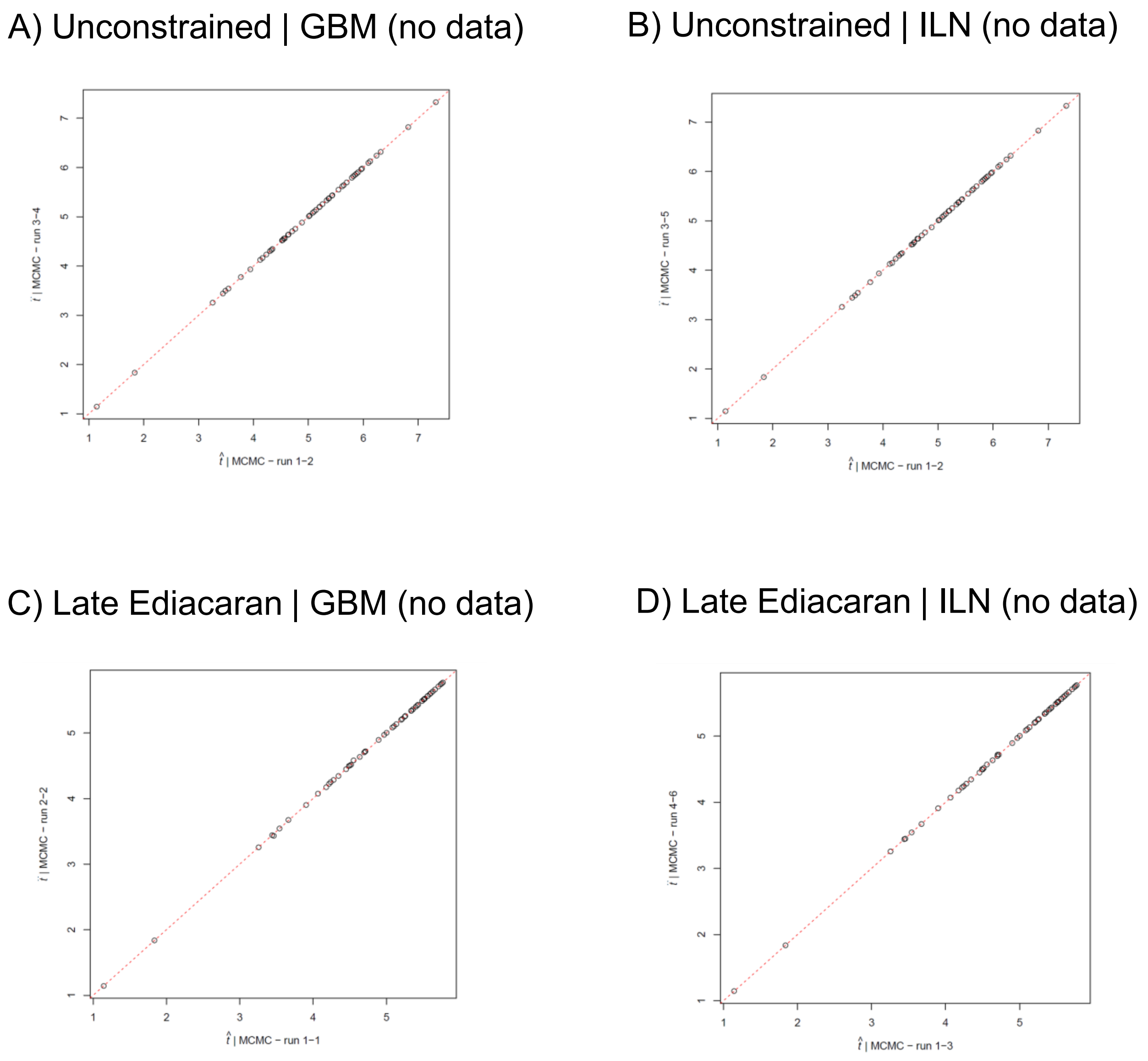
A) Unconstrained | GBM (no data)
B) Unconstrained | ILN (no data)
C) Late Ediacaran | GBM (no data)
D) Late Ediacaran | ILN (no data)
t̃ | MCMC – run 3–4
t̂ | MCMC – run 1–2
t̃ | MCMC – run 3–5
t̂ | MCMC – run 1–2
t̃ | MCMC – run 2–2
t̂ | MCMC – run 1–1
t̃ | MCMC – run 4–6
t̂ | MCMC – run 1–3
