# Supplementary material for: On the origin of animals and placental mammals: a critique of literalist readings of the fossil record
Source: Mol Biol Evol. 2026 Aug 19;43(9):msag211. doi: 10.1093/molbev/msag211 (PMC13543917; doi:10.1093/molbev/msag211)

A) Unconstrained | GBM (posterior)

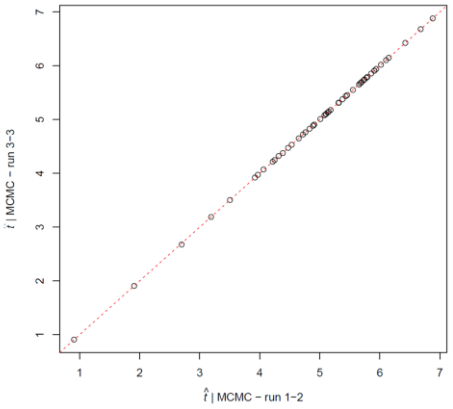

B) Unconstrained | ILN (posterior)

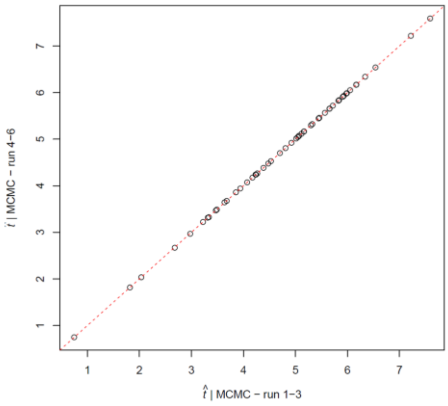

C) Late Ediacaran | GBM (posterior)

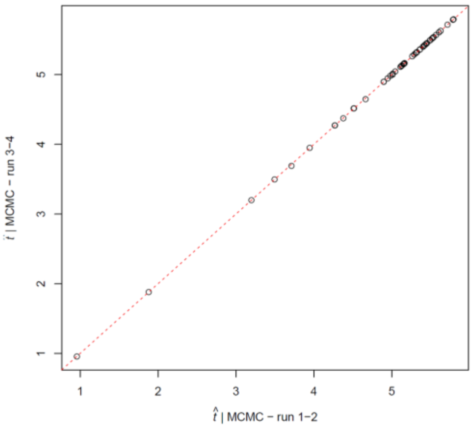

D) Late Ediacaran | ILN (posterior)

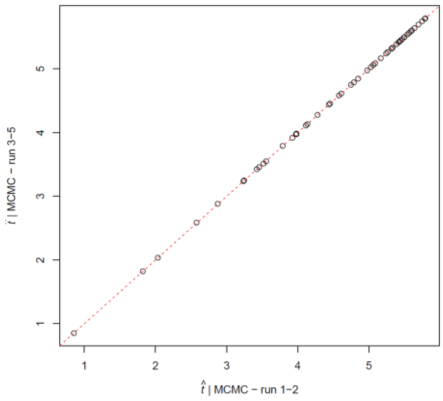

Supplement: Supplementary file 1 [file msag211_supplementary_data.zip › Supp_Fig_S14_AnimalsConvPlotsPost.pdf]
