# Supplementary material for: On the origin of animals and placental mammals: a critique of literalist readings of the fossil record
Source: Mol Biol Evol. 2026 Aug 19;43(9):msag211. doi: 10.1093/molbev/msag211 (PMC13543917; doi:10.1093/molbev/msag211)

S15) Unconstrained | NODAT | GBM

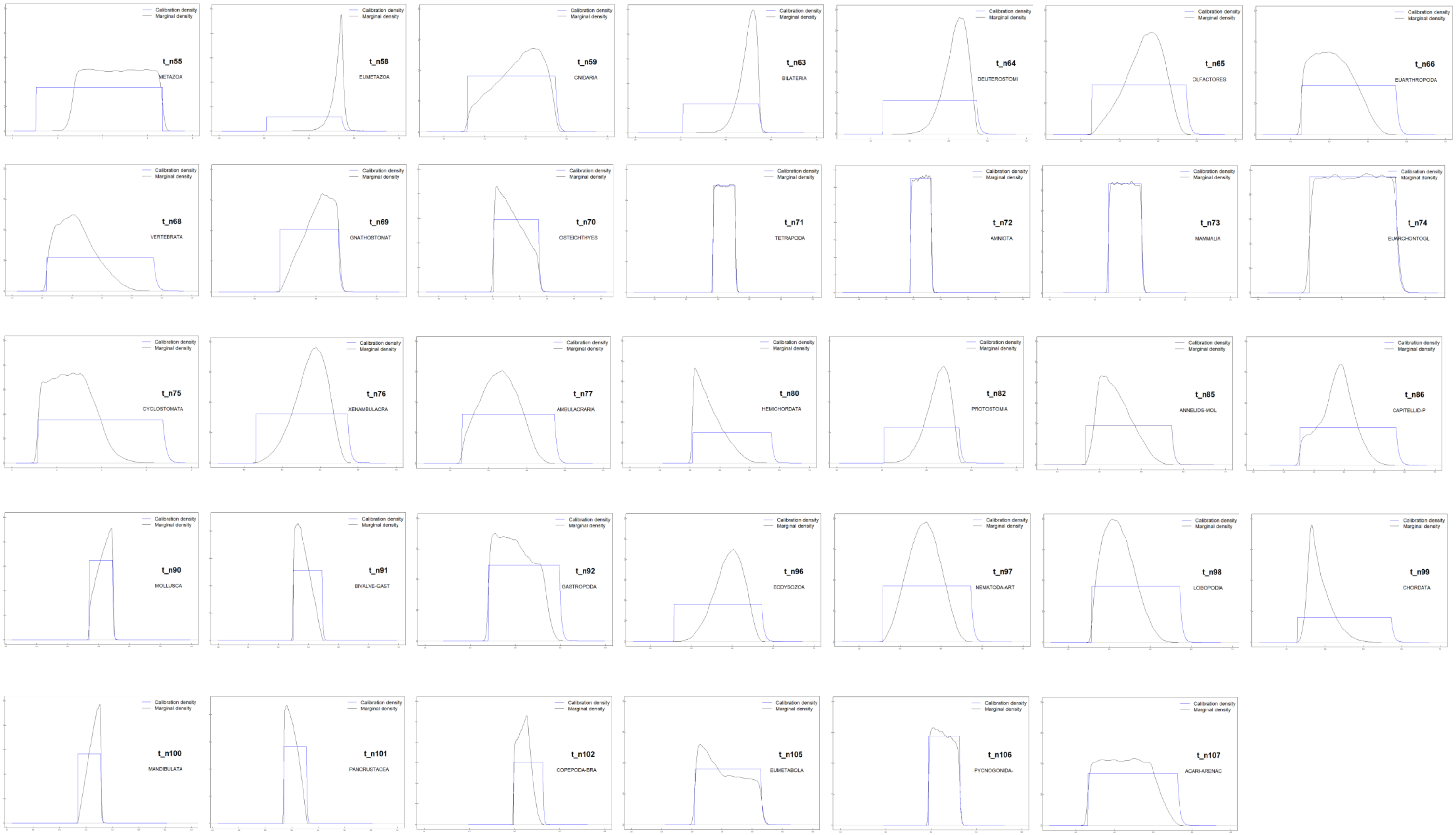

S16) Unconstrained | NODAT | ILN

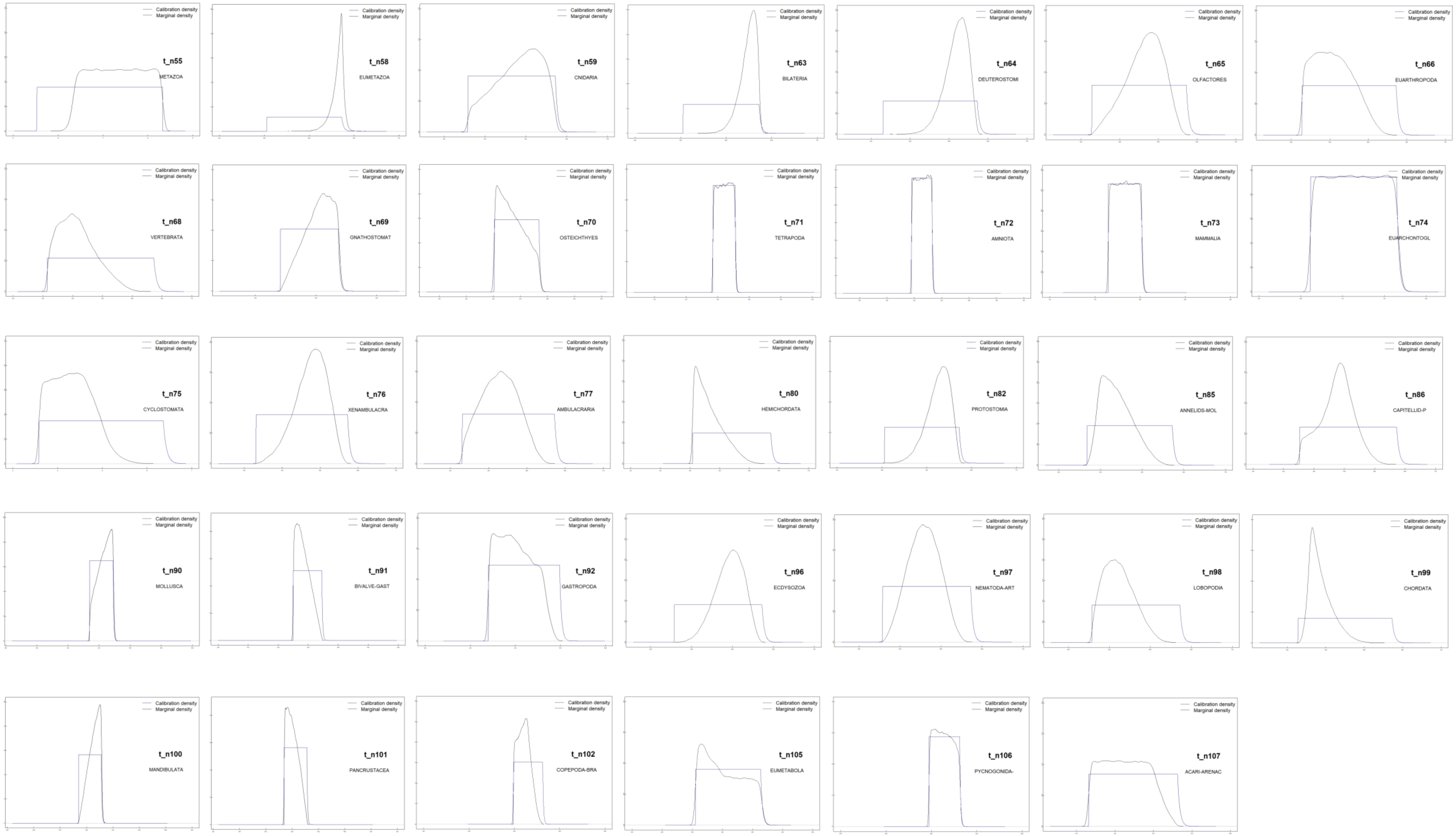

S17) Late Ediacaran | NODAT | GBM

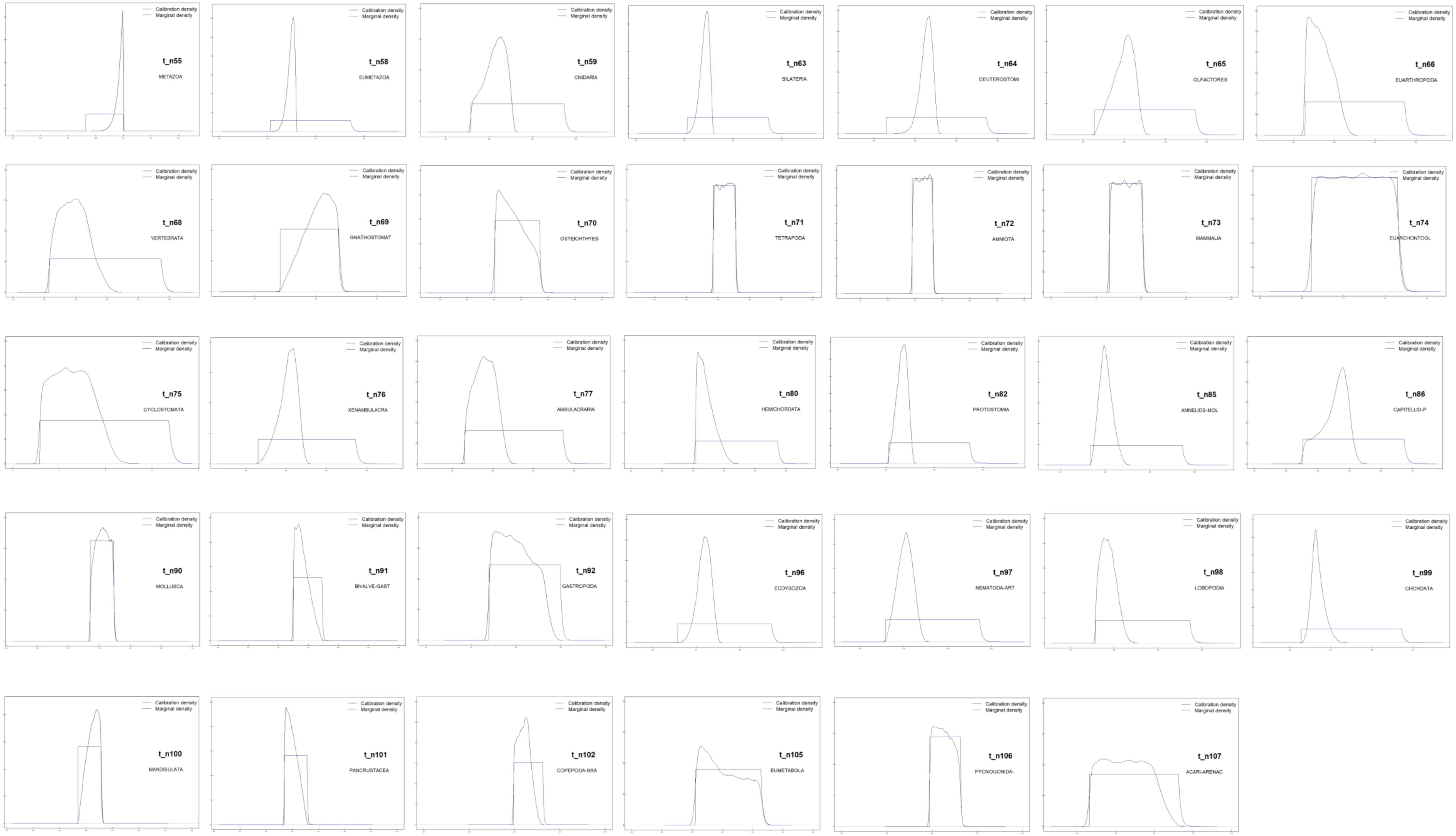

S18) Late Ediacaran | NODAT | ILN

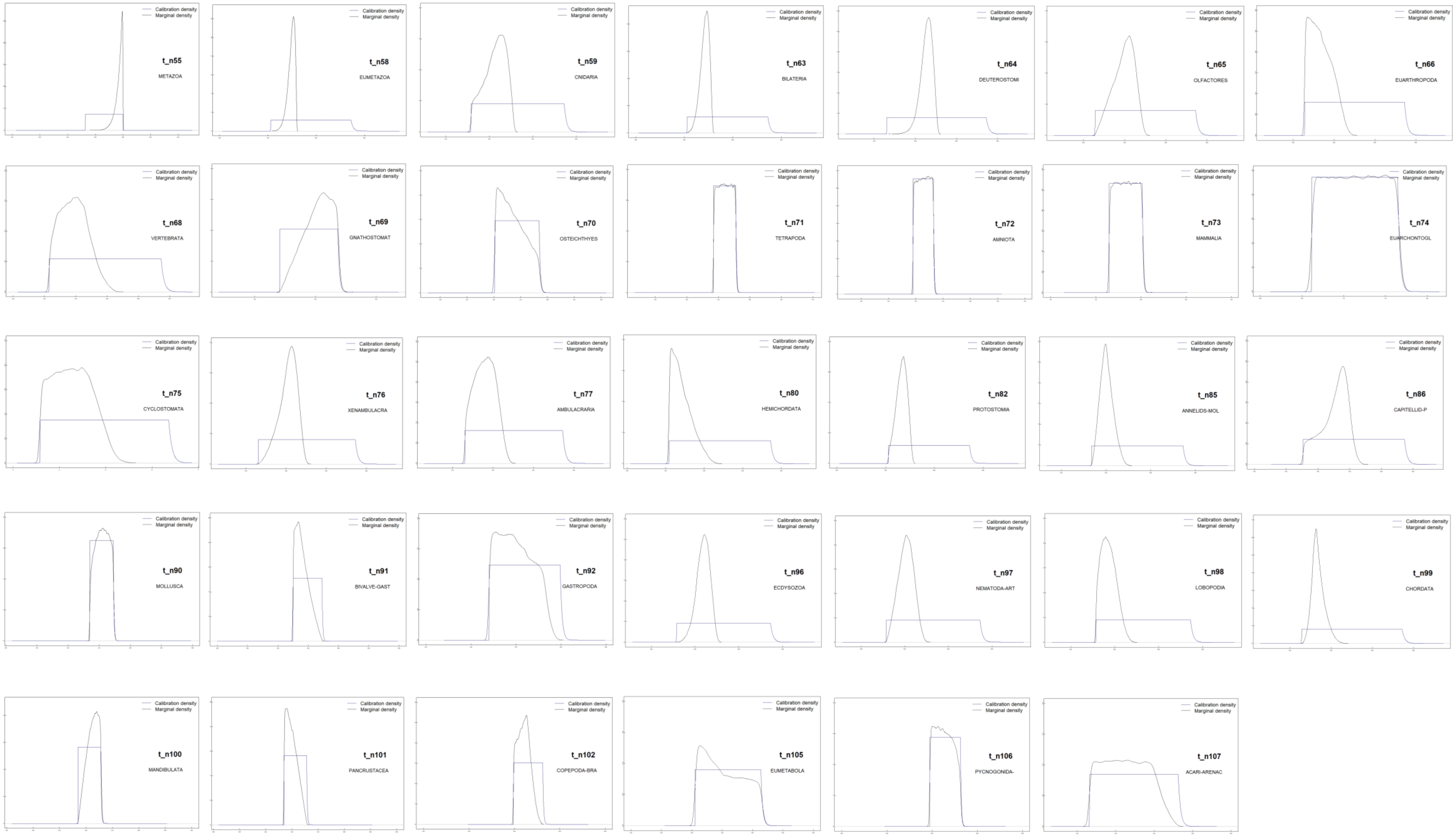

Supplement: Supplementary file 1 [file msag211_supplementary_data.zip › Supp_Fig_S15-S18_Animals_margVScalib.pdf]
