# Supplementary material for: On the origin of animals and placental mammals: a critique of literalist readings of the fossil record
Source: Mol Biol Evol. 2026 Aug 19;43(9):msag211. doi: 10.1093/molbev/msag211 (PMC13543917; doi:10.1093/molbev/msag211)
Supplement: Supplementary file 1 [file msag211_supplementary_data.zip › Supp_Fig_S19-S22_Animals_rategrams.pdf]

# S19) Unconstrained | GBM

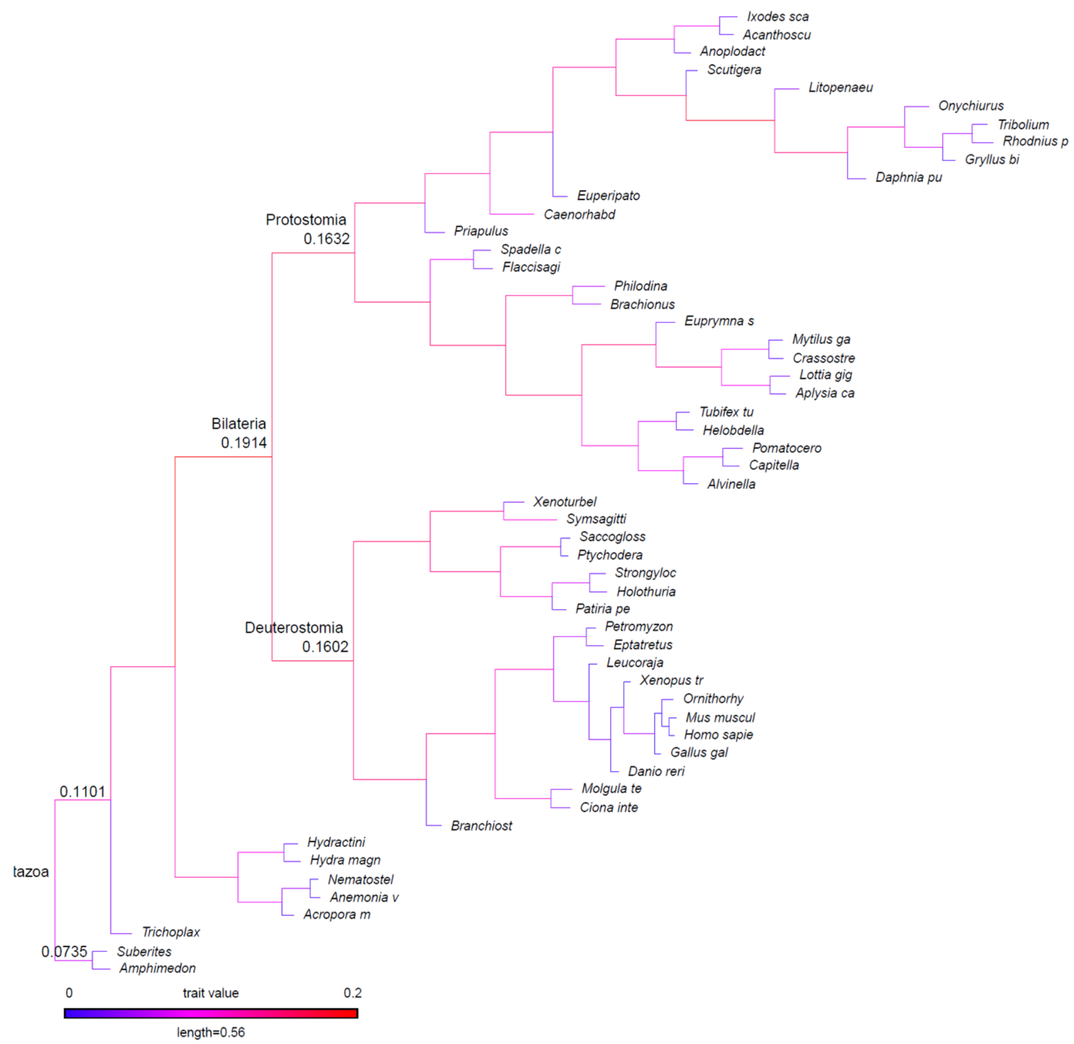


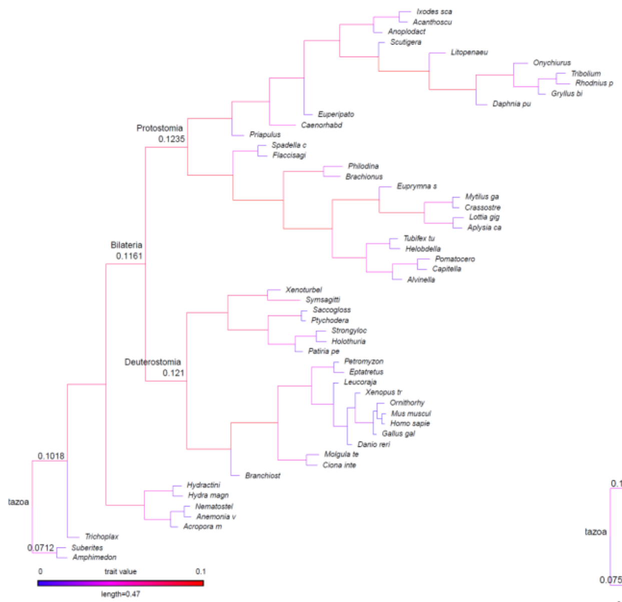

Partition 1

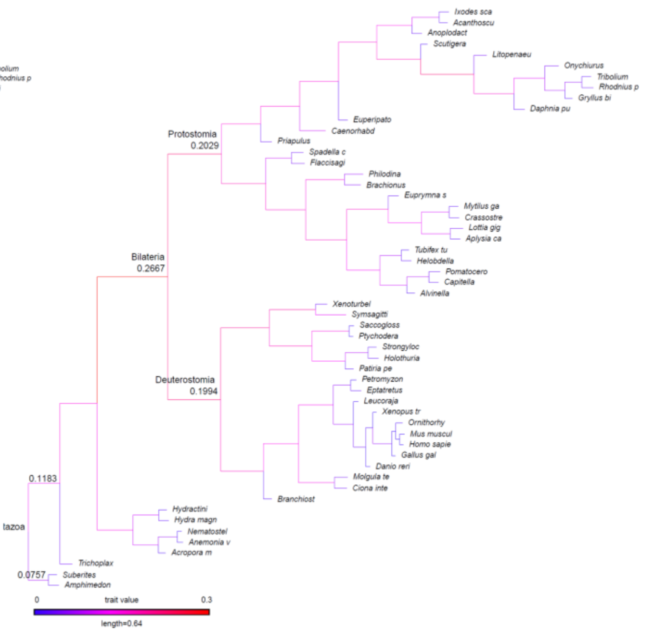

Partition 2

# S20) Unconstrained | ILN

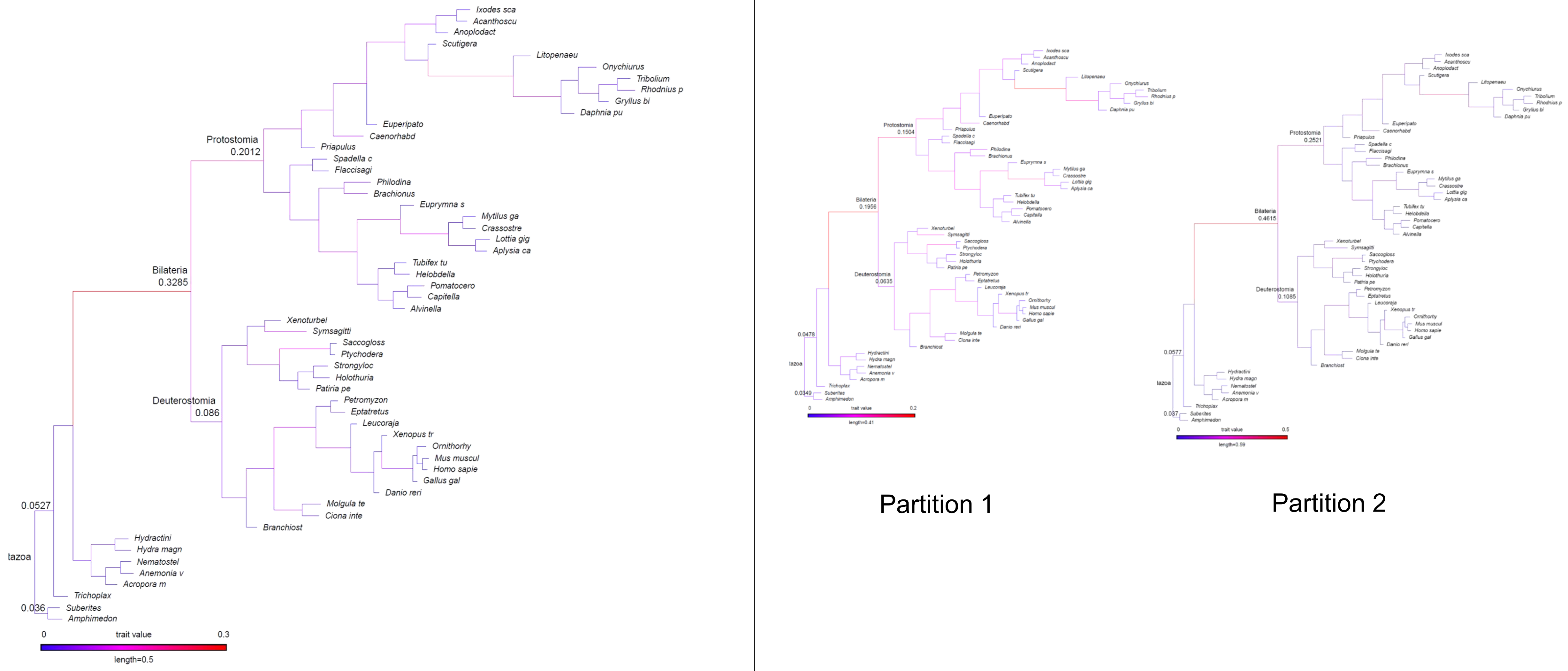


Partition 1

Partition 2

# S21) Late Ediacaran | GBM

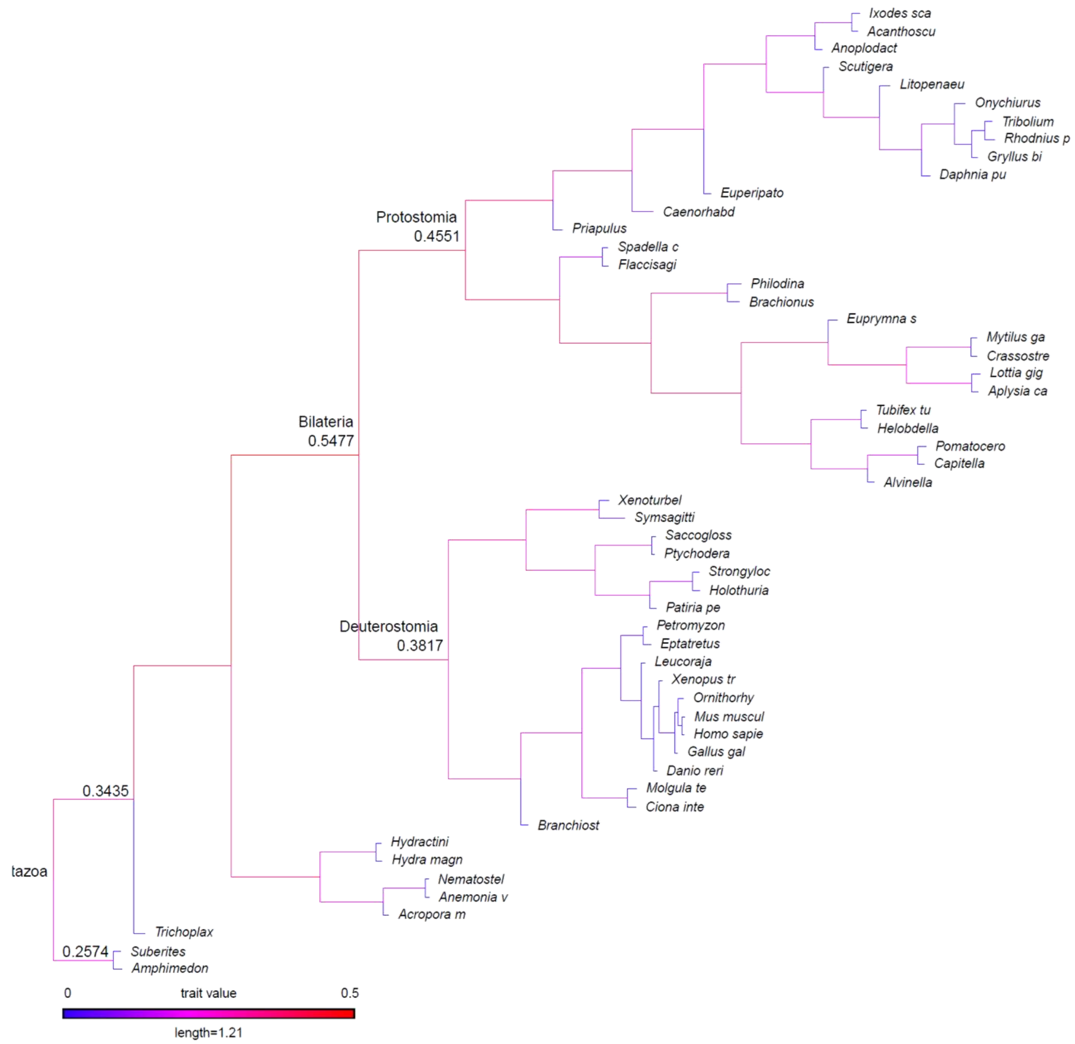


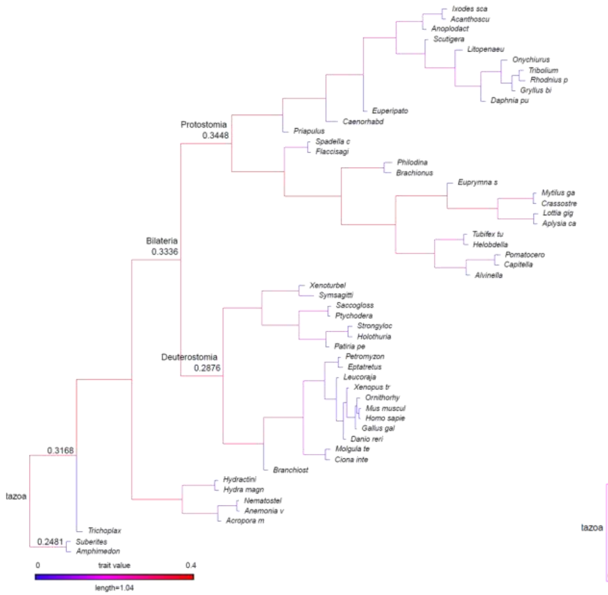


Partition 1

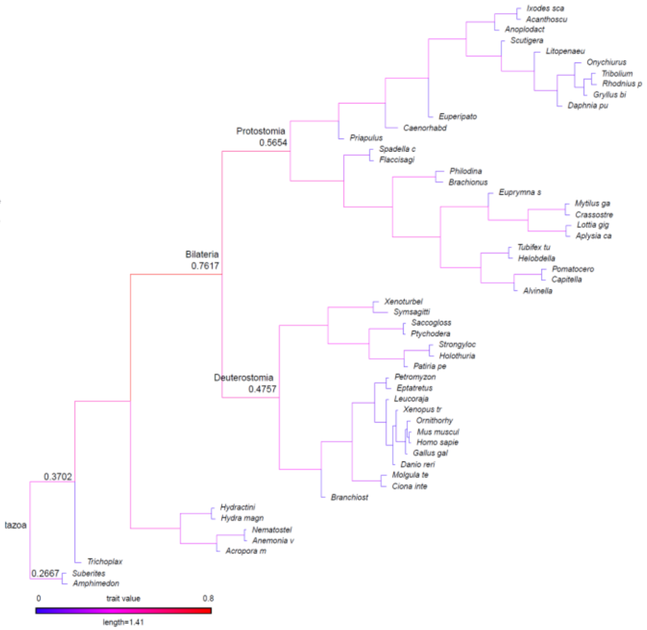


Partition 2

# S22) Late Ediacaran | ILN

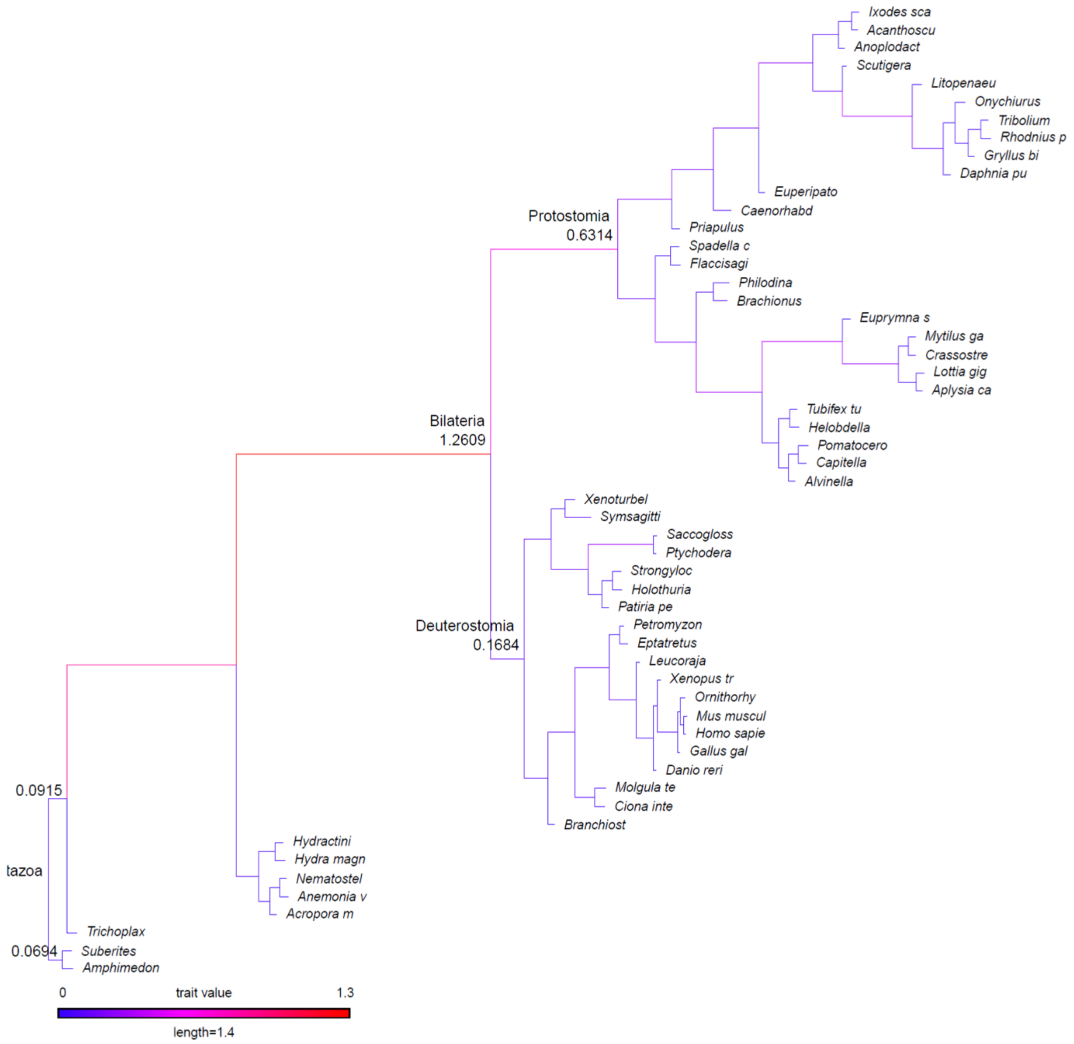


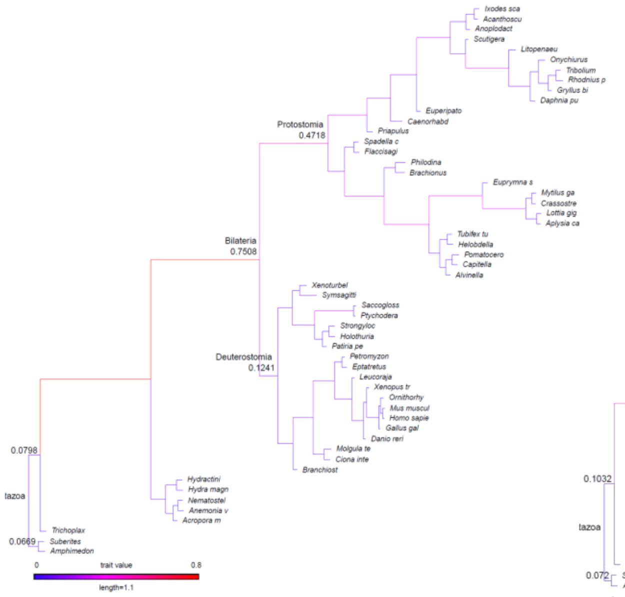


Partition 1

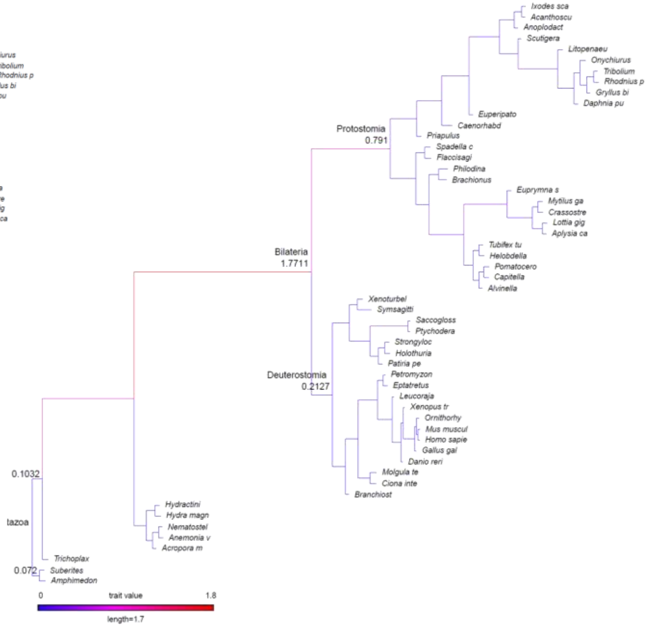


Partition 2
