# Supplementary material for: On the origin of animals and placental mammals: a critique of literalist readings of the fossil record
Source: Mol Biol Evol. 2026 Aug 19;43(9):msag211. doi: 10.1093/molbev/msag211 (PMC13543917; doi:10.1093/molbev/msag211)
Supplement: Supplementary file 1 [file msag211_supplementary_data.zip › Supp_Fig_S1_WorkflowAnalysis.pdf]

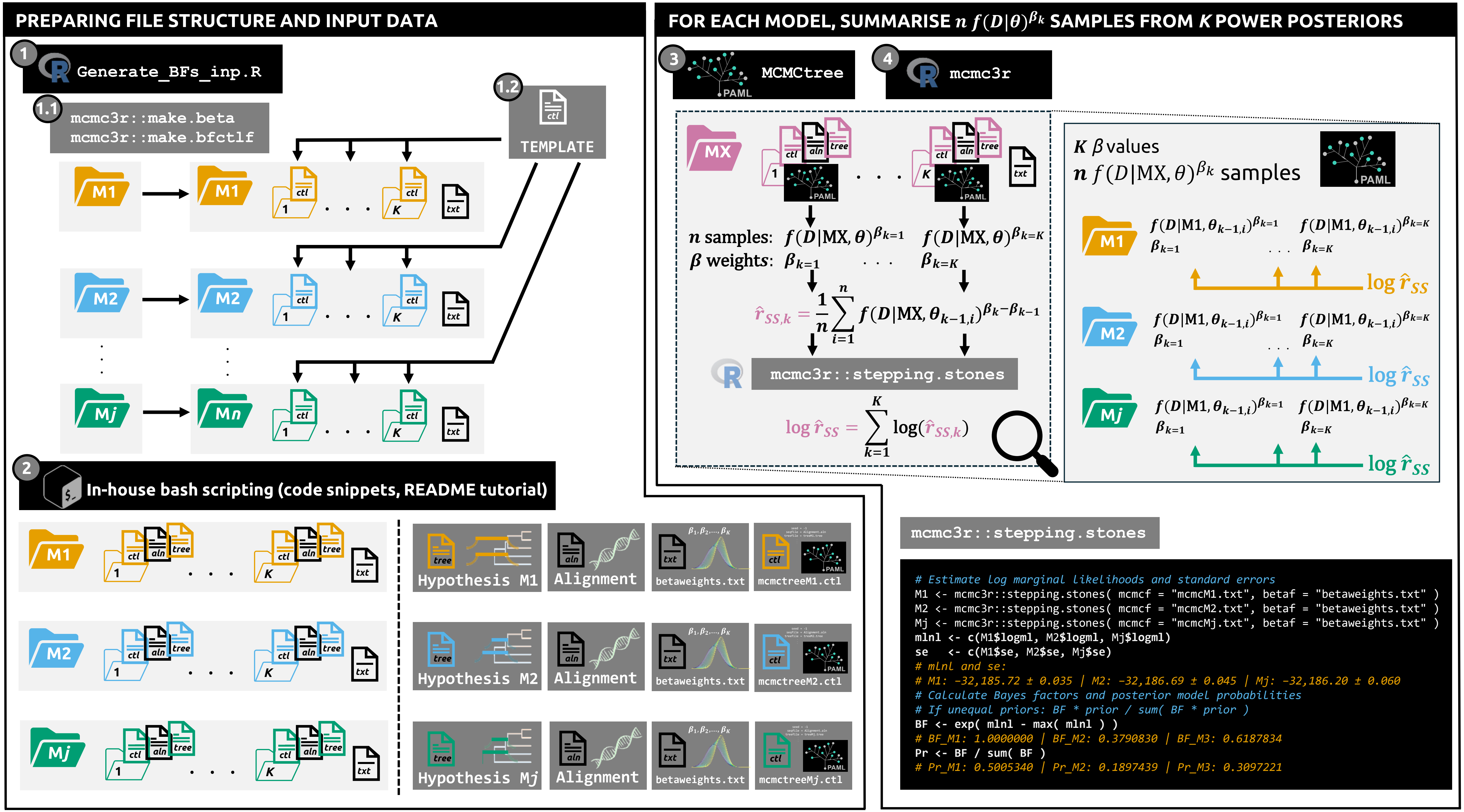
PREPARING FILE STRUCTURE AND INPUT DATA
1 Generate_BFs_inp.R
1.1 mcmc3r::make.beta
mcmc3r::make.bfctlf
1.2 ctl TEMPLATE
M1 M1 ctl 1 . . . ctl K txt
M2 M2 ctl 1 . . . ctl K txt
Mj Mn ctl 1 . . . ctl K txt
2 In-house bash scripting (code snippets, README tutorial)
M1 ctl aln tree 1 . . . ctl aln tree K txt
M2 ctl aln tree 1 . . . ctl aln tree K txt
Mj ctl aln tree 1 . . . ctl aln tree K txt
tree Hypothesis M1
aln Alignment
txt betaweights.txt
ctl PAML mcmctreeM1.ctl
tree Hypothesis M2
aln Alignment
txt betaweights.txt
ctl PAML mcmctreeM2.ctl
tree Hypothesis Mj
aln Alignment
txt betaweights.txt
ctl PAML mcmctreeMj.ctl
FOR EACH MODEL, SUMMARISE $n\ f(D|\theta)^{\beta_k}$ SAMPLES FROM K POWER POSTERIORS
3 PAML MCMCtree
4 mcmc3r
MX ctl aln tree 1 PAML . . . ctl aln tree K PAML txt
n samples: $f(D|\mathrm{MX},\theta)^{\beta_{k=1}}$ $f(D|\mathrm{MX},\theta)^{\beta_{k=K}}$
β weights: $\beta_{k=1}$ . . . $\beta_{k=K}$
$\hat{r}_{SS,k} = \frac{1}{n}\sum_{i=1}^{n} f(D|\mathrm{MX},\theta_{k-1,i})^{\beta_k-\beta_{k-1}}$
mcmc3r::stepping.stones
$\log \hat{r}_{SS} = \sum_{k=1}^{K} \log(\hat{r}_{SS,k})$
K β values
$n\ f(D|\mathrm{MX},\theta)^{\beta_k}$ samples
PAML
M1 $f(D|\mathrm{M1},\theta_{k-1,i})^{\beta_{k=1}}$ $\beta_{k=1}$ . . . $f(D|\mathrm{M1},\theta_{k-1,i})^{\beta_{k=K}}$ $\beta_{k=K}$ $\log \hat{r}_{SS}$
M2 $f(D|\mathrm{M1},\theta_{k-1,i})^{\beta_{k=1}}$ $\beta_{k=1}$ . . . $f(D|\mathrm{M1},\theta_{k-1,i})^{\beta_{k=K}}$ $\beta_{k=K}$ $\log \hat{r}_{SS}$
Mj $f(D|\mathrm{M1},\theta_{k-1,i})^{\beta_{k=1}}$ $\beta_{k=1}$ $f(D|\mathrm{M1},\theta_{k-1,i})^{\beta_{k=K}}$ $\beta_{k=K}$ $\log \hat{r}_{SS}$
mcmc3r::stepping.stones
# Estimate log marginal likelihoods and standard errors
M1 <- mcmc3r::stepping.stones(mcmcf = "mcmcM1.txt", betaf = "betaweights.txt")
M2 <- mcmc3r::stepping.stones(mcmcf = "mcmcM2.txt", betaf = "betaweights.txt")
Mj <- mcmc3r::stepping.stones(mcmcf = "mcmcMj.txt", betaf = "betaweights.txt")
mlnl <- c(M1$logml, M2$logml, Mj$logml)
se <- c(M1$se, M2$se, Mj$se)
# mlnl and se:
# M1: -32,185.72 ± 0.035 | M2: -32,186.69 ± 0.045 | Mj: -32,186.20 ± 0.060
# Calculate Bayes factors and posterior model probabilities
# If unequal priors: BF * prior / sum(BF * prior)
BF <- exp(mlnl - max(mlnl))
# BF_M1: 1.0000000 | BF_M2: 0.3790830 | BF_M3: 0.6187834
Pr <- BF / sum(BF)
# Pr_M1: 0.5005340 | Pr_M2: 0.1897439 | Pr_M3: 0.3097221
