# Supplementary material for: On the origin of animals and placental mammals: a critique of literalist readings of the fossil record
Source: Mol Biol Evol. 2026 Aug 19;43(9):msag211. doi: 10.1093/molbev/msag211 (PMC13543917; doi:10.1093/molbev/msag211)
Supplement: Supplementary file 1 [file msag211_supplementary_data.zip › Supp_Fig_S23_Animals_CompareDistsDivModelsPlots.pdf]

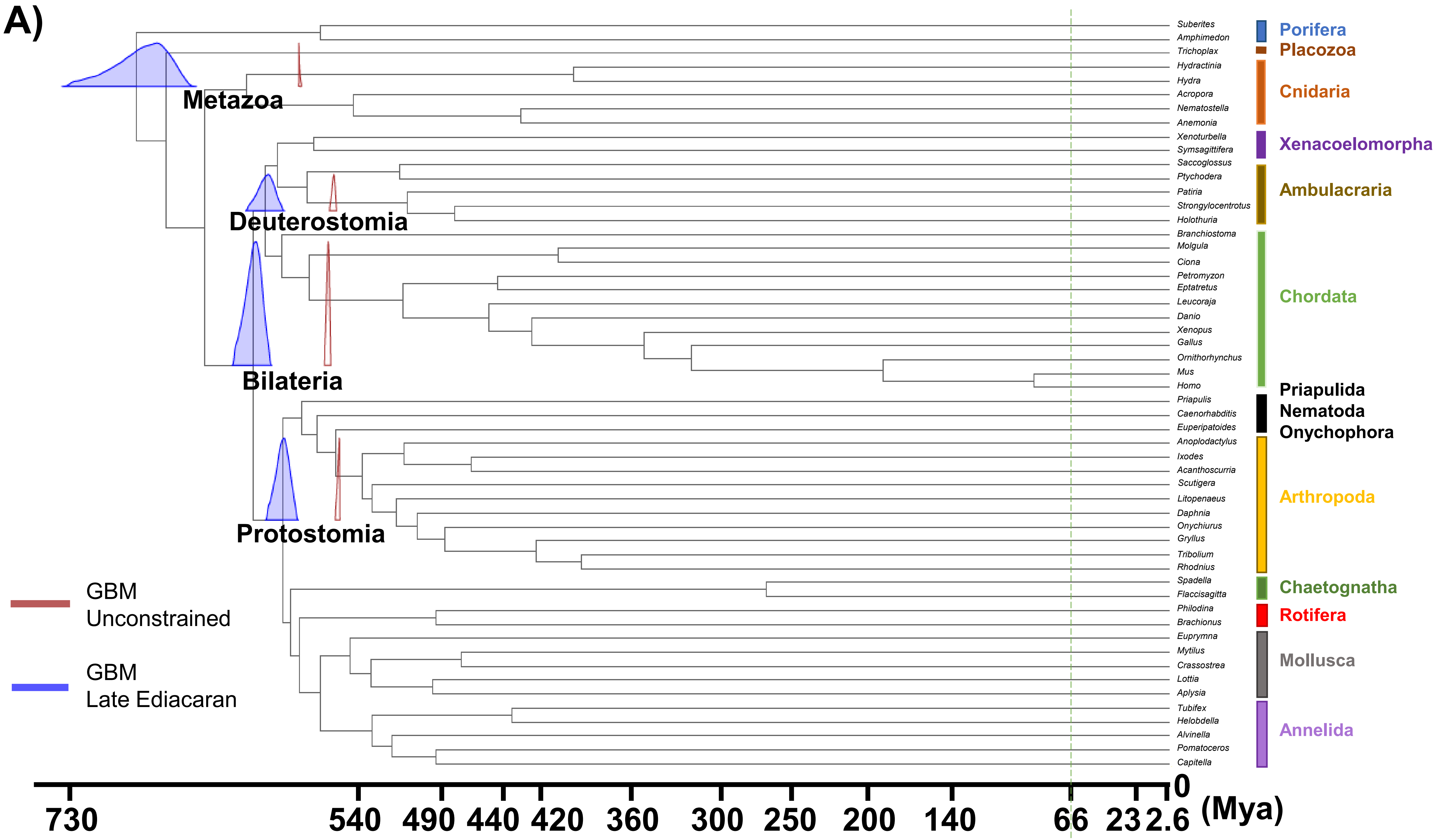

A)
Metazoa
Deuterostomia
Bilateria
Protostomia
GBM
Unconstrained
GBM
Late Ediacaran
Suberites
Amphimedon
Trichoplax
Hydractinia
Hydra
Acropora
Nematostella
Anemonia
Xenoturbella
Symsagittifera
Saccoglossus
Ptychodera
Patiria
Strongylocentrotus
Holothuria
Branchiostoma
Molgula
Ciona
Petromyzon
Eptatretus
Leucoraja
Danio
Xenopus
Gallus
Ornithorhynchus
Mus
Homo
Priapulis
Caenorhabditis
Euperipatoides
Anoplodactylus
Ixodes
Acanthoscurria
Scutigera
Litopenaeus
Daphnia
Onychiurus
Gryllus
Tribolium
Rhodnius
Spadella
Flaccisagitta
Philodina
Brachionus
Euprymna
Mytilus
Crassostrea
Lottia
Aplysia
Tubifex
Helobdella
Alvinella
Pomatoceros
Capitella
Porifera
Placozoa
Cnidaria
Xenacoelomorpha
Ambulacraria
Chordata
Priapulida
Nematoda
Onychophora
Arthropoda
Chaetognatha
Rotifera
Mollusca
Annelida
730
540
490
440
420
360
300
250
200
140
66
23
2.6
0
(Mya)

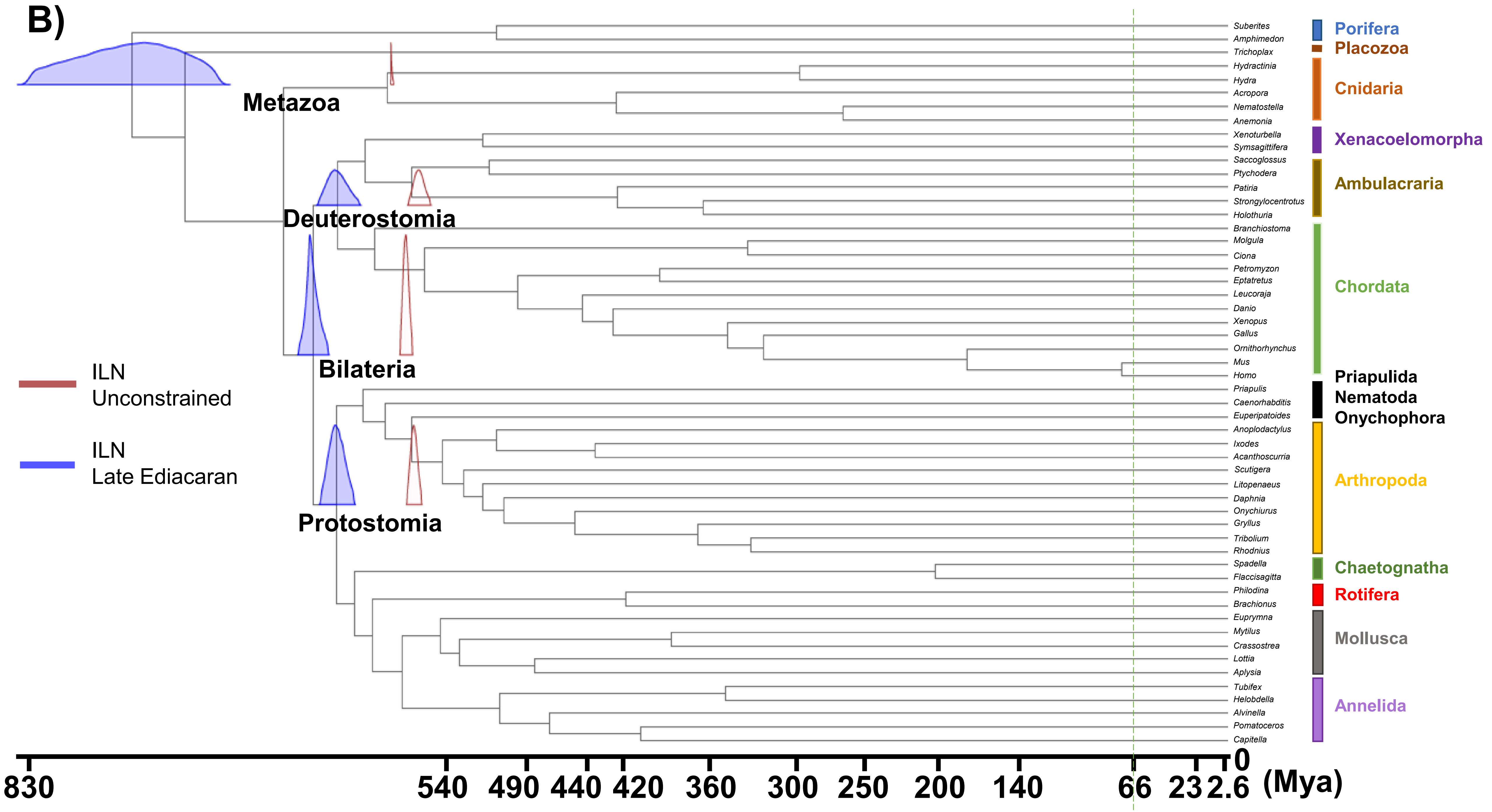
B)
Metazoa
Deuterostomia
Bilateria
Protostomia
ILN
Unconstrained
ILN
Late Ediacaran
Suberites
Amphimedon
Trichoplax
Hydractinia
Hydra
Acropora
Nematostella
Anemonia
Xenoturbella
Symsagittifera
Saccoglossus
Ptychodera
Patiria
Strongylocentrotus
Holothuria
Branchiostoma
Molgula
Ciona
Petromyzon
Eptatretus
Leucoraja
Danio
Xenopus
Gallus
Ornithorhynchus
Mus
Homo
Priapulis
Caenorhabditis
Euperipatoides
Anoplodactylus
Ixodes
Acanthoscurria
Scutigera
Litopenaeus
Daphnia
Onychiurus
Gryllus
Tribolium
Rhodnius
Spadella
Flaccisagitta
Philodina
Brachionus
Euprymna
Mytilus
Crassostrea
Lottia
Aplysia
Tubifex
Helobdella
Alvinella
Pomatoceros
Capitella
Porifera
Placozoa
Cnidaria
Xenacoelomorpha
Ambulacraria
Chordata
Priapulida
Nematoda
Onychophora
Arthropoda
Chaetognatha
Rotifera
Mollusca
Annelida
830
540
490
440
420
360
300
250
200
140
66
23
2.6
0
(Mya)
