# Supplementary material for: On the origin of animals and placental mammals: a critique of literalist readings of the fossil record
Source: Mol Biol Evol. 2026 Aug 19;43(9):msag211. doi: 10.1093/molbev/msag211 (PMC13543917; doi:10.1093/molbev/msag211)
Supplement: Supplementary file 1 [file msag211_supplementary_data.zip › Supp_Fig_S24_OrderStatisticsAndTruncation.pdf]

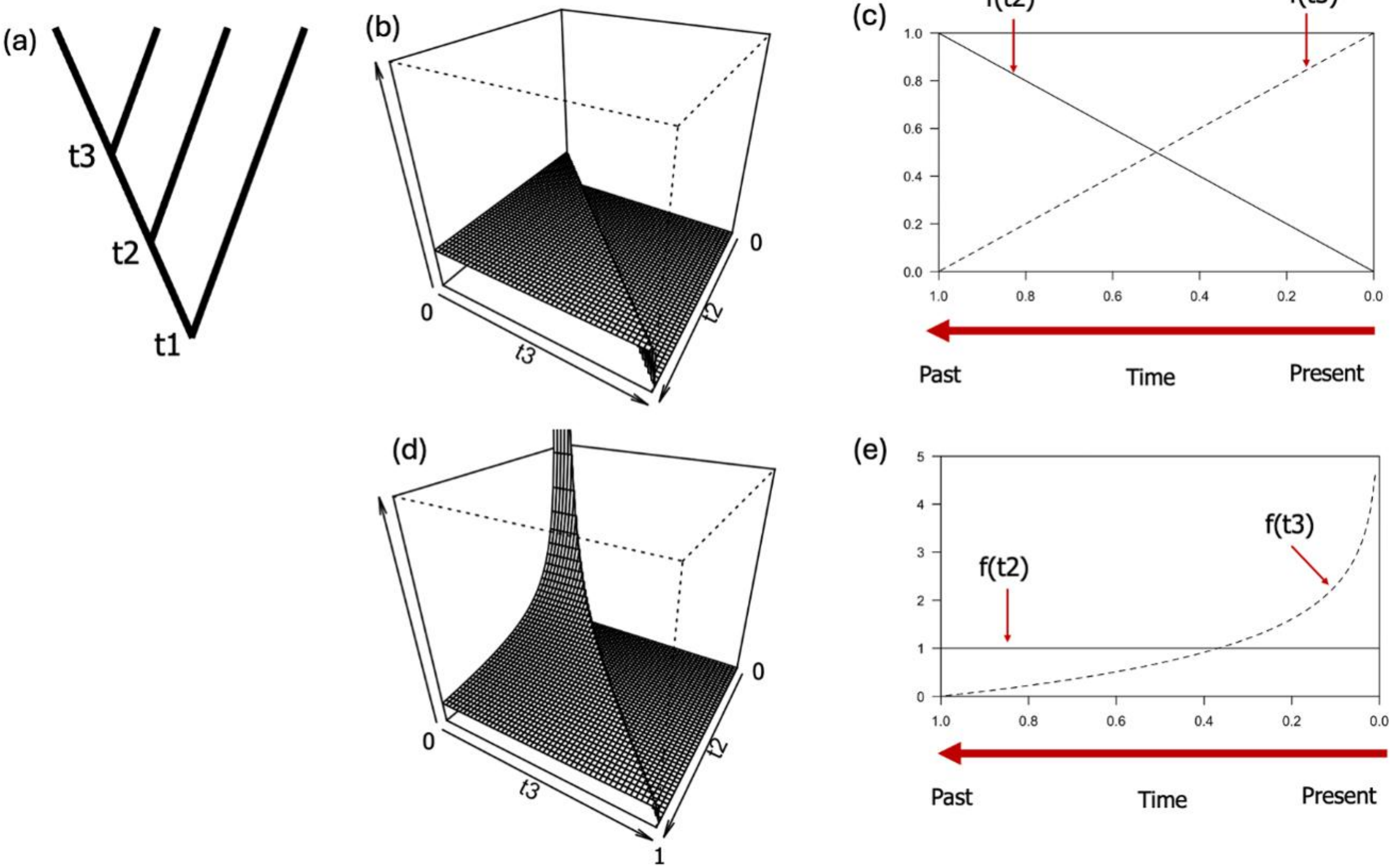

(a)
t3
t2
t1
(b)
0
t3
t2
0
(c)
f(t2)
f(t3)
1.0
0.8
0.6
0.4
0.2
0.0
1.0
0.8
0.6
0.4
0.2
0.0
Past
Time
Present
(d)
0
t3
t2
0
1
(e)
5
4
3
2
1
0
f(t3)
f(t2)
1.0
0.8
0.6
0.4
0.2
0.0
Past
Time
Present
