# Supplementary material for: On the origin of animals and placental mammals: a critique of literalist readings of the fossil record
Source: Mol Biol Evol. 2026 Aug 19;43(9):msag211. doi: 10.1093/molbev/msag211 (PMC13543917; doi:10.1093/molbev/msag211)

A) Post-K-Pg | GBM (no data)

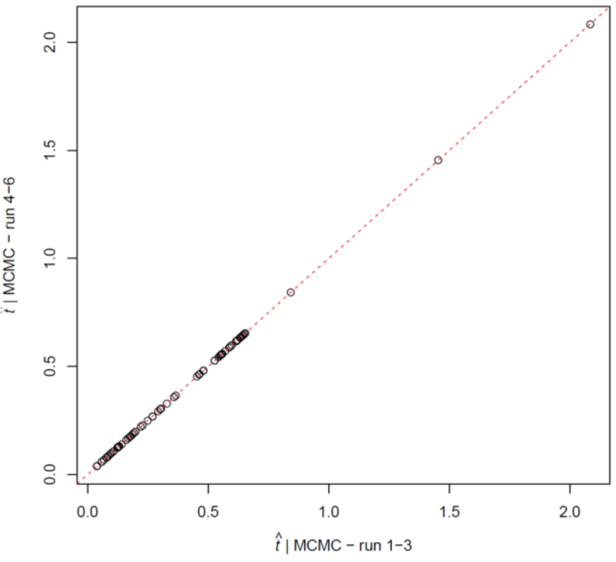

B) Post-K-Pg | ILN (no data)

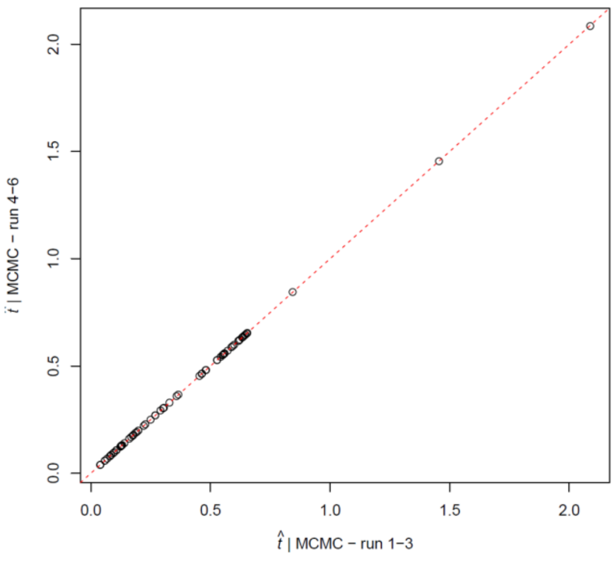

C) Unconstrained | GBM (no data)

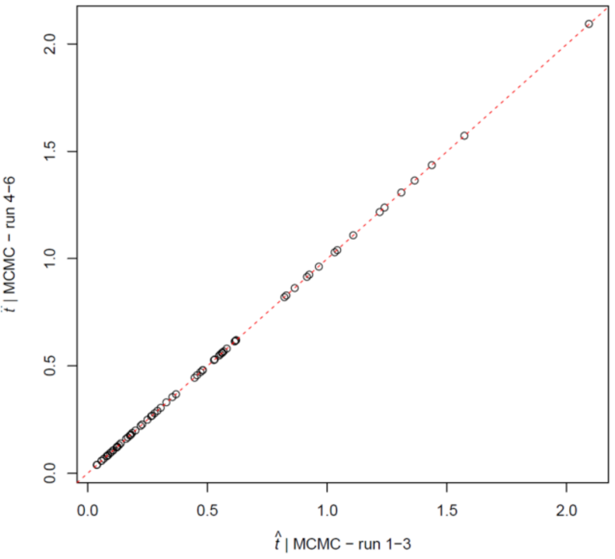

D) Unconstrained | ILN (no data)

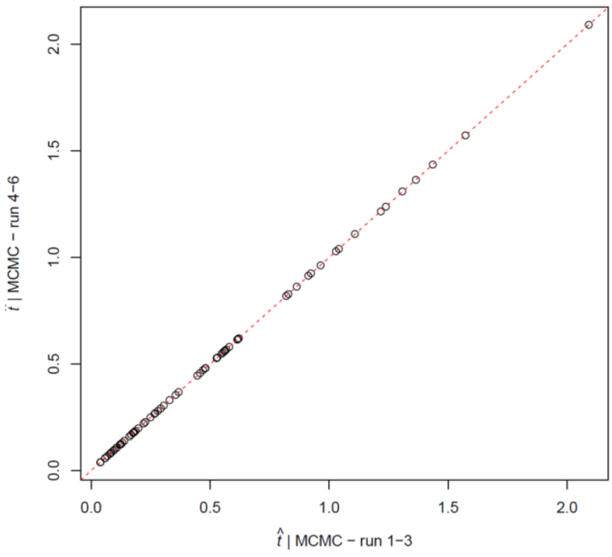

Supplement: Supplementary file 1 [file msag211_supplementary_data.zip › Supp_Fig_S2_Mammals_ConvPlotsPrior.pdf]
