# Supplementary material for: On the origin of animals and placental mammals: a critique of literalist readings of the fossil record
Source: Mol Biol Evol. 2026 Aug 19;43(9):msag211. doi: 10.1093/molbev/msag211 (PMC13543917; doi:10.1093/molbev/msag211)
Supplement: Supplementary file 1 [file msag211_supplementary_data.zip › Supp_Fig_S3_Mammals_ConvPlotsPost.pdf]

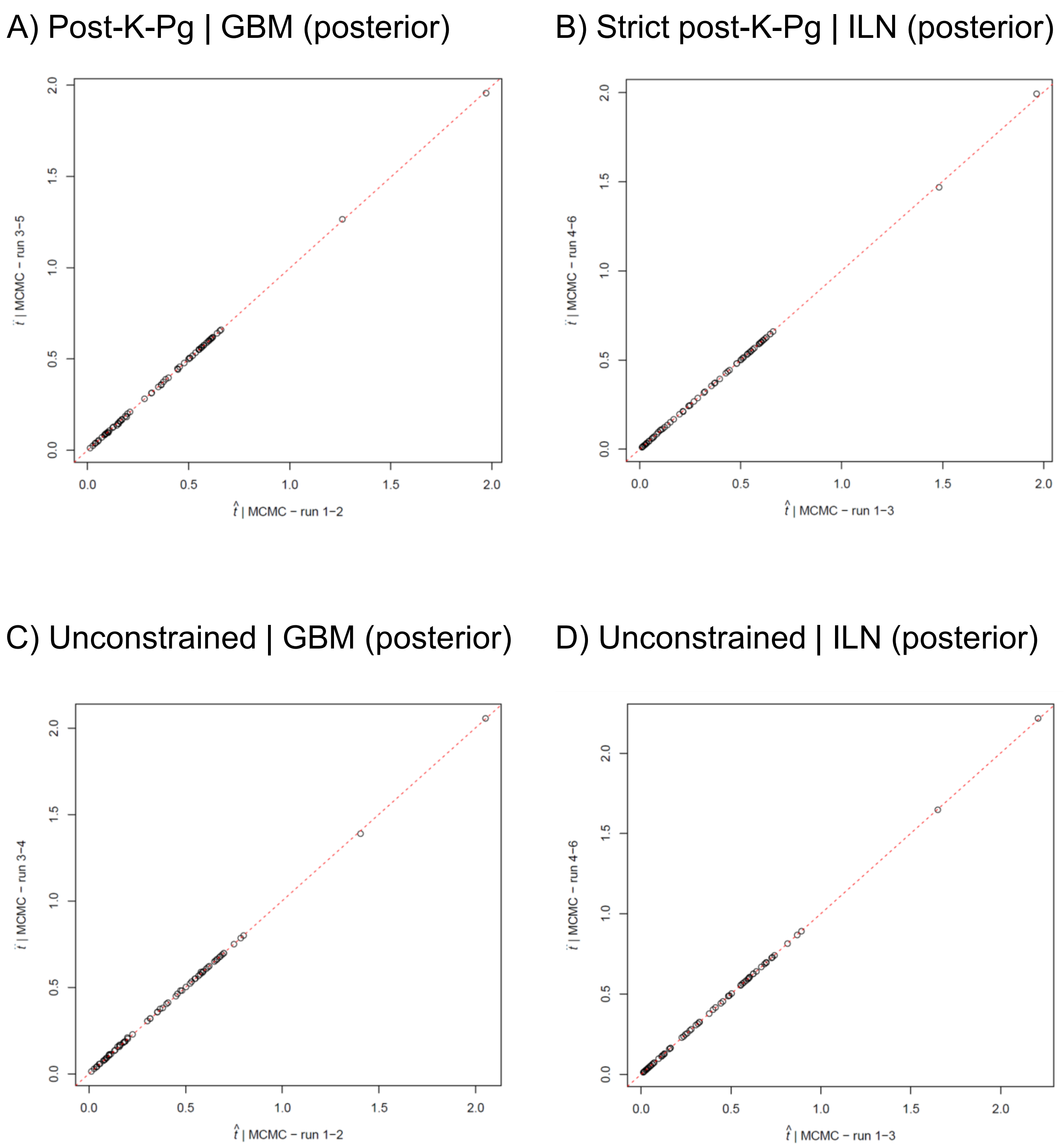

A) Post-K-Pg | GBM (posterior)
B) Strict post-K-Pg | ILN (posterior)
C) Unconstrained | GBM (posterior)
D) Unconstrained | ILN (posterior)
$\hat{t}$ | MCMC – run 1–2
$\hat{t}$ | MCMC – run 3–5
$\hat{t}$ | MCMC – run 1–3
$\hat{t}$ | MCMC – run 4–6
$\hat{t}$ | MCMC – run 1–2
$\hat{t}$ | MCMC – run 3–4
$\hat{t}$ | MCMC – run 1–3
$\hat{t}$ | MCMC – run 4–6
0.0
0.5
1.0
1.5
2.0
