# Supplementary material for: On the origin of animals and placental mammals: a critique of literalist readings of the fossil record
Source: Mol Biol Evol. 2026 Aug 19;43(9):msag211. doi: 10.1093/molbev/msag211 (PMC13543917; doi:10.1093/molbev/msag211)
Supplement: Supplementary file 1 [file msag211_supplementary_data.zip › Supp_Fig_S4-S7_Mammals_margVScalib.pdf]

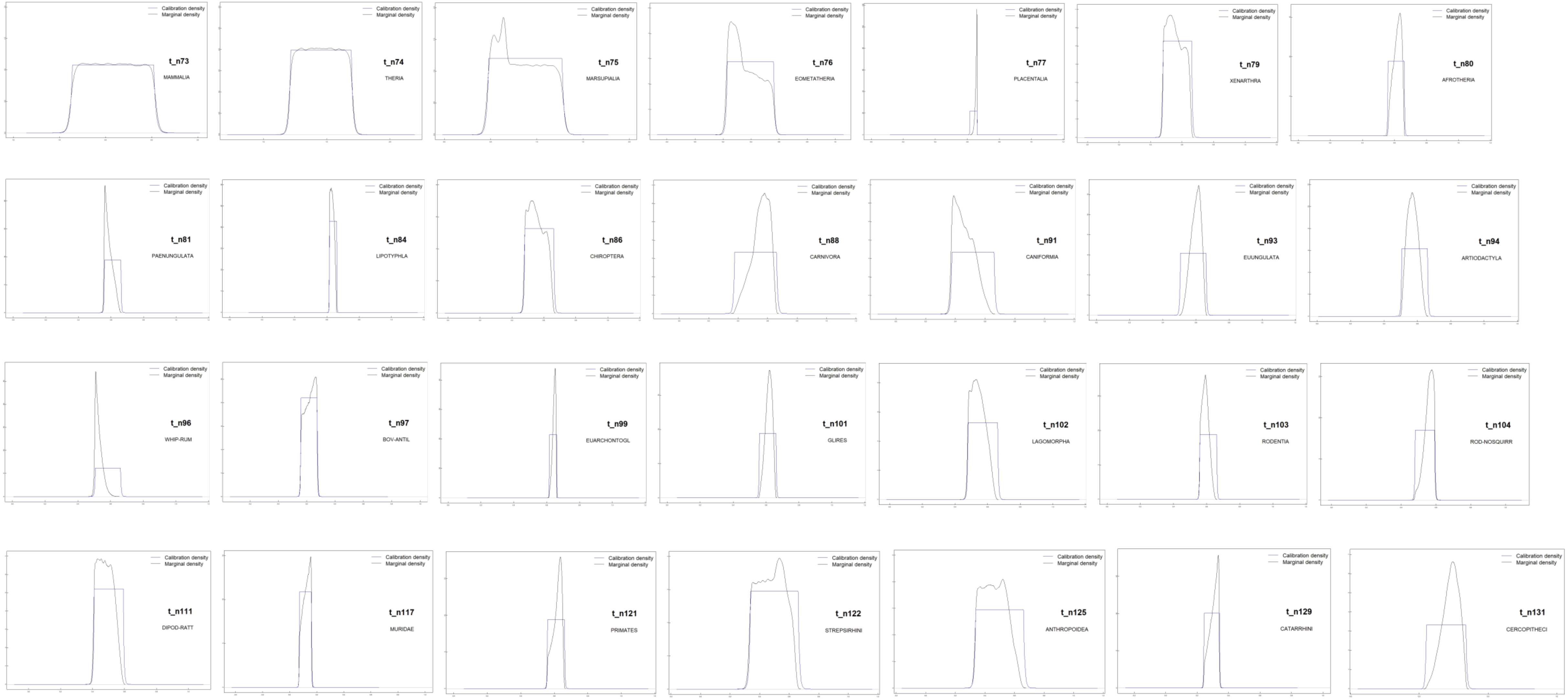

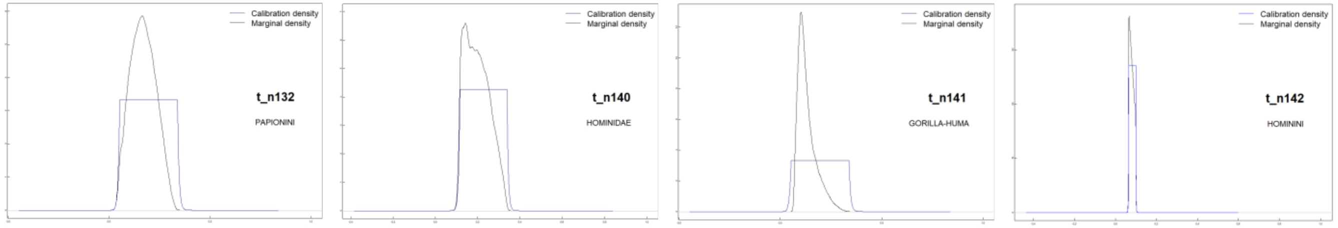

S4) Post-K-Pg | NODAT | GBM

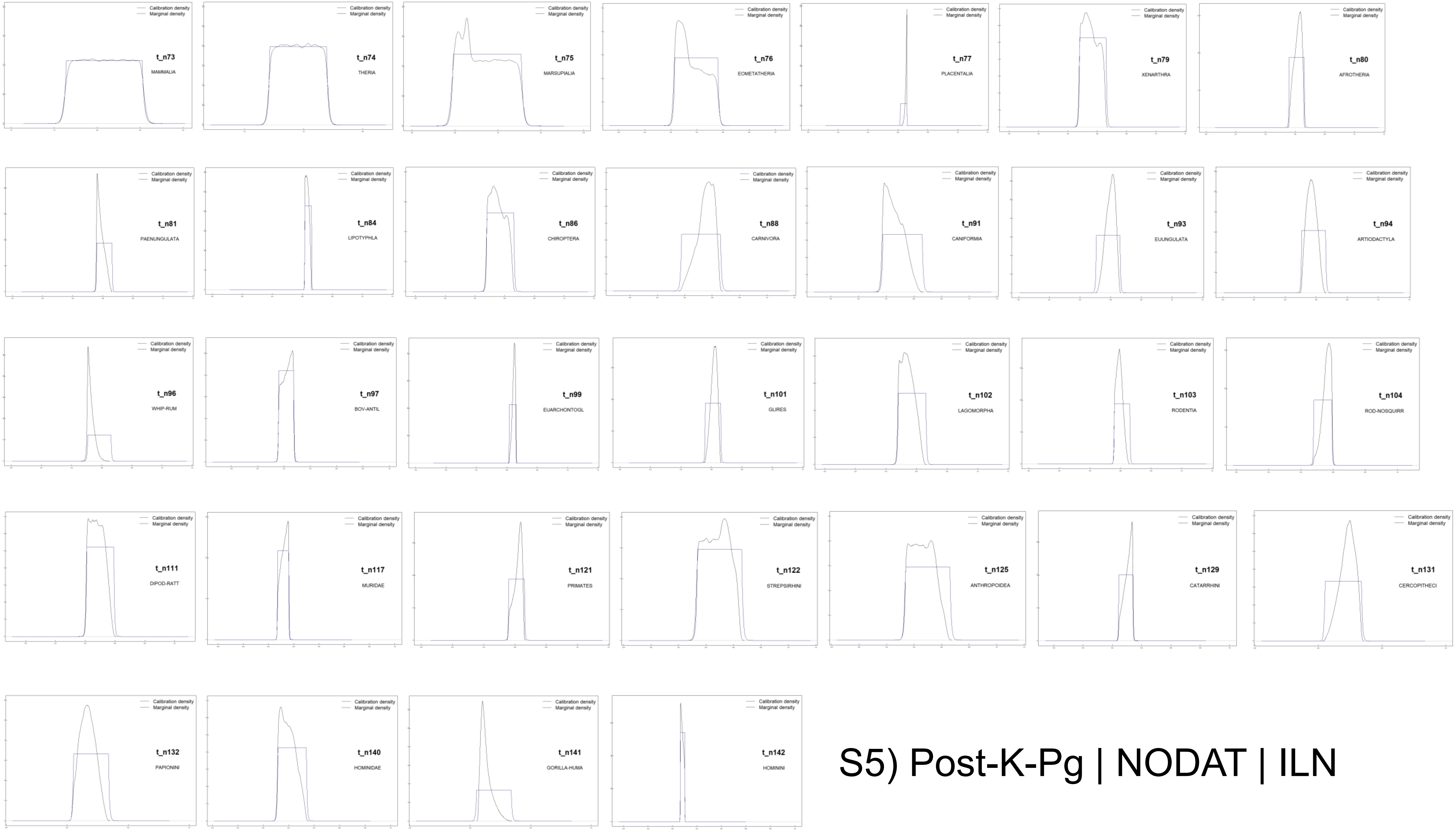

S5) Post-K-Pg | NODAT | ILN

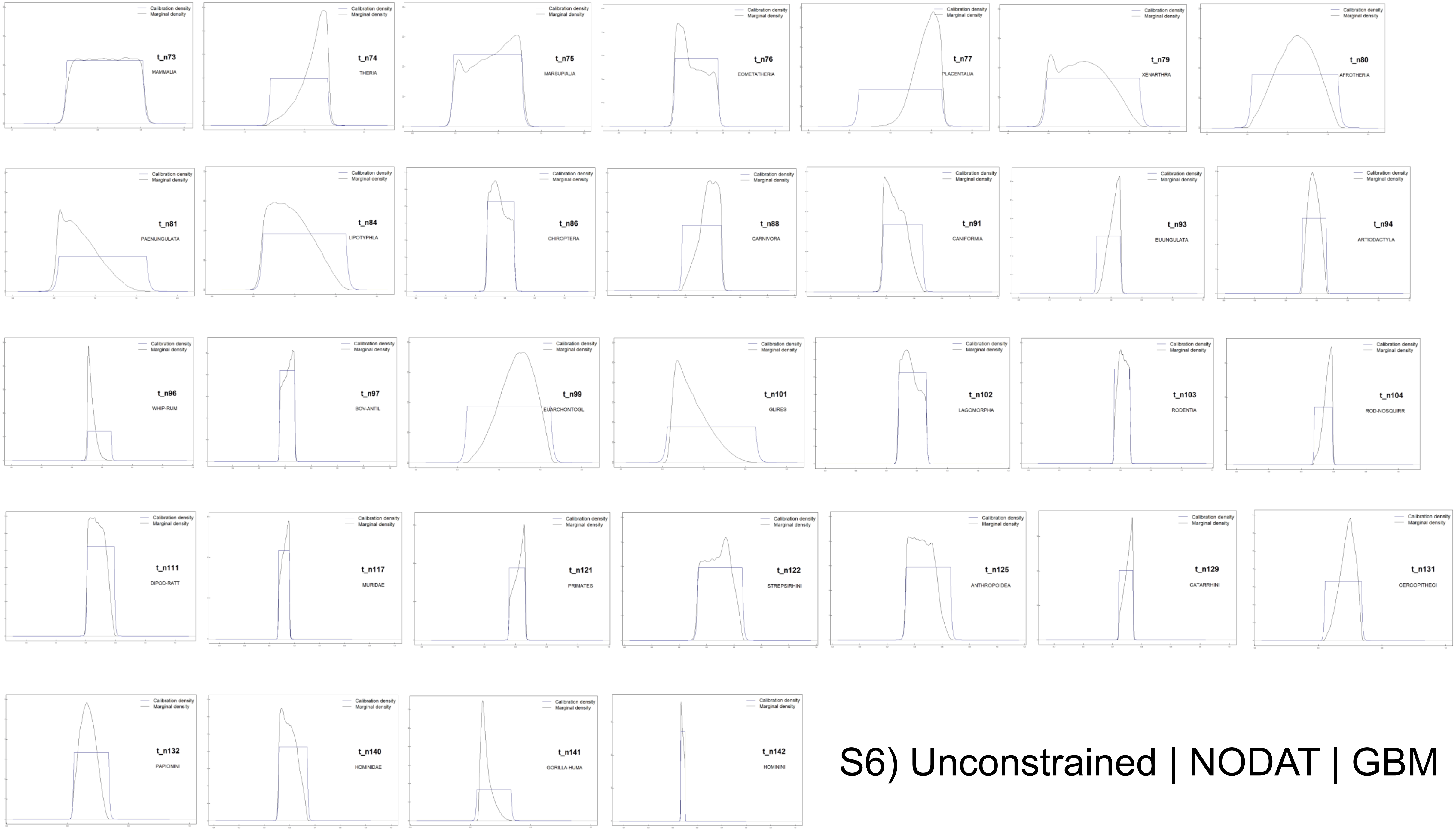

S6) Unconstrained | NODAT | GBM

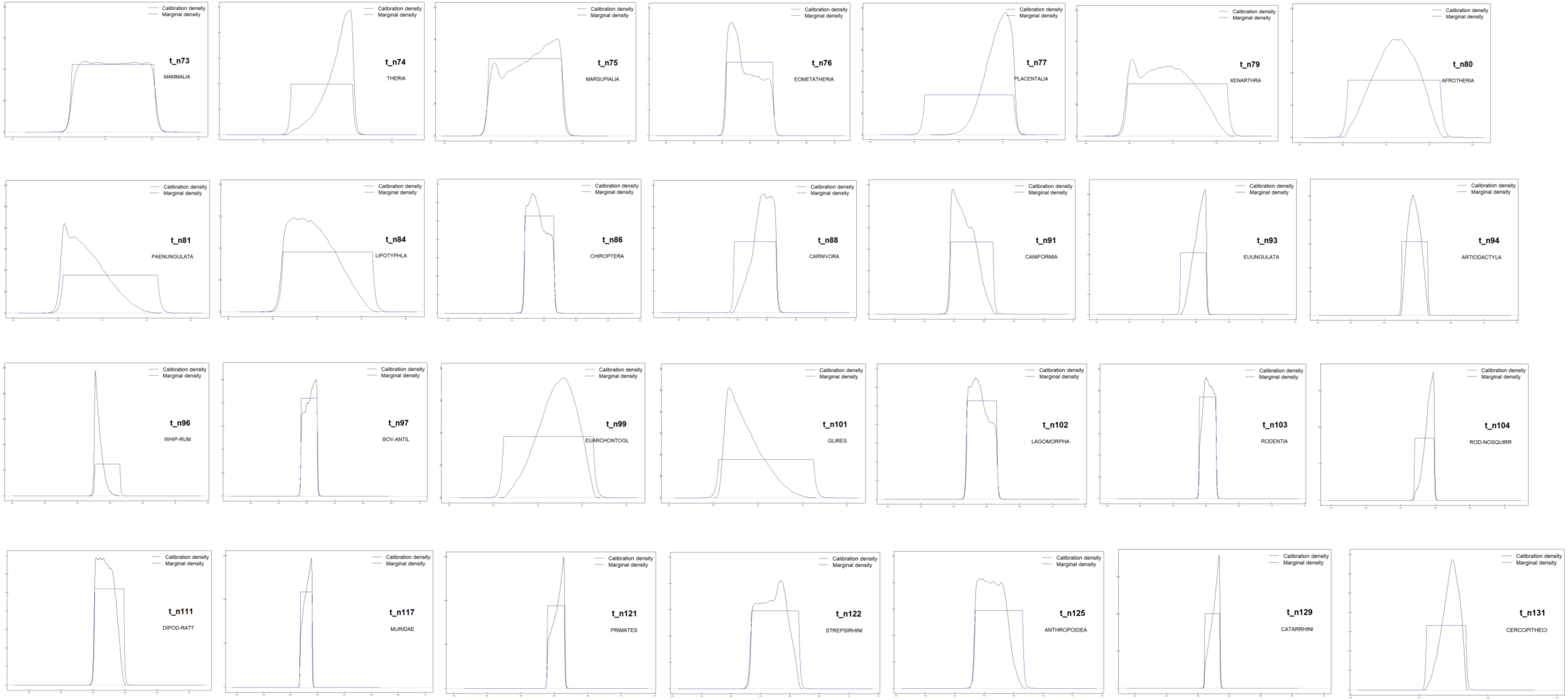

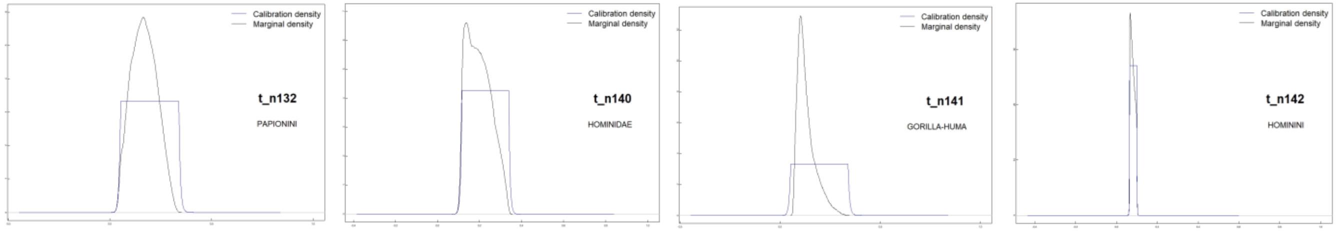

S7) Unconstrained | NODAT | ILN
