# Supplementary material for: On the origin of animals and placental mammals: a critique of literalist readings of the fossil record
Source: Mol Biol Evol. 2026 Aug 19;43(9):msag211. doi: 10.1093/molbev/msag211 (PMC13543917; doi:10.1093/molbev/msag211)
Supplement: Supplementary file 1 [file msag211_supplementary_data.zip › Supp_Fig_S8-S11_Mammals_rategrams.pdf]

S8) Post-K-Pg | GBM

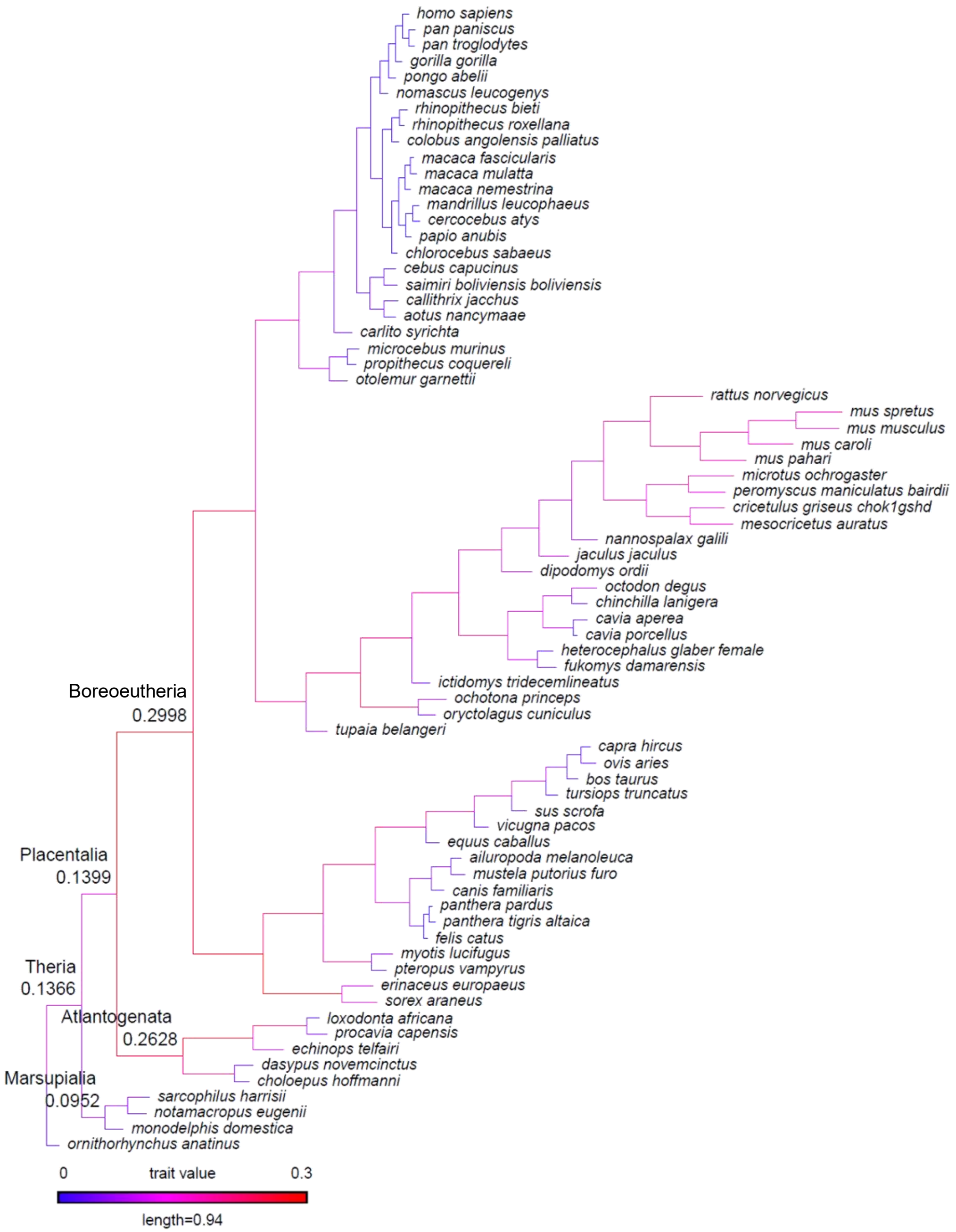

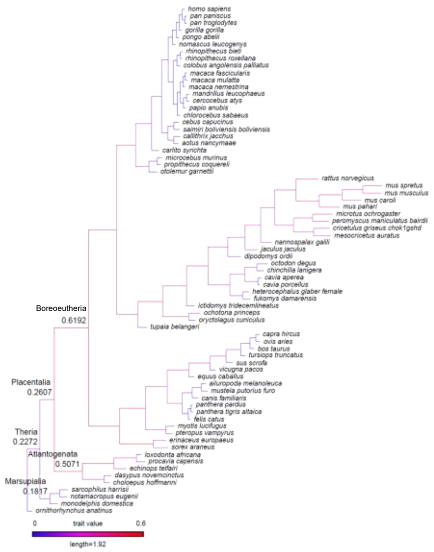

Partition 1

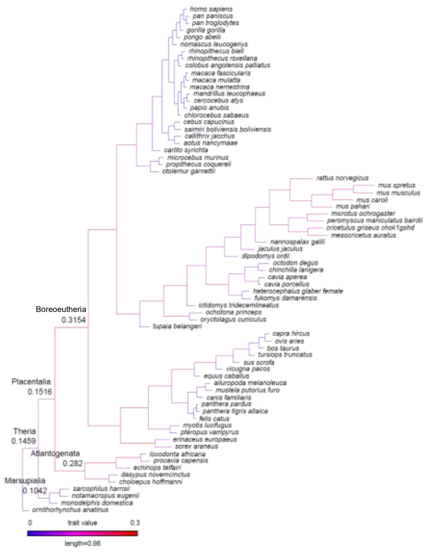

Partition 2

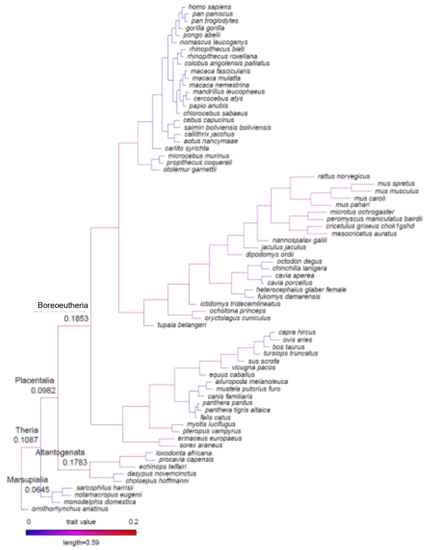

Partition 3

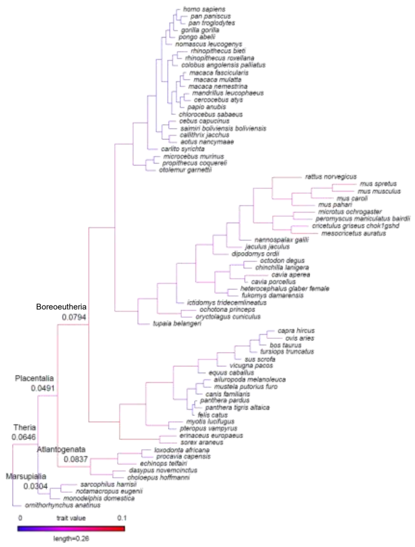

Partition 4

S9) Post-K-Pg | ILN

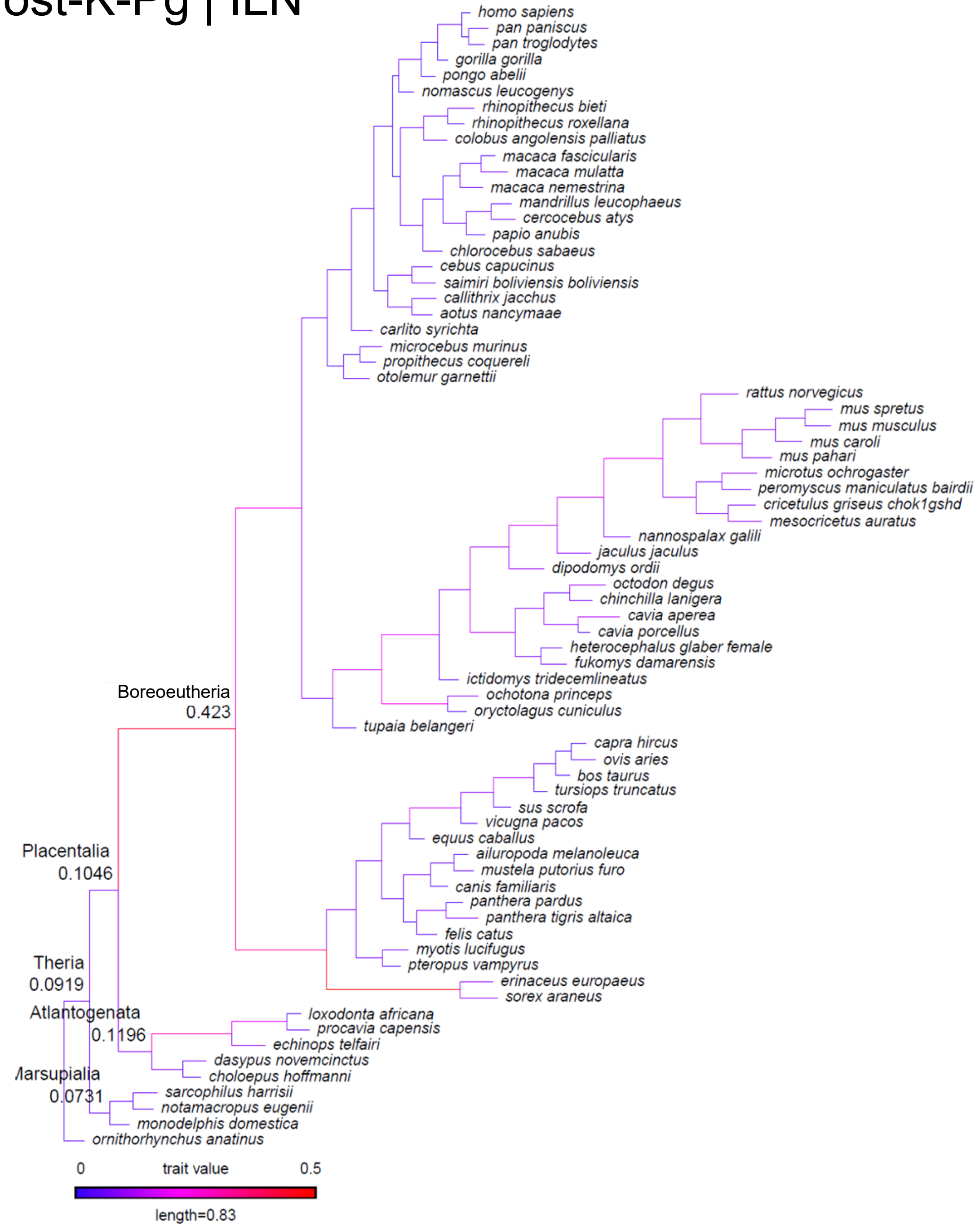

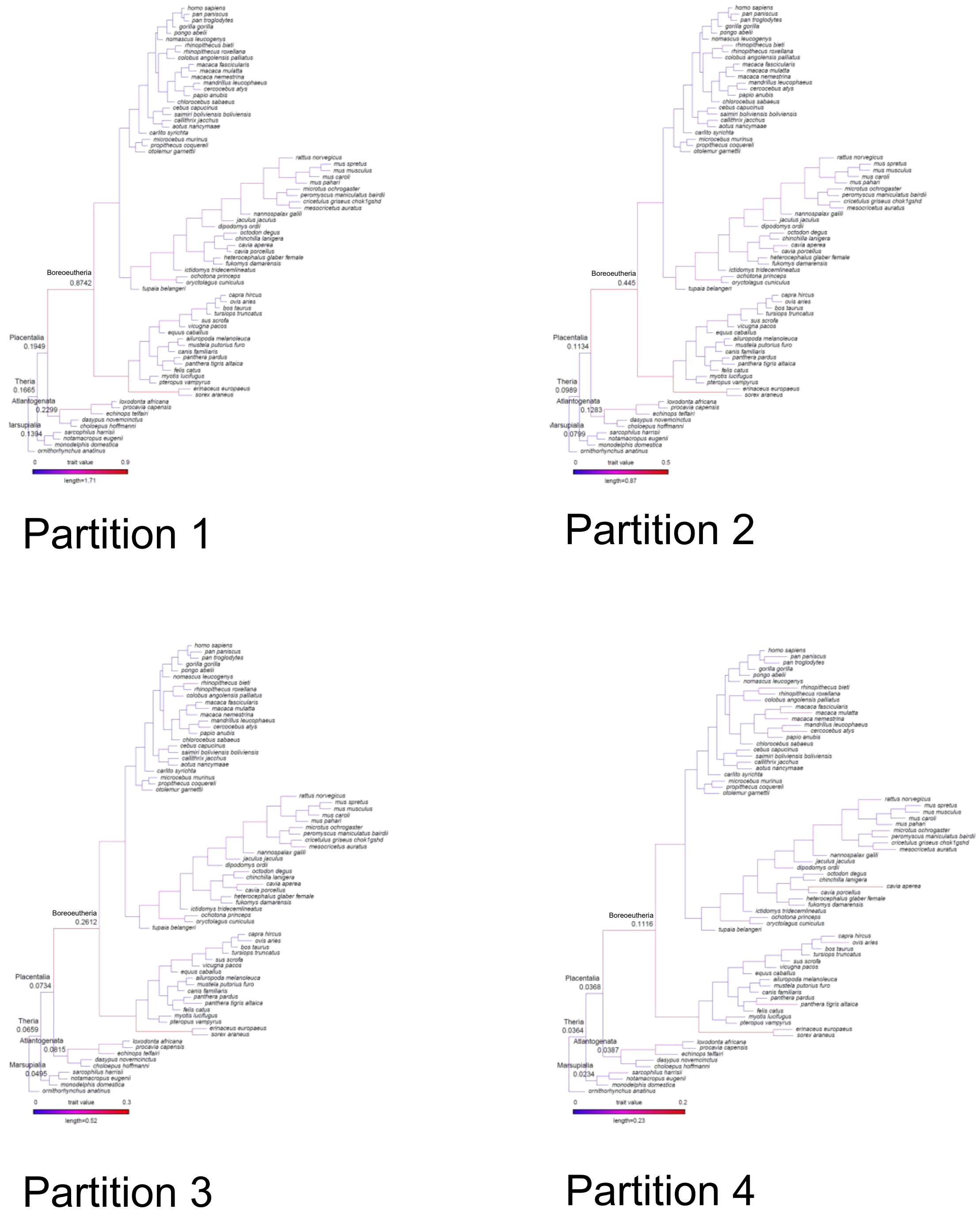

S10) Unconstrained | GBM

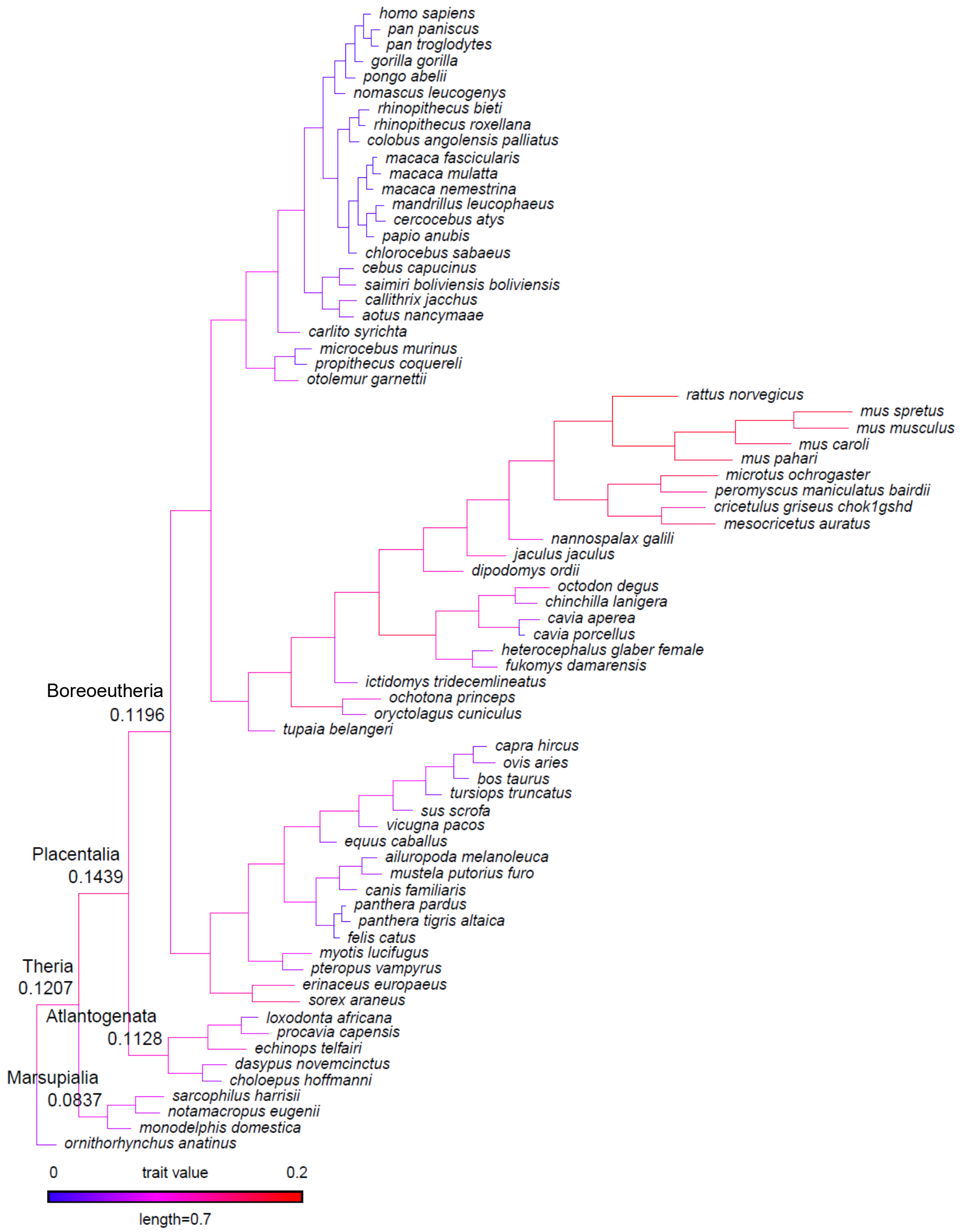

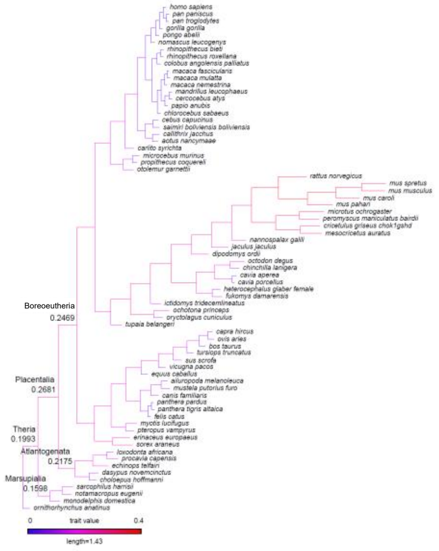

Partition 1

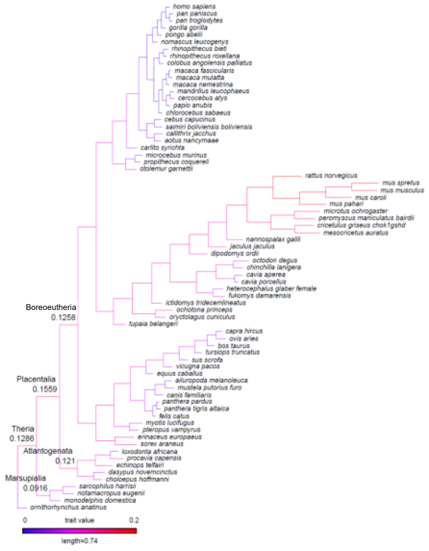

Partition 2

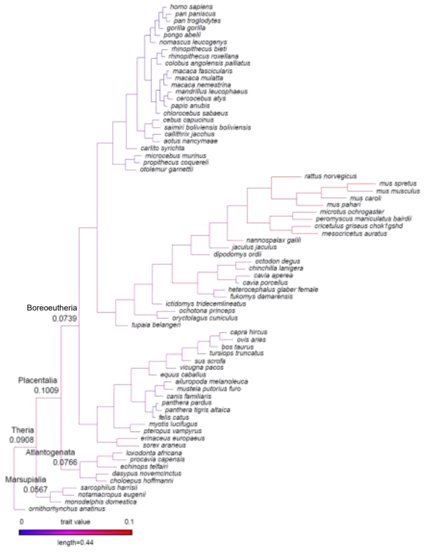

Partition 3

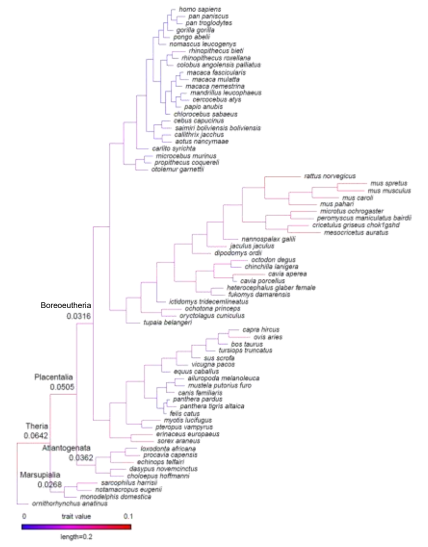

Partition 4

S11) Unconstrained | ILN

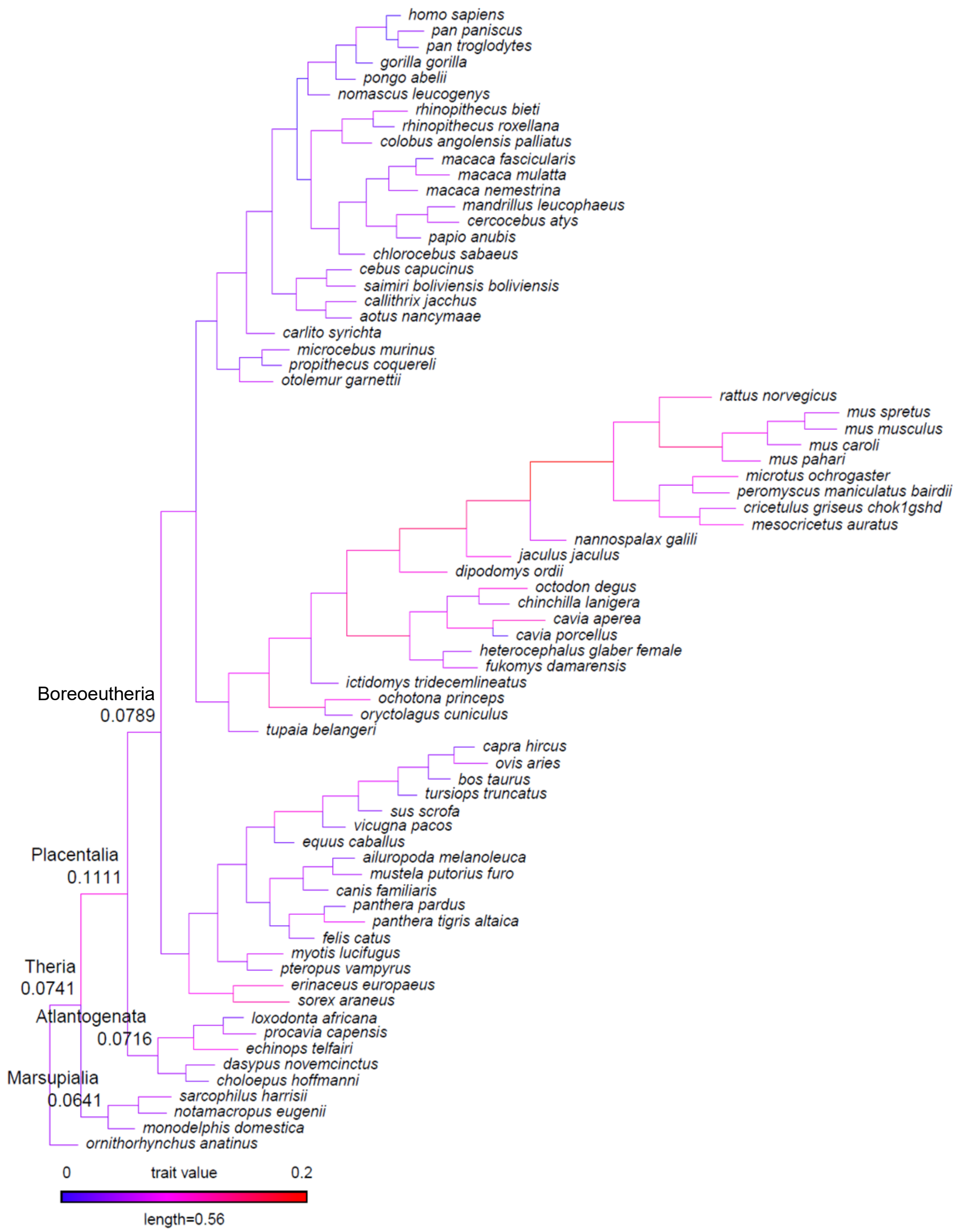

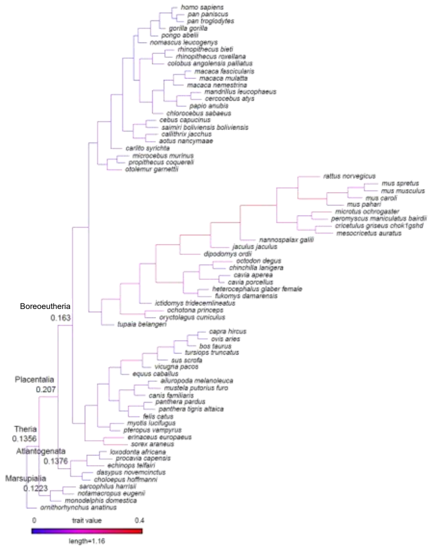

Partition 1

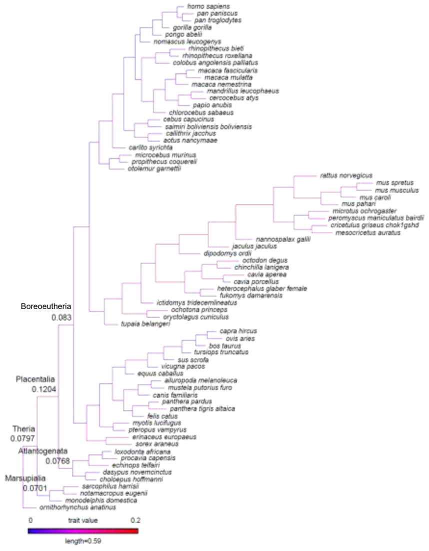

Partition 2

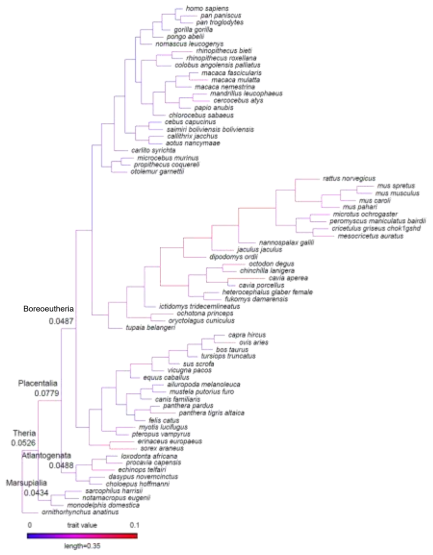

Partition 3

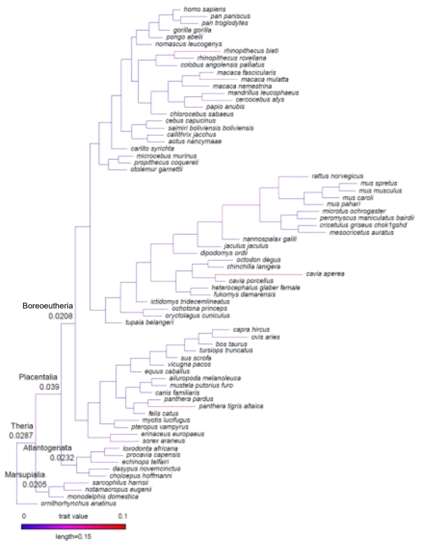

Partition 4
